# Asymmetric Organocatalytic Addition of Malononitrile to Trifluoromethyl Arylketimines: A Viable Entry to Chiral α-CF_3_ Quaternary Aminoesters

**DOI:** 10.3390/molecules31010141

**Published:** 2025-12-31

**Authors:** Milena Ivkovic, Francesca Franco, Sergio Rossi, Sara Ferrario, Alessandra Puglisi, Maurizio Benaglia

**Affiliations:** 1 Faculty of Pharmacy, University Business Academy, Heroja Pinkija 4, 21101 Novi Sad, Serbia; milena.ivkovic@ffns.ac.rs; 2 Dipartimento di Chimica, Università degli Studi di Milano, Via Golgi 19, 20133 Milano, Italy; sergio.rossi@unimi.it (S.R.); sara.ferrario@unimi.it (S.F.); alessandra.puglisi@unimi.it (A.P.)

**Keywords:** ketimines, organocatalysis, quaternary aminoesters, chiral iminophosphoranes, fluorinated aminoesters

## Abstract

In the present study the stereoselective addition of malononitrile to trifluoromethyl arylketimines promoted by chiral iminophosphoranes was investigated. A panel of structurally diverse enantiopure bifunctional superbases, which include thiourea or squaramide unit and a basic site connected by a chiral scaffold, was tested in the asymmetric organocatalytic reaction, to afford an adduct featuring a quaternary stereocenter, in up to a 87/13 enantiomeric ratio. The product was then converted in a single step transformation into the corresponding enantioenriched α-CF_3_ substituted quaternary aminoester, without any loss of stereochemical integrity. The absolute configuration of the final product was established by chemical correlation of the chiral compound with a known molecule. Preliminary computational studies were performed in order to elucidate the reaction mechanism and rationalize the stereochemical outcome of the reaction.

## 1. Introduction

Chiral organosuperbases remove protons from acidic pronucleophiles to produce a reactive nucleophile. The relevant protonated catalysts may engage effectively with the reactants via hydrogen bonding or electrostatic interaction [1]. Organosuperbases are usually defined by their excellent solubility in organic solvents, notable tunability, and the ability to broaden the reaction scope to challenging substrates with elevated pKa values. Moreover, chiral organobase catalysts with an additional functional group alongside the basic site can exhibit dual functionalization, simultaneously activating both pronucleophiles and electrophiles [2].

In this context, enantiopure bifunctional iminophosphoranes have found wide application in several transformations. In 2013 Dixon reported novel acyclic amino acid derived bifunctional iminophosphorane organocatalysts featuring a thiourea unit [3]. The high activity of the catalysts was demonstrated, among others, in the Mannich reaction of nitromethane to imines, direct aldol addition of aryl ketones to α-fluorinated ketones [4], 1,4-addition reactions (enantioselective sulfa-Michael to α-substituted acrylate esters [5,6], enantioselective cyclohexadienone desymmetrization [7], and synthesis of alkylidenecyclopropanes [8].

Following our interest in the synthesis of enantiopure fluorinated amines [9], we have recently reported a very efficient enantioselective organocatalytic addition of nitromethane to trifluoromethyl aryl ketimines promoted by electron-rich bifunctional iminophosphoranes, in up to 95% *e*.*e*. [10].

We wish to report here the stereoselective addition of malononitrile to trifluoromethyl arylketimines promoted by chiral iminophosphoranes. The addition products could be easily converted to enantioenriched α-CF_3_ substituted quaternary aminoesters, without any loss of stereochemical integrity [11].

## 2. Results and Discussion

### 2.1. Organocatalysts Synthesis

**Figure 1:**
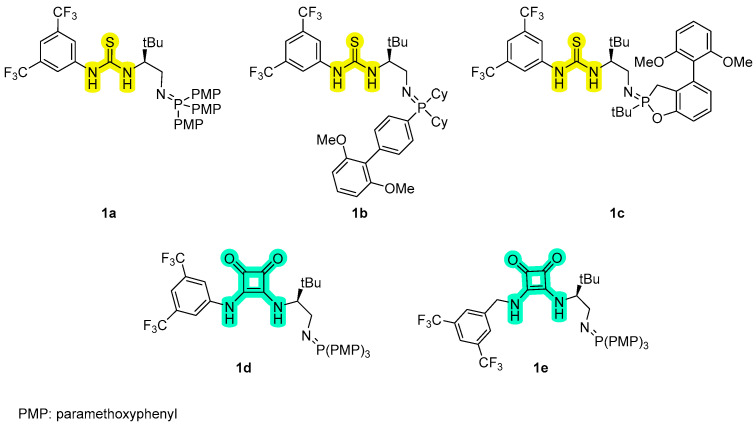
Selected chiral bifunctional iminophosphorane organocatalysts (BIMPs).

Taking advantage of their structural diversity and the tunability of both the basic and the hydrogen-bonding groups, a small library of bifunctional iminophosphorane (BIMPs) catalysts **1a**–**e** were synthetized. The basicity of the iminophosphorane unit was systematically tuned by the choice of phosphines, using tris(4-methoxypenyl)phosphine (P(PMP)_3_), 2-Dicyclohexylphosphino-2′,6′-dimethoxybiphenyl (SPhos) and 3-(tert-butyl)-4-(2,6-dimethoxyphenyl)-2,3-dihydrobenzo[d][1,3]oxaphosphole (rac-BIDIME), as representative partners. Thiourea- and squaramide-based H-bond donors were also explored as structural variations in the hydrogen-bond donor unit (Figure 1).

**Scheme 1:**
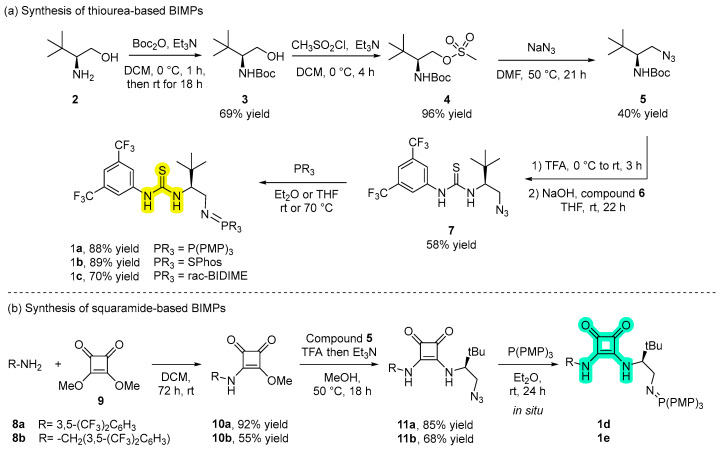
Synthetic pathways for the synthesis of (**a**) thiourea- and (**b**) squaramide-based BIMPs.

The synthetic routes to access the thiourea- and squaramide-based organocatalysts follow different pathways. In the case of thiourea based-BIMPs, catalysts **1a**–**c** were prepared from commercially available l-*tert*-leucinol **2** according to the literature procedure [10]. As shown in Scheme 1a, the free amino group of l-*tert*-leucinol was Boc protected to give carbamate **3**. Subsequent activation of the hydroxyl group with methanesulfonyl chloride results in the formation of mesylate **4**, which then underwent nucleophilic substitution with NaN_3_ to give the corresponding azide **5**. Deprotection of **5** with TFA, followed by treatment with aqueous NaOH and with 3,5-bistrifluoromethyl phenyl isothiocyanate **6**, leads to the formation of organoazide **7**, a common intermediate for the synthesis of catalysts **1a**–**c**. Intermediate **7** was then reacted in the Staudinger reaction with the desired phosphine, to afford the corresponding thiourea-based BIMP catalysts in good yields.

In contrast, squaramide-based BIMPs were accessed by condensation of the corresponding amine intermediates with commercially available dimethyl squarate under mild conditions (Scheme 1b). Due to stability issues, iminophosphorane catalysts **1d**–**e** were not isolated but generated in situ from stable precursors **11a**–**b** during the asymmetric addition of malononitrile to trifluoromethyl aryl ketimines. 3,5-bis(trifluoromethyl)aniline **8a** and 3,5-bis(trifluoromethyl)benzylamine **8b** were reacted with 3,4-dimethoxy-3-cyclobutene-1,2-dione **9** to afford the corresponding derivatives **10a**–**b** in good to excellent yields. At this point, a treatment with previously synthetized organoazide **5** afforded the desired stable precursors **11a**–**b** which were converted in situ into the desired organocatalysts **1d**–**e** by reaction with P(PMP)_3_ [12].

### 2.2. N-Boc Aryl Trifluoromethyl Ketimines Synthesis

**Scheme 2:**
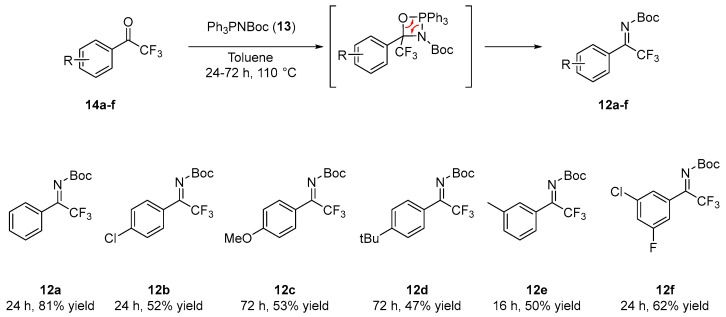
Synthesis of a small library of *N*-Boc aryl trifluoromethyl ketimines.

*N*-Boc trifluoromethyl ketimines **12a**–**f** where synthesized via a [2+2] cycloaddition of *N*-Boc-imino-(triphenyl)-phosphorane **13** with commercially available trifluoromethyl ketones **14a**–**f**, performed in toluene under reflux. The resulting four-membered heterocyclic intermediate easily undergoes reverse [2+2] cycloaddition to yield the target ketimines in 24–72 h [13]. Six ketimines exhibiting different electronic and steric properties where then prepared in 47–81% yields (Scheme 2).

### 2.3. Enantioselective Mannich Addition of Malononitrile to N-Boc Aryl Trifluoromethyl Ketimine

Having synthesized the required precursors, we investigated the enantioselective addition of malononitrile **15** to *N*-Boc aryl trifluoromethyl ketimine **12a**, selected as model compound, for the preparation of enantioenriched dicyano adduct **16a**. The addition product **16a** was then converted into the high-value α-trifluoromethyl α-amino esters **17a** by reaction with magnesium bis(monoperoxyphthalate) hexahydrate (MMPP) [14], for which the enantiomeric excesses were determined. Reactions were performed on a 0.2 mmol scale of ketimine using 2 equiv. of malononitrile, 10 mol% of BIMP organocatalyst at 0 °C in 0.75 mL of the desired solvent. Selected results are reported in Table 1 (see Supporting Information for further details).

Using toluene as the solvent, we first evaluated the influence of the basic moiety of the bifunctional organocatalyst. Catalysts **1a**, **1b**, and **1c**, bearing different iminophosphorane residues, afforded the desired product **16a** in comparable yields (76–85%) and with enantioselectivities up to 56% *e*.*e*. (entries 1–3). In contrast, with catalysts **1d** and **1e** compound **16a** was obtained in high yields but in racemic form, indicating that their structural features are not suitable for effective chiral discrimination in this transformation (entries 4–5). Since catalyst **1a** provided the most promising results, it was selected as catalyst of choice for the following screening. Different solvents and temperatures were then investigated. Ethers such as THF, MTBE, and Me-THF (entries 6–8) lead to the formation of desired product with slightly reduced yields (63–68%) but significantly improved enantiomeric excesses (74% *e*.*e*. in THF and 72% *e*.*e*. in MTBE and Me-THF). In contrast, chlorinated and polar aprotic solvents such as DCM and CH_3_CN (entries 9–10) provide good yields but very low enantioselectivities. Reaction performed in dioxane at room temperature (entry 11) afforded compound **16a** in moderate yield and enantioselectivity, whereas an attempt performed in THF at −20 °C for 96 h provided the desired product in 72% yield without improvement of the enantioselectivity (74% *e*.*e*. entry 12).

**Scheme 3:**
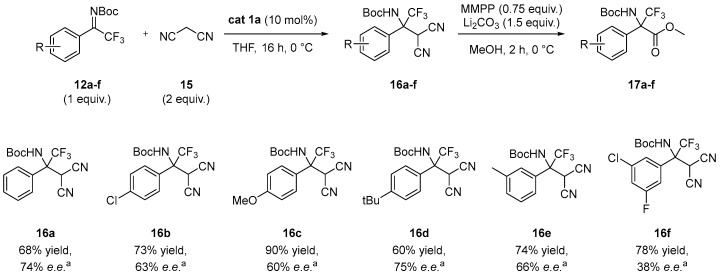
Ketimines scope in the enantioselective Mannich addition. ^a^ Enantiomeric excesses were determined by HPLC analysis of corresponding α-amino ester **17** on chiral stationary phase.

Having identified the best reaction conditions (Table 1, entry 7), the substrate scope of ketimines **12a**–**f** in the organocatalytic enantioselective addition was investigated (Scheme 3).

*Para*-substituted ketimines **16b**–**d** bearing either electron-withdrawing or electron-donating substituents were well tolerated, providing the corresponding adducts **16a**–**d** in yields of up to 90% (compound **16c**) and enantioselectivities of up to 75% (compound **16d**). When *meta*-substituted ketimine **12e** was employed, product **16e** was isolated in 74% yield and 66% *e*.*e*. Interestingly, electron poor disubstituted ketimine **12f** was also successfully engaged in the enantioselective Mannich addition, affording the corresponding product **16f** in 78% yield and 38% *e*.*e*.

**Scheme 4:**
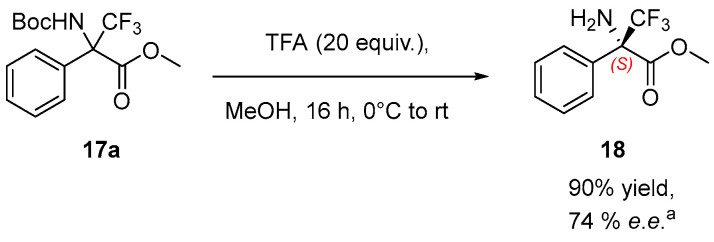
Assignment of the absolute configuration of compound **17a**. ^a^ Enantiomeric purity was assigned by comparison of known optical purity value.

To assign the absolute configuration of the reaction product, compound **17a** was converted into compound **18** in 90% yield via amine deprotection without erosion of the enantiomeric excess (Scheme 4). According to the literature it was not possible to determine the enantiomeric excess of compound **18** by chiral GC or HPLC; therefore, the enantiomeric purity was assigned by comparison with a reported optical purity value [15]. The optical purity of compound **18** was determined by polarimetric analysis and compared with the reported literature values, confirming that the compound adopt the (*S*)-configuration [15].

**Scheme 5:**
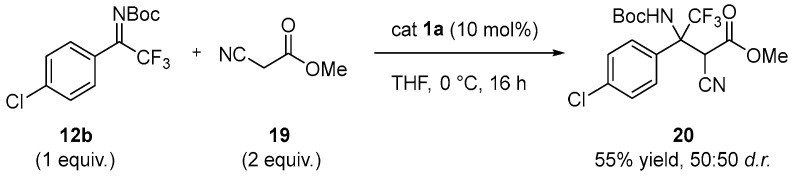
Organocatalytic addition of methyl 2-cyanoacetate **19** to ketimine **12b**.

To further evaluate the feasibility of the transformation and to access more synthetically demanding, highly functionalized compounds, the reaction was extended to the more challenging nucleophile methyl 2-cyanoacetate **19** in combination with ketimine **12b**. Gratefully, under the optimized reaction conditions, the corresponding product **20** was obtained in 55% yield as a 1:1 mixture of diastereoisomers (Scheme 5). Further studies will be required to determine the enantiomeric excess.

### 2.4. Computational Analysis. pKa Values

In order to rationalize the stereochemical outcome of this transformation, computational analysis was performed. At first a DFT investigation of the acid/base properties of catalyst **1a** was investigated. It is known that the pKa values of widely used chiral bifunctional dialkylamino and cinchona-derived (thio)urea organocatalysts fall within the range of 8.5–19.6 in DMSO [16], with the strongest acidic example being Schreiner’s thiourea [17,18], whereas one of the least acidic examples is the Jacobsen-List organocatalysts [19,20].

Although bifunctional iminophosphoranes are often described as superbases catalysts due to their pronounced reactivity in bifunctional activation [1,2], no experimental determinations of BIMPs intrinsic acid-base profile are currently available. Only a few examples related to the determination of iminophosphorane bases pKa have been reported [3,7]. For this reason, we computed the micro-pKa and pK_BH_^+^ values of catalyst **1a**, considering both its neutral and protonated forms, in order to clarify its acid–base profile and its potential role in the catalytic activation of different substrates.

Quantum chemical methods have been widely employed for pKa predictions, using either direct methods based on Born–Haber cycles [21], or indirect approaches, commonly referred to as the “isodesmic method [22].” Direct methods provide results usually aligned with experimental measurements but require high computational costs, especially when explicit solvation models are employed, making them impractical for larger systems. Indirect isodesmic methods, on the other hand, are computationally less demanding but require the use of an experimental pKa value from a reference acid-base reaction involving species similar to those under investigation. In this approach, structural similarity is critical, since even small differences can lead to significant deviations in acidity, making the careful selection of an appropriately comparable reference a challenging task. Isodesmic method has been successfully applied to the determination of the acid-base profile of the iminophosphorane moiety of BIMPs (BH^+^ → B + H^+^ profile), using pKa of aniline as reference [23]; however, to the best of our knowledge, no pKa determination of the neutral structure has been computed.

**Scheme 6:**
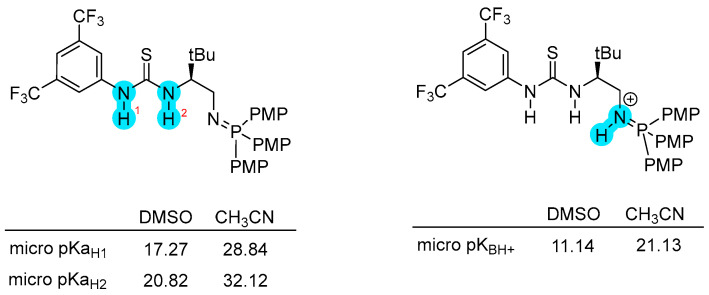
pKa estimations for catalyst **1a** in DMSO and CH_3_CN using the LFESR approach. All computations were performed at M06-2X/6-311++G (d,p) level of theory, and formic acid was used as a reference reaction.

To overcome the limitation of isodesmic approach, in 2022, Busch and co-workers reported an alternative and more general computational method able of predicting pKa values for a broad range of compounds across different solvents, which does not require being referred to a known reference species. This method is based on experimental aqueous pKa values of known molecules combined with absolute potentials of the standard hydrogen electrode (SHE) in non-aqueous media (LFESR approach) [24]. Inspired by these developments, we adopted this computational strategy to estimate the acidities of catalyst **1a** in DMSO and CH_3_CN. Taking advantage of the LFESR approach, it was possible to estimate the proton solvation energy at a minimal computational cost. A summary of the pKa estimations is presented in Scheme 6. DFT calculations were performed using Gaussian 16, Revision C.01, the M06-2X functional and 6-311++G (d,p) basis set in combination with SMD solvation model since it offered the best combination of accuracy, reliability, and time consumption [24].

The computed acidity trends provide clear insight into the acid behavior of catalyst **1a** in different solvents. In both DMSO and CH_3_CN media, the N–H group of the thiourea bound to the 3,5-bis(trifluoromethyl)phenyl ring (N–H1) is consistently more acidic than the N–H group located proximal to the iminophosphorane unit (N–H2), as evidenced by systematically lower micro-pKa values (pKa_H1_ < pKa_H2_). This identifies N-H1 group as the most acidic site of the catalyst. Although the overall acidity of the catalyst is formally determined by the macroscopic pKa, the two micro-pKa values differ by more than two orders of magnitude, making the contribution of the less acidic site negligible for all practical purposes. The absolute pKa values, however, exhibit a pronounced solvent dependence: all micro-pKa values are substantially higher in CH_3_CN than in DMSO, reflecting the reduced ability of acetonitrile to stabilize charged species.

**Scheme 7:**
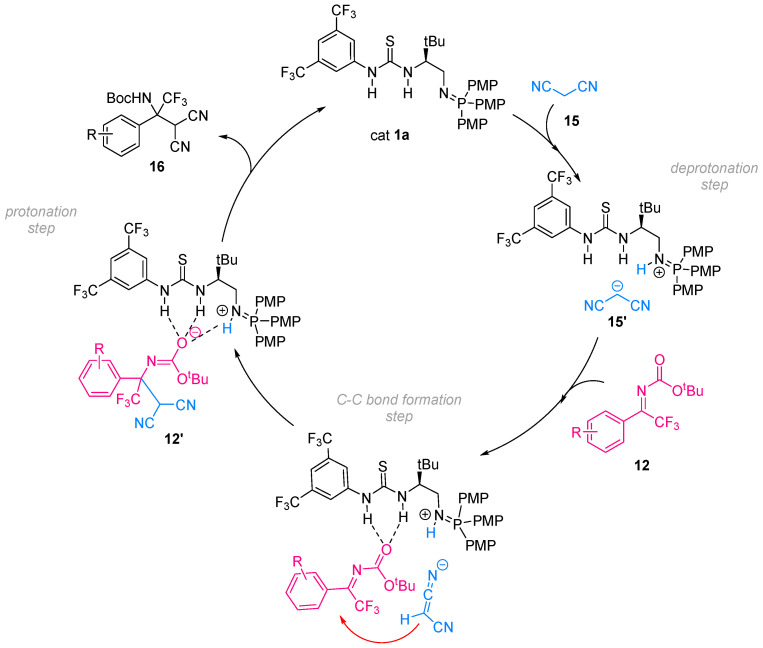
Proposed mechanism for the stereoselective addition of malononitrile to *N*-Boc ketimines promoted by BIMP catalyst **1a**.

Considering the acidity trends discussed above and based on previously reported stereoselective transformations promoted by this class of catalysts (ketimine nitro-Mannich reactions [10], sulfur–Michael additions [6], and conjugate additions to enone diesters [5]), a plausible reaction mechanism for the enantioselective addition of malononitrile **15** to ketimine **12a** was proposed (Scheme 7).

Following the initial deprotonation of malononitrile **15** by the basic iminophosphorane moiety, the resulting malononitrile anion **15**′ is engaged in a nucleophilic attack on the ketimine **12**, which is simultaneously activated through dual hydrogen-bond coordination between the thiourea unit of the catalyst and the *N*-Boc protecting group of the substrate. This is consistent with the fact that the most acidic moiety of the catalyst is involved in hydrogen-bond formation with the ketimine, whereas the basic site, once protonated, interacts via electrostatic interaction with the malononitrile anion. It should be noted that the deprotonation step and nucleophilic attack could occur in a concerted manner or through discrete, sequential steps. However, according to this proposed catalytic cycle, the reactive partners are aligned within a well-defined chiral environment, favoring the formation of a new C–C bond in a stereoselective manner. After that, a proton transfer from the protonated iminophosphorane to the negatively charged *N*-Boc-protected intermediate **12**′ occurs, leading to the formation of the desired *N*-Boc-protected fluorinated dicyano compound **16** and regenerating the free BIMP catalyst which is able to re-enter the catalytic cycle.

### 2.5. Computational Analysis

In order to confirm this hypothesis, preliminary computational studies were performed on the transition states involved in this transformation. For this purpose, calculations were performed considering the ketimine **12a** and catalyst **1a**, which has been shown to promote the formation of the desired compound (*S*)-**16a** with 74% *e*.*e*. To comprehensively investigate all relevant transition states, several key structural and mechanistic factors were considered. These included the *E*/*Z* configuration of ketimine **12a**, the conformational flexibility of the thiourea unit (capable of adopting either s-*cis* or s-*trans* arrangements) and the hydrogen-bonding interactions within **12a** and three distinct acidic sites of the catalyst (namely the N-H1 and N-H2 groups of the thiourea moiety and the P=NH^+^ group). Additionally, the approach of the nucleophilic malononitrile anion to either the *re* or *si* face of the imine becomes particularly relevant for the final configuration of the product.

**Scheme 8:**
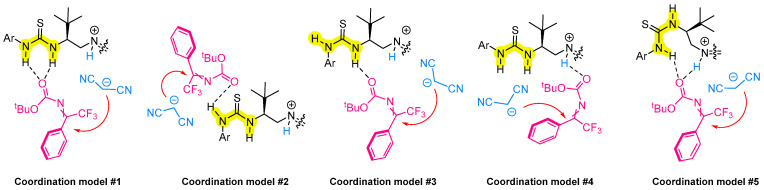
Five possible coordination models proposed for the activation of ketimine **12a** by catalyst **1a**.

Considering all these aspects, at least five different coordination models can be proposed, (Scheme 8) considering all of them and taking into account the *E*/*Z* configuration of the imine as well as the nucleophilic attack on the *si*/*re* face, four transition states can be identified for each model, resulting in a total of 20 transition states.

Initial conformational geometries were obtained by Monte Carlo conformational analysis performed with molecular mechanics calculations using the OPLS4 force field [25] of the Macromodel package in the Schrodinger suite [26]. These structures were then fully optimized in vacuo using the PM6 semiempirical method of the Gaussian package [27]. This level of theory was chosen as a compromise between computational cost and the need to explore the large number of transition states arising from the five proposed coordination models, enabling a rapid and systematic investigation of all relevant transition state geometries. Harmonic vibrational calculations were also performed to confirm that the optimized structures correspond to first-order saddle points, each exhibiting a single imaginary frequency. All five different coordination models where analyzed, and in the notation used for describing transition states, (s-*cis*)/(s-*trans*) refers to the conformation of the thiourea unit, *E*/*Z* indicates the configuration of the imine, and *re*/*si* denotes the face of the imine exposed to nucleophilic attack by malononitrile.

A detailed analysis of the possible 20 transition states is available in the Supporting Information. Although additional computational studies at higher level are necessary, from the preliminary investigation, considering that imine *Z* is known to be more stable, it resulted that low energy TS could be found with both coordination modes one and five, where a clear preference for nucleophilic attack on the *si* face of the *Z*-configured imine is present, in line with the experimental observations. While the results provide a useful preliminary overview of the relative stabilities of the 20 transition states across the five proposed coordination models, they should be interpreted with caution. The absolute values and relative ordering may change at a higher level of theory and when solvent effects are considered, which are known to influence both the stabilization of transition states and the stereochemical outcome. Therefore, the present data are most useful for identifying trends and for selecting the most relevant transition states to be further optimized and evaluated using more accurate computational methods.

## 3. Material and Methods

Reactions were carried out under a positive pressure of nitrogen and dry solvents were used. Reactions were monitored by thin layer chromatography (TLC) on Macherey-Nagel (Düren, Germany) pre-coated silica gel plates (0.25 mm) and visualized by UV irradiation at 254 nm. Whenever necessary, a ninhydrin solution or a permanganic solution was used as stains for developing TLC plates. Flash chromatography was performed on standard flash column chromatography on Merck (Darmstadt, Germany). silica gel 60 (particle size: 0.04–0.063 mm). Hexane, pentane, ethyl acetate (EtOAc), dichloromethane (DCM), methanol (MeOH), and diethyl ether (Et_2_O) were used as standard eluent solvents.

^1^H NMR, ^13^C NMR and ^19^F NMR spectra were recorded at 25 °C on Bruker Avance spectrometers (300 MHz, 75 MHz, 282 MHz) or NEO 400 MHz (Bruker, Billerica, MA, USA) (400 MHz for ^1^H NMR, 101 MHz for ^13^C NMR). Deuterated solvents acquired from Sigma-Aldrich (Steinheim, Germany). were used as supplied. The spectra were recorded in ppm using the solvent peak as a reference for ^1^H and ^13^C NMR spectra (7.26, 77.16 for CDCl_3_).

High-resolution mass spectra (HRMS) were obtained from the Unitech COSPECT center, University of Milan and performed on a Q-TOF Synapt G2-Si (Waters, Milford, MA, USA) using an Acquity UPLC I-Class photodiode array (PDA) detector (Waters, Milford, MA, USA).

Enantiomeric excess determinations were performed with an Agilent (Santa Clara, CA, USA) Instrument Series 1100, using a Chiralpak AD or a Lux Phenomenex 3 µm Amylose-1 as column (eluent: *n*-hexane/isopropanol 95:5 according to the sample, flow rate as specified).

XYZ geometries of transition states are available as a separate supporting material on a Dataverse repository.

### 3.1. General Procedure for the Synthesis of N-Boc Trifluoromethyl Aryl Ketimines (***12a**–**f***)

According to the literature procedure [13], *N*-Boc-imino-(triphenyl)-phosphorane **13** (2 equiv.) was added to a solution of 300 mg (1 equiv.) of the corresponding commercially available trifluoroacetophenone **14a**–**f** in dry toluene (5 mL). The reaction mixture was heated and stirred for 24–72 h (depending on starting ketone) at 110 °C. The reaction was monitored by TLC and/or ^1^H NMR in CDCl_3_. After the consumption of starting ketone, the reaction was cooled down to room temperature and toluene was removed under reduced pressure. The residue was purified by silica gel column chromatography (*n*-Hexane/AcOEt from 98:2 to 90:101) to afford ketimines **12a**–**f** in modest to good yields. All the analytical data are in agreement with the literature [10,28].

### 3.2. General Procedure for the Enantioselective Synthesis of ***15a**–**f***

A 10 mL Schlenk tube under nitrogen was charged with ketimine **12a**–**f** (0.2 mmol, 1 equiv., 0.27 M in THF) and 14.6 mg of iminophosphorane catalyst **1a** (0.02 mmol, 0.1 equiv.). The reaction mixture was cooled down to 0 °C, and 26.4 mg of malononitrile **15** (0.4 mmol, 2 equiv.) were added. The reaction mixture was stirred for 16 h at 0 °C, then THF was removed under reduced pressure and the crude was purified by column chromatography on silica gel (*n*-Hexane/AcOEt from 100:0 to 90:10) to afford compounds **16a**–**f**.

### 3.3. General Procedure for Oxidative Decyanation of ***16a**–**f***

According to the literature [14], under nitrogen atmosphere, a flask was charged with compounds **16a**–**f** (1 equiv., 0.1 M in dry methanol) and dry methanol at 0 °C. Magnesium monoperoxyphthalate hexahydrate (MMPP, 0.75 equiv.), and Li_2_CO_3_ (1.5 equiv.) were added to the stirring solution. The reaction mixture was stirred for 2 h at 0 °C. The reaction was stopped and quenched by water and extracted with DCM (3 × 5 mL). The combined organic layers were dried over anhydrous Na_2_SO_4_, filtered, and concentrated under reduced pressure to afford quaternary amino esters **17a**–**f**.

Determination of enantiomeric excesses was conducted by chiral HPLC (column: Chiralpak AD, Lux Phenomenex 3 µm Amylose-1; eluent: *n*-hexane/isopropanol 95:5, flow rate 1 mL/min).

## 4. Conclusions

In conclusion, a new mild and efficient methodology for the synthesis of α-trifluoromethyl α-aminoesters was disclosed. Good yields, up to 90%, and enantioselectivities up to 75% were achieved. The methodology was applied to a small library of ketimines and a very preliminary attempt of using methyl 2-cyanoacetate as nucleophile is reported. The absolute configuration of the reaction product was assigned as (*S*) enantiomer, based on literature data. Preliminary computational investigation on the pKa values of the bifunctional iminophosphorane [29,30] catalysts and studies of the possible transition states of the reaction were also performed, thus opening the way to a better understanding of the mode of action of those bifunctional organosuperbases and to a rationalization of the observed stereochemical outcome of the organocatalytic transformation.

## Figures and Tables

**Table 1:** Optimization of reaction conditions for the enantioselective addition of malononitrile **15** to ketimine **12a**.

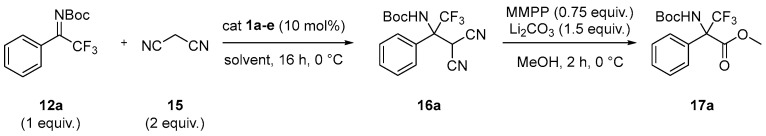				
Entry	Catalyst	Solvent	Yield (%)	*e*.*e*. (%) ^a^
1	**1a**	Toluene	85	52
2	**1b**	Toluene	72	50
3	**1c**	Toluene	76	56
4	**1d**	Toluene	72	0
5	**1e**	Toluene	90	0
6	**1a**	THF	68	74
7	**1a**	MTBE	67	72
8	**1a**	Me-THF	63	72
9	**1a**	DCM	83	57
10	**1a**	CH_3_CN	80	22
11 ^b^	**1a**	Dioxane	62	64
12 ^c^	**1a**	THF	72	74

^a^ Determined by HPLC analysis of compound **17a** on chiral stationary phase. ^b^ Reaction performed at room temperature. ^c^ Reaction performed at −20 °C for 96 h.

## Data Availability

The original data presented in the study are openly available in Dataverse UNIMI at: https://doi.org/10.13130/RD_UNIMI/EG0U5H (accessed on 15 December 2025).

## Supplementary Materials

The following supporting information can be downloaded at: https://www.mdpi.com/article/10.3390/molecules31010141/s1 and comprises the detailed synthesis and characterization of the catalysts employed, the general experimental procedure for the enantioselective addition of malononitrile to ketimines, and the full spectroscopic data of all relevant compounds. See Schemes S1–S6; Tables S1–S6. References [31,32,33,34,35,36,37] are cited in the Supplementary Materials.
