# Supplementary material for: Asymmetric Organocatalytic Addition of Malononitrile to Trifluoromethyl Arylketimines: A Viable Entry to Chiral α-CF3 Quaternary Aminoesters
Source: Molecules. 2025 Dec 31;31(1):141. doi: 10.3390/molecules31010141 (PMC12787335; doi:10.3390/molecules31010141)
Supplement: Supplementary file 1 [file molecules-31-00141-s001.zip › molecules-4073340-supplementary.pdf]

Supplementary Material

Asymmetric organocatalytic addition of malononitrile to trifluoromethyl arylketimines: a viable entry to chiral α-CF_3 quaternary aminoesters

Milena Ivkovic[1], Francesca Franco[2,]*, Sergio Rossi[2], Sara Ferrario[2], Alessandra Puglisi[2] and Maurizio Benaglia[2,]*

[1]Faculty of Pharmacy, University Business Academy, Heroja Pinkija 4, 21101 Novi Sad, Serbia

[2] Dipartimento di Chimica, Università degli Studi di Milano, Via Golgi 19, 20133 Milano, Italy

Table of Contents

1. General information S3

2. Experimental Procedures and Characterization Data S4

2.1 Synthesis of BIMP catalysts (1a-1e) S4

2.2 Synthesis of *N*-Boc aryl trifluoromethyl ketimines (12a-f) S8

2.3 Enantioselective synthesis of compound 16a S9

2.4 Enantioselective synthesis of compounds 16a-f S10

2.5 Racemic synthesis of compounds 16a-f S12

2.6 Synthesis of quaternary α-amino esters (17af) S12

2.7 Assignment of the absolute configuration of compound 16a S15

2.8 Enantioselective synthesis of compound 20 .... S15

2.9 Racemic synthesis of compound 20 .... S16

3. NMR spectra S17

4. HPLC traces S43

5. Computational data S49

5.1 Micro-pKa determination of catalyst 1a S49

5.2 Transition state investigation S49

6. References S53

1. General information

Reagents were purchased at the highest commercial quality and used as received. Dry solvents were purchased with AcroSeal packaging and used without further purifications.

If not otherwise stated, reactions were carried out under a positive pressure of nitrogen (5 cm of mercury, or with a spring-loaded silicon oil bubbler set to 100 mbar) and dry solvents were used. Reactions were monitored by thin layer chromatography (TLC) on Macherey-Nagel pre-coated silica gel plates (0.25 mm) and visualized by UV irradiation at 254 nm. Whenever necessary, a ninhydrin solution (400 mg of ninhydrin, 200 mL of ethanol) or a permanganic solution (3 g potassium permanganate, 20 g sodium carbonate, 300 mL of deionized water, sodium hydroxide 5 % solution) were used as stains for developing TLC plates. Flash chromatography was performed on standard flash column chromatography on Merck silica gel 60 (particle size: 0.04–0.063 mm). Hexane, pentane, ethyl acetate (EtOAc), dichloromethane (DCM), methanol (MeOH), diethyl ether (Et_2O) were used as standard eluent solvents.

^{1}H NMR, ^{13}C NMR, and ^{19}F NMR spectra were recorded at 25 °C on Bruker Avance spectrometers (300 MHz, 75 MHz, and 282 MHz) or NEO 400 MHz (Bruker) (400 MHz for ^{1}H NMR, and 101 MHz for ^{13}C NMR). Deuterated solvents acquired from Sigma-Aldrich were used as supplied. The spectra were recorded in ppm using the solvent peak as a reference for ^{1}H and ^{13}C NMR spectra (7.26, 77.16 for $CDCl_3$). ^{1}H NMR data are reported as follows: chemical shift (ppm), multiplicity (s = singlet, br. s. = broad singlet, d = doublet, t = triplet, q = quartet, quint = quintet, sext = sextet, hept = heptet, dd = doublet of doublets, ddd = doublet of doublets of doublets, td = triplet of doublets, qd = quartet of doublets, m = multiplet), coupling constants (Hz), and numbers of protons. ^{19}F, ^{31}P NMR and ^{13}C NMR data are generally reported as follows: chemical shift (ppm). Structural assignments were made with additional information from gHSQC and gHMBC experiments.

High-resolution mass spectra (HRMS) were obtained from the Unitech COSPECT centre, University of Milan and performed on a Q-TOF Synapt G2-Si using an Acquity UPLC I-Class photodiode array (PDA) detector.

Enantiomeric excess determinations were performed with an Agilent Instrument Series 1100, using a Chiralpak AD or a Lux Phenomenex 3µm Amylose-1 as column (eluent: n-hexane/isopropanol 95:5 according to the sample, flow rate as specified).

XYZ geometries of transition states are available as a separate supporting material on a Dataverse repository.

2. Experimental Procedures and Characterization Data

2.1 Synthesis of BIMP catalysts (1a-1e)

Synthesis of BIMP catalyst precursor 7

The synthetic routes used to access the thiourea- and squaramide-based organo-catalysts follows different synthetic pathways. In the case of thiourea based-BIMPs, catalysts **1a-c** were prepared from commercially available L-tert-leucinol **2** according to literature procedures.[1-5]

Initially, according to a literature procedure,[2] the free amino group of L-tert-leucinol **2** was protected under standard Boc-protection conditions to give tert-butyl (*S*)-(1-hydroxy-3,3-dimethylbutan-2-yl)carbamate **3**. All the analytical data are in agreement to those reported.[2]

According to a literature procedure,[3] the subsequent activation of the hydroxyl group of carbamate **3** with methanesulfonyl chloride results in the formation of (*S*)-2-((tert-butoxycarbonyl)amino)-3,3-dimethylbutyl methanesulfonate **4**, which then underwent nucleophilic substitution with NaN_3 to give the corresponding azide **5**.[4] All the analytical data are in agreement to those reported.[3,5]

Deprotection of tert-butyl (*S*)-(1-azido-3,3-dimethylbutan-2-yl)carbamate **5** with TFA, followed by treatment with aqueous NaOH and with 3,5-bistrifluoromethyl phenyl isothiocyanate **6**, leads to the formation of (*S*)-1-(1-azido-3,3-dimethylbutan-2-yl)-3-(3,5-bis(trifluoromethyl)phenyl)thiourea **7**. All the analytical data are in agreement with the literature.[1,5]

The synthetized organoazide **7** serves as common intermediate for the synthesis of catalysts **1a-c**.

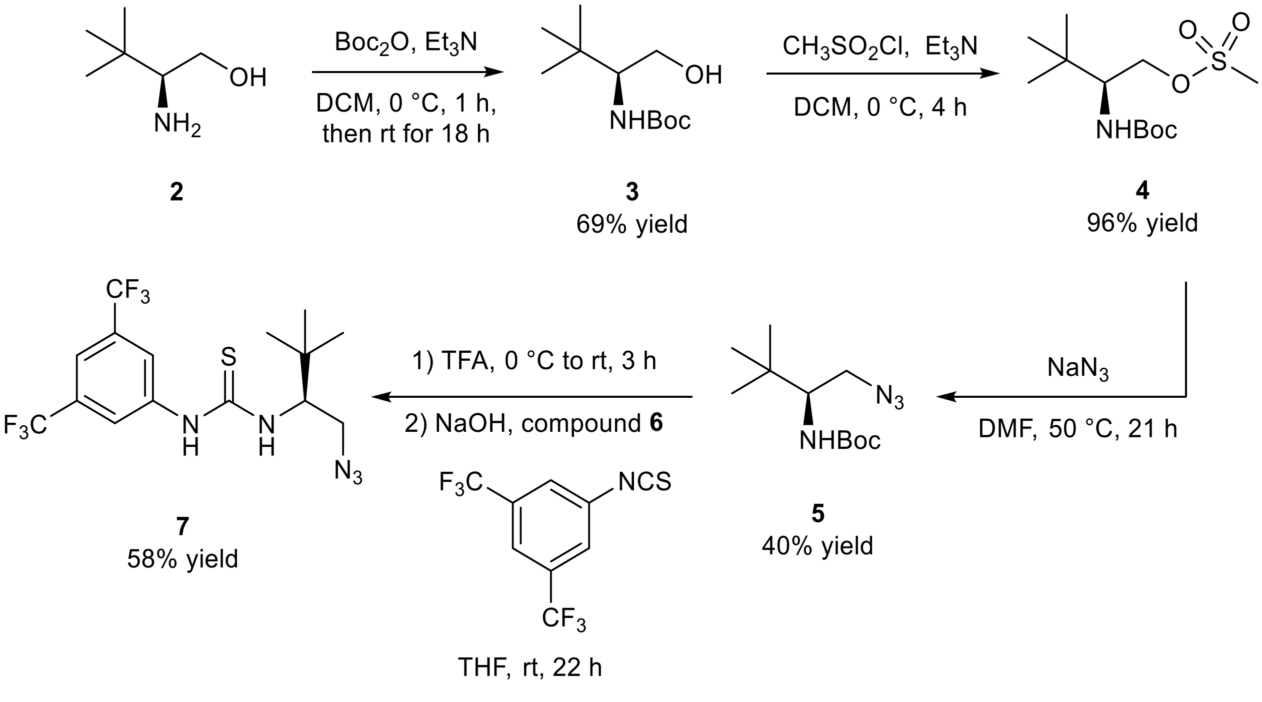

Synthesis of BIMP catalyst 1a

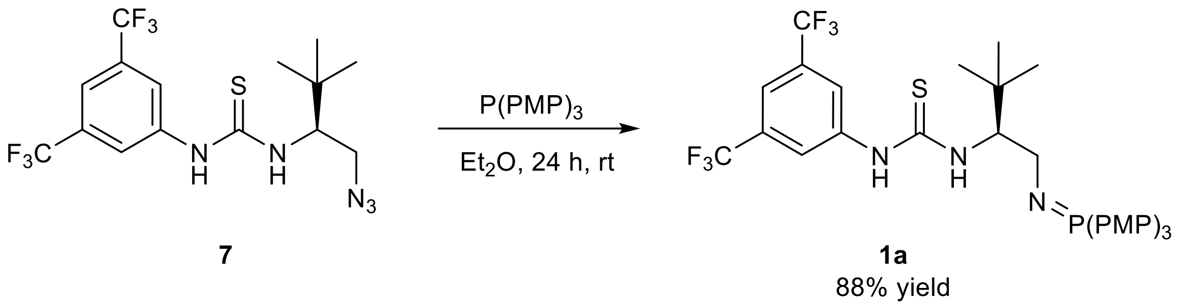

According to a literature procedure,[5] under nitrogen atmosphere, to 100 mg of azide **7** (0.24 mmol, 1 equiv.) in Et_2O (0.6 mL) was added 85 mg of tris(4-methoxyphenyl)phosphine $P(PMP)_3$ (0.24 mmol, 1 equiv.) at rt. The reaction mixture was stirred at rt for 24 h and concentrated under a stream of nitrogen. Pentane was added and the resultant thick precipitate was filtered. The precipitate was washed with pentane/Et_2O 1:1 and dried in vacuo to obtain 156 mg of catalyst **1a** (88% yield) as a white solid. All the analytical data are in agreement with the literature.[5]

Synthesis of BIMP catalyst 1b

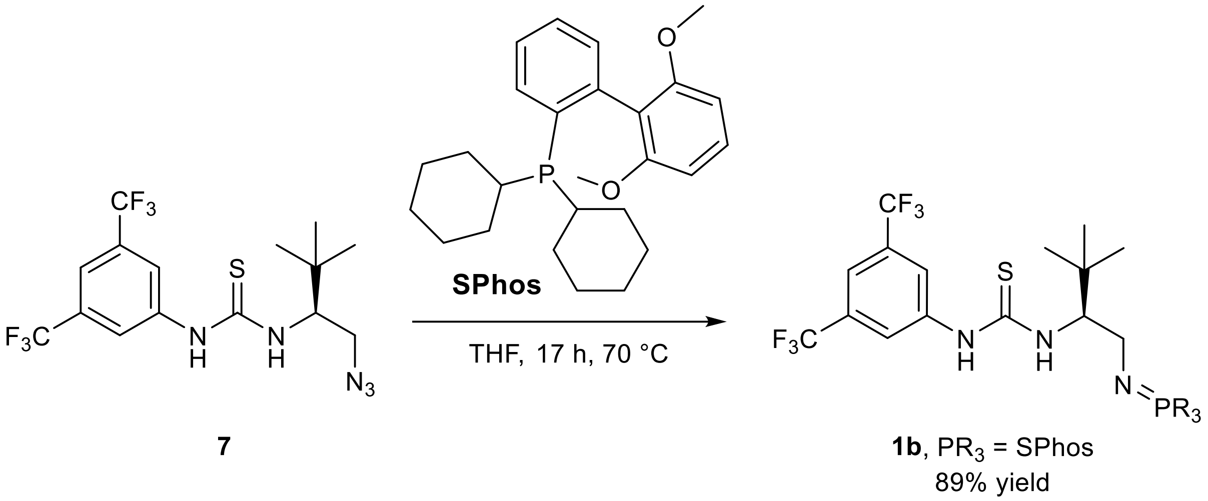

According to a literature procedure,[1] in a high-pressure vial (10 mL) were added 100 mg of azide **7** (0.24 mmol, 1 equiv.) and 98.5 mg of phosphine SPhos (0.24 mmol, 1.0 equiv.) 4 mL of dry tetrahydrofuran (THF). The reaction mixture was stirred at 70 °C for 17h. After the consumption of starting material (monitored by ^{31}P NMR), THF was removed under the flow of nitrogen and dried additionally on high-vacuum pump to obtain 170 mg of catalyst **1b** (89% yield) without further purification as a shiny light-yellow solid. All the analytical data are in agreement with the literature.[1]

Synthesis of BIMP catalyst 1c

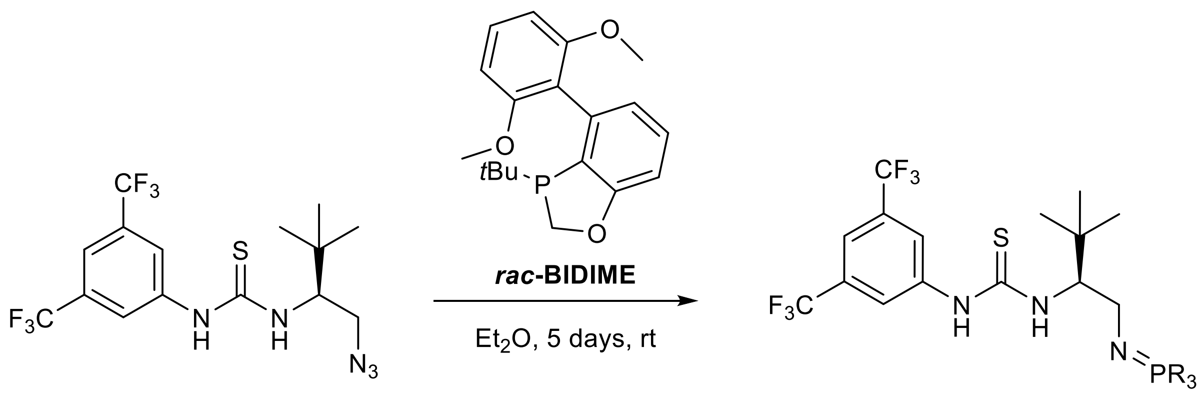

7

1c, PR_3 = *rac*-BIDIME
70% yield

According to a literature procedure,[1] in the two-necked flask was added 50 mg of azide **7** (0.12 mmol, 1 equiv.) and 39.6 mg of *rac*-BIDIME (0.12 mmol, 1 equiv.) in 2 mL of dry Et_2O. The reaction mixture was stirred at rt, until the completion of the reaction. The reaction was monitored by TLC, ^{1}H NMR, ^{19}F NMR and ^{31}P NMR. After 5 days stirring at rt, solvent was removed under the flow of nitrogen and catalyst **1c** was isolated as a pale-yellow solid (60 mg, ~70% pure catalyst by ^{31}P NMR, ~30% unreacted phosphine present). It was directly used, without further purification. All the analytical data are in agreement with the literature.[1]

Synthesis of BIMP catalyst precursors 11a and 11b

In contrast, squaramide-based BIMPs (catalysts **1d-e**) were not isolated but generated *in situ* from stable precursors **11a-b** during the asymmetric addition of malononitrile to trifluoromethyl aryl ketimines.

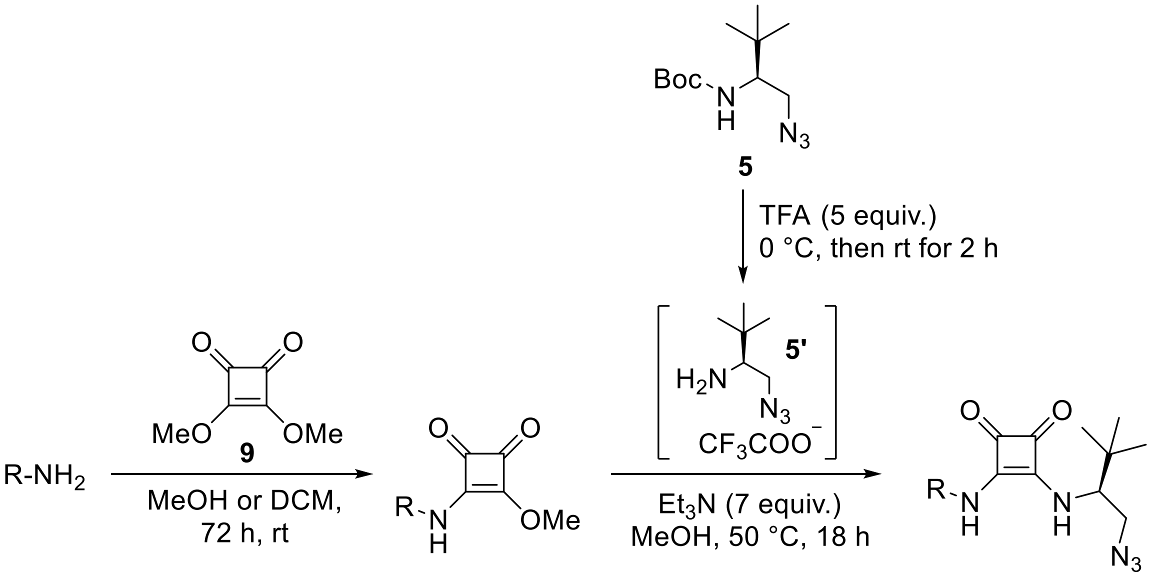

8a R= 3,5-$(CF_3)_2C_6H_3$
8b R= -CH_2(3,5-$(CF_3)_2C_6H_3$)

10a, 92% yield
10b, 55% yield

11a, 85% yield
11b, 68% yield

According to a literature procedure,[6] intermediates **10a** and **10b** were accessed by a condensation reaction of the corresponding amine intermediates **8a-b** with commercially available dimethyl squarate **9** under mild conditions. After a filtration on celite pad and a chromatographic purification, hemisquaramides **10a** and **10b** were obtained in 55 and 92% yield, respectively. All the analytical data are in agreement with the literature.[6]

At this point, 200 mf of azide **5** (0.82 mmol, 1.0 equiv.) were cooled to 0°C with an ice-bath. Under nitrogen atmosphere, 316 μL of TFA (4.13 mmol, 5.0 equiv.) were added dropwise. The reaction mixture was warmed to room temperature and, after stirring for 2 hours, the excess TFA was removed by flushing nitrogen. The proper hemisquaramide **10a** or **10b** (0.75 mmol, 0.91 equiv.) was added followed by anhydrous methanol (1.4 mL) and TEA (400 μL, 2.87 mmol, 7.0 equiv.). After reacting at 50°C for 18 h, the solvent was evaporated in vacuo and the crude product purified by flash column chromatography over silica (DCM, then DCM/MeOH from 98:2 to 95:5) to afford pre-catalysts **11a** and **11b** in high yields.

(S)-3-((1-azido-3,3-dimethylbutan-2-yl)amino)-4-((3,5-bis(trifluoromethyl)phenyl)amino)cyclobut-3-ene-1,2-dione (11a)

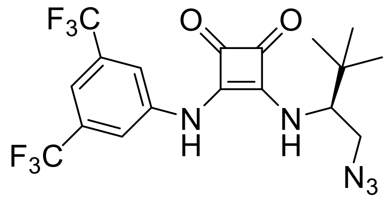

White solid (0.28 g, 85 % yield). **Rf =** 0.6 (*n*-Hex/AcOEt 3:7). **^{1}H NMR** (DMSO-d6, 300 MHz): δ = 10.20 (s, 1H), 8.07 (s, 2H), 7.86 (d, *J* = 10.3 Hz, 1H), 7.71 (s, 1H), 4.06 (t, *J* = 8.5 Hz, 0H), 3.81 (dd, *J* = 13.2, 3.1 Hz, 1H), 3.51 (dd, *J* = 13.1, 10.1 Hz, 1H), 0.97 (s, 7H). **^{13}C NMR** (DMSO-d6, 75 MHz): δ = 184.3, 180.7, 170.1, 162.5, 140.9, 131.3 (q, *J* = 32.9 Hz), 123.2 (q, *J* = 272.8 Hz), 118.2 (d, *J* = 4.1 Hz), 114.9, 62.6, 51.2, 34.2, 25.9. **^{19}F NMR** (DMSO-d6, 57 MHz): δ = -59.8. **HRMS (ESI$^+$):** m/z [M+Na] calcd for $C_{18}H_{17}N_5O_2F_6$: 472.1184; found: 472.1184. $[\alpha]_D^{30}$= +17.6 (DMSO, c = 0.92).

(S)-3-((1-azido-3,3-dimethylbutan-2-yl)amino)-4-((3,5 bis(trifluoromethyl)benzyl)amino)cyclobut-3-ene-1,2-dione (11b)

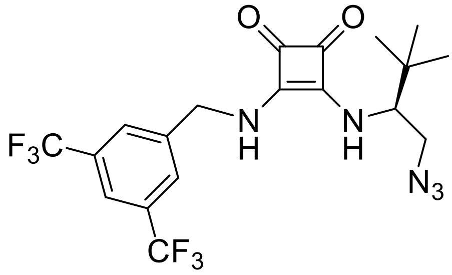

Yellow solid (0.26 g, 68% yield). **Rf =** 0.66 (DCM/MeOH 98:2). **^{1}H NMR** (DMSO-d6, 300 MHz): δ = 8.10 – 8.02 (m, 1H), 7.96 (bs, 1H), 7.48 (s, 1H), 7.05 (bs, 1H), 5.08 (t, *J* = 5.7 Hz, 1H), 4.19 (bs, 1H), 3.68 (dd, *J* = 12.9, 3.2 Hz, 1H), 3.46 (t, *J* = 11.0 Hz, 1H), 0.98 (s, 9H). **^{13}C NMR** (DMSO-d6, 75 MHz): δ = 182.8, 182.5, 168.6, 167.5, 142.7, 142.66, 131.0 (q, *J* = 32.9 Hz), 128.9 (q, *J* = 23.2 Hz), 126.9 (q, *J* = 197.6 Hz), 121.2, 62.3, 51.1, 45.7, 25.9. **^{19}F NMR** (DMSO-d6, 75 MHz): δ = -59.4. **HRMS (ESI$^+$):** *m/z* [M+Na] calcd for $C_{19}H_{19}N_5O_2F_6$: 486.1341; found: 486.1341. $[\alpha]_D^{30}$= -33.0 ($CHCl_3$, c = 0.91).

Synthesis of BIMP catalysts 1d and 1e

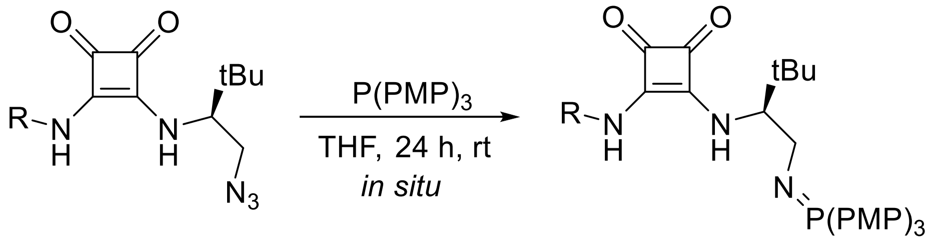

11a R= 3,5-$(CF_3)_2C_6H_3$ **1d** R= 3,5-$(CF_3)_2C_6H_3$
11b R= -CH_2(3,5-$(CF_3)_2C_6H_3$) **1e** R= -CH_2(3,5-$(CF_3)_2C_6H_3$)

Tris-(4-methoxyphenyl)phosphine (7.0 mg, 0.020 mmol, 1.0 equiv.) and the proper squaramide pre-catalyst **11a** or **11b** (0.020 mmol, 1.0 equiv.) were mixed together in dry THF (0.4 mL) under nitrogen atmosphere. The mixture was reacted until the complete consumption of the starting material. After about 24 h the solvent was removed under nitrogen stream, and the catalysts **1d-1e** were directly used in the enantioselective addition of malononitrile without any purification.

2.2 Synthesis of *N*-Boc aryl trifluoromethyl ketimines 12a-f

General procedure for the synthesis of *N*-Boc trifluoromethyl aryl ketimines 12a-f (GP1)

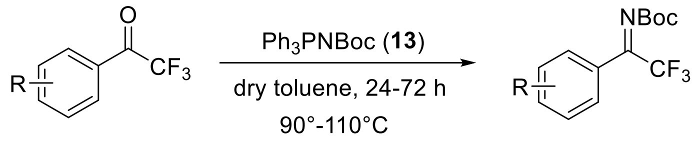

14a-f **12a-f**

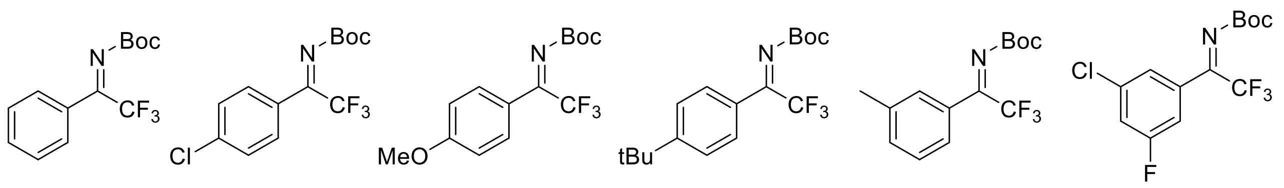

12a 24h, 81%yield

12b 24h, 52%yield

12c 72h, 53%yield

12d 72h, 47%yield

12e 16h, 50%yield

12f 24h, 62%yield

According to a literature procedure,[7] to a solution of 300 mg (1 equiv.) of the corresponding commercially available trifluoroacetophenone **14a-f** in dry toluene (5 mL) was added *N*-Boc-imino-(triphenyl)-phosphorane **13** (2 equiv). The reaction mixture was heated and stirred for 24-72 h (depending on starting ketone) at 90-110°C. The reaction was monitored by TLC and/or ^{1}H NMR in $CDCl_3$. After the consumption of starting ketone, the reaction was cooled down to room temperature and toluene was removed under reduced pressure. The residue was purified by silica gel column chromatography (eluent: n-hexane/ethyl acetate 98:2 to n-hexane/ethyl acetate 9:1) to afford ketoimines **12a-f** in from modest to good yields. All the analytical data are in agreement with the literature.[1,8]

2.3 Enantioselective synthesis of compound 16a

General procedure for the enantioselective addition of malononitrile scope promoted by BIMP cat. 1a-1c (GP2)

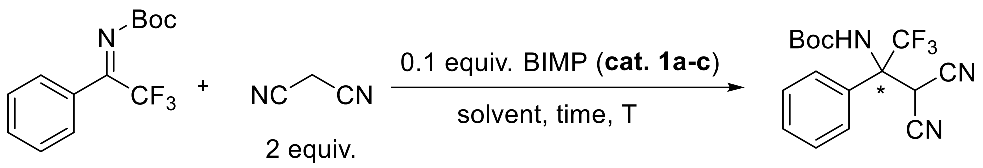

12a **15** **16a**

A 10 mL Schlenk tube under nitrogen was charged with 56.7 mg of ketimine **12a** (0.2 mmol, 1 equiv., 0.27 M in the appropriate solvent) and the appropriate iminophosphorane catalyst **cat. 1a-c** (0.02 mmol, 0.1 equiv.). The reaction mixture was cooled down at 0°C or -20°C, and 26.4 mg of malononitrile **15** (0.4 mmol, 2 equiv.) were added. The reaction was stirred until complete consumption of the starting material, then the solvent was removed under reduced pressure and the crude was purified by column chromatography on silica gel (n-Hexane/AcOEt from 100:0 to 90:10) to afford compound **16a**.

General procedure for the enantioselective addition of malononitrile scope promoted by BIMP cat. 1d-1e (GP3)

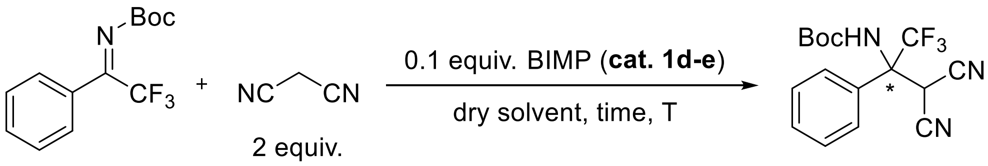

12a **15** **16a**

The iminophosphorane catalysts **cat. 1d** and **1e** (0.02 mmol, 0.1 equiv.) prepared in situ from pre-catalysts **11a** and **11b** respectively, were dissolved in dry solvent (0.800 mL) under nitrogen atmosphere. Then, 26.4 mg of malononitrile **15** (0.4 mmol, 2 equiv.) and 56.7 mg of ketimine **12a** (0.2 mmol, 1 equiv., 0.25 M in toluene) were added. The mixture was cooled down at 0°C or -20°C. The reaction was stirred until complete consumption of the starting material, then the solvent was evaporated in vacuo and the crude product purified by flash column chromatography over silica (n-Hexane/AcOEt from 100:0 to 90:10) to afford compound **16a**.

2.4 Enantioselective synthesis of compounds 16a-f

General procedure for the enantioselective addition of malononitrile scope promoted by BIMP cat. 1a (GP4)

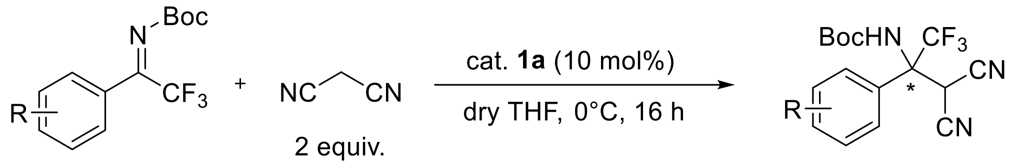

12a-f **15** **16a-f**

A 10 mL Schlenk tube under nitrogen was charged with ketimine **12a-f** (0.2 mmol, 1 equiv., 0.27 M in THF) and 14.6 mg of iminophosphorane catalyst **cat. 1a** (0.02 mmol, 0.1 equiv.). The reaction mixture was cooled down to 0°C, and 26.4 mg of malononitrile **15** (0.4 mmol, 2 equiv.) were added. The reaction mixture was stirred for 16h at 0°C, then THF was removed under reduced pressure and the crude was purified by column chromatography on silica gel (n-Hexane/AcOEt from 100:0 to 90:10) to afford compounds **16a-f**.

tert-butyl (3,3-dicyano-1,1,1-trifluoro-2-phenylpropan-2-yl)carbamate (16a)

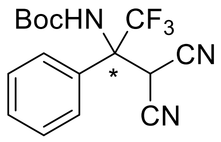

Starting from ketimine **12a** (54.65 mg), tert-butyl (3,3-dicyano-1,1,1-trifluoro-2-phenylpropan-2-yl)carbamate **16a** was prepared according to **GP2-4**. After purification by flash column chromatography, it was obtained in 68% yield from **GP4** (46.2 mg) as a white solid.

^{1}**H NMR** (300 MHz, $CDCl_3$) δ 7.79 – 7.65 (m, 2H), 7.62 – 7.45 (m, 3H), 5.85 (s, 1H), 5.43 (s, 1H), 1.48 (s, 9H). ^{19}**F NMR** (282 MHz, $CDCl_3$) δ -71.7. ^{13}**C NMR** (101 MHz, $CDCl_3$) δ 153.7, 131.8, 131.0, 129.6, 126.57 (2C), 126.6, 124.1 (q, J = 288.8 Hz), 110.3, 109.9, 83.4, 65.3 (q, J = 28.3 Hz), 29.6, 28.18 (3C). **Rf =** 0.28 (n-hexane/ethyl acetate 8:2).

HRMS (ESI$^+$): [M+Na] *m/z* calcd for $C_{16}H_{16}N_3O_4F_3$: 362.1092; found: 362.1085.

tert-butyl (2-(4-chlorophenyl)-3,3-dicyano-1,1,1-trifluoropropan-2-yl)carbamate (16b)

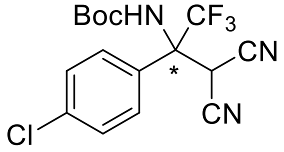

Starting from ketimine **12b** (61.54 mg), tert-butyl (2-(4-chlorophenyl)-3,3-dicyano-1,1,1-trifluoropropan-2-yl)carbamate **16b** was prepared according to **GP4**. After purification by flash column chromatography, it was obtained in 73% yield (54.6 mg) as a white solid.

^{1}**H NMR** (300 MHz, $CDCl_3$) δ 7.66 – 7.60 (m, 2H), 7.53 – 7.45 (m, 2H), 5.79 (s, 1H), 5.49 (s, 1H), 1.48 (s, 9H). ^{19}**F NMR** (282 MHz, $CDCl_3$) δ -72.1. ^{13}**C NMR** (75 MHz, $CDCl_3$) δ 153.6, 137.5, 130.2, 129.88 (2C), 128.12 (2C), 123.9 (q, J = 288.6 Hz), 110.0, 109.8, 83.7, 65.0 (q, J = 28.6 Hz), 29.6, 28.17 (3C). **Rf =** 0.50 (n-hexane/ethyl acetate 8:2).

HRMS (ESI$^+$): [M+Na] *m/z* calcd for $C_{16}H_{15}N3O_2F_3Cl$: 396.0703; found: 396.0698.

tert-butyl (3,3-dicyano-1,1,1-trifluoro-2-(4-methoxyphenyl)propan-2-yl)carbamate (16c)

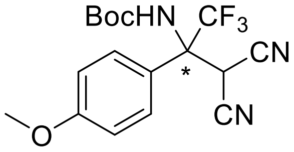

Starting from ketimine **12c** (60.66 mg), tert-butyl (3,3-dicyano-1,1,1-trifluoro-2-(4-methoxyphenyl)propan-2-yl)carbamate **16c** was prepared according to **GP4**. After purification by flash column chromatography, it was obtained in 90% yield (66.5 mg) as a sticky solid.

^{1}H NMR (300 MHz, $CDCl_3$) δ 7.62 (d, *J* = 8.8 Hz, 2H), 7.07 – 6.95 (m, 2H), 5.82 (s, 1H), 5.42 (s, 1H), 3.85 (s, 3H), 1.48 (s, 9H). **^{19}F NMR** (282 MHz, $CDCl_3$) δ -71.9. **^{13}C NMR** (75 MHz, $CDCl_3$) δ 161.3, 153.7, 124.3 (q, *J* = 288.8 Hz), 128.07 (2C), 123.4, 114.92 (2C), 110.4, 110.0, 83.2, 65.0 (q, *J* = 28.6 Hz), 55.6, 29.8, 28.20 (3C). **Rf** = 0.30 (hexane: ethyl acetate = 8:2).

HRMS (ESI$^+$): [M+Na] *m/z* calcd for $C_{17}H_{18}N_3O_3F_3$: 392.1198; found: 392.1193.

tert-butyl (2-(4-(tert-butyl)phenyl)-3,3-dicyano-1,1,1-trifluoropropan-2-yl)carbamate (16d)

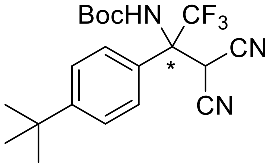

Starting from ketimine **12d** (65.87 mg), tert-butyl (2-(4-(tert-butyl)phenyl)-3,3-dicyano-1,1,1-trifluoropropan-2-yl)carbamate **16d** was prepared according to **GP4**. After purification by flash column chromatography, it was obtained in 60% yield (47.5 mg) as a white solid.

^{1}H NMR (300 MHz, $CDCl_3$) δ 7.62 (d, *J* = 8.6 Hz, 2H), 7.54 – 7.52 (m, 1H), 7.52 – 7.48 (m, 1H), 5.88 (s, 1H), 5.45 (s, 1H), 1.48 (s, 9H), 1.34 (s, 9H). **^{19}F NMR** (282 MHz, $CDCl_3$) δ -71.6. **^{13}C NMR** (75 MHz, $CDCl_3$) δ 154.2, 130.3, 128.6, 126.57 (2C), 126.28 (2C), 124.4 (q, *J* = 288.8 Hz), 110.4, 110.0, 83.1, 65.1 (q, *J* = 28.5 Hz), 34.9, 31.17 (3C), 29.4, 28.16 (3C). **Rf** = 0.56 (hexane: ethyl acetate = 8:2).

HRMS (ESI$^+$): [M+Na] *m/z* calcd for $C_{20}H_{24}N_3O_2F_3$: 418.1718; found: 418.1710.

tert-butyl (3,3-dicyano-1,1,1-trifluoro-2-(m-tolyl)propan-2-yl)carbamate (16e)

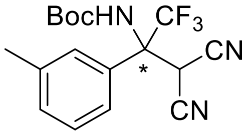

Starting from ketimine **12e** (57.46 mg), tert-butyl (3,3-dicyano-1,1,1-trifluoro-2-(m-tolyl)propan-2-yl)carbamate **16e** was prepared according to **GP4**. After purification by flash column chromatography, it was obtained in 74% yield (52.3 mg) as a white solid.

^{1}H NMR (300 MHz, $CDCl_3$) δ 7.49 (d, *J* = 8.0 Hz, 2H), 7.45 – 7.30 (m, 2H), 5.84 (s, 1H), 5.49 (s, 1H), 2.44 (d, *J* = 1.5 Hz, 3H), 1.49 (s, 9H). **^{19}F NMR** (282 MHz, $CDCl_3$) δ -71.6. **^{13}C NMR** (75 MHz, $CDCl_3$) δ 153.7, 139.6, 131.73 (2C), 129.5, 127.0, 124.1 (q, J = 288.8 Hz), 123.6, 110.3, 110.0, 83.3, 65.2 (q, J = 28.5 Hz), 29.5, 28.17 (3C), 21.8. **Rf** = 0.39 (hexane: ethyl acetate = 8:2).

HRMS (ESI$^+$): [M+Na] *m/z* calcd for $C_{17}H_{18}N_3O_2F_3$: 376.1249; found: 376.1246.

tert-butyl (2-(3-chloro-5-fluorophenyl)-3,3-dicyano-1,1,1-trifluoropropan-2-yl)carbamate (16f)

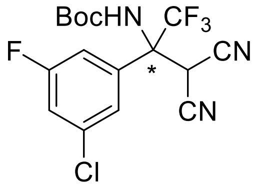

Starting from ketimine **12f** (65.14 mg), tert-butyl (2-(3-chloro-5-fluorophenyl)-3,3-dicyano-1,1,1-trifluoropropan-2-yl)carbamate **16f** was prepared according to **GP4**. After purification by flash column chromatography, it was obtained in 78% yield (61.1 mg) as a white solid.

^{1}H NMR (400 MHz, $CDCl_3$) δ 7.47 (s, 1H), 7.32 (dt, *J* = 9.4, 2.1 Hz, 1H), 7.28 (dt, *J* = 7.8, 2.0 Hz, 1H), 5.74 (s, 1H), 5.59 (s, 1H), 1.48 (s, 9H). **^{19}F NMR** (282 MHz, $CDCl_3$) δ -72.2, -107.0. **^{13}C NMR** (101 MHz, $CDCl_3$) δ 162.7 (d, *J* = 252.6 Hz), 153.3, 136.7 (d, *J* = 10.6 Hz), 135.1 (d, *J* = 8.2 Hz), 123.4 (q, *J* = 288.5 Hz), 123.0 (dd, *J* = 3.5, 1.8 Hz), 119.0 (d, *J* = 24.3 Hz), 113.0 (dd, *J* = 25.1, 1.9 Hz), 109.6, 109.3, 84.0, 64.7 (ddd, *J* = 56.3, 27.6, 1.4 Hz), 31.1, 28.00 (3C). **Rf** = 0.45 (hexane: ethyl acetate = 8:2).

HRMS (ESI^+): [M+Na] *m/z* calcd for $C_{16}H_{14}N_3O_2F_4Cl$: 414.0608; found: 414.0611.

2.5 Racemic synthesis of compounds 16a-f

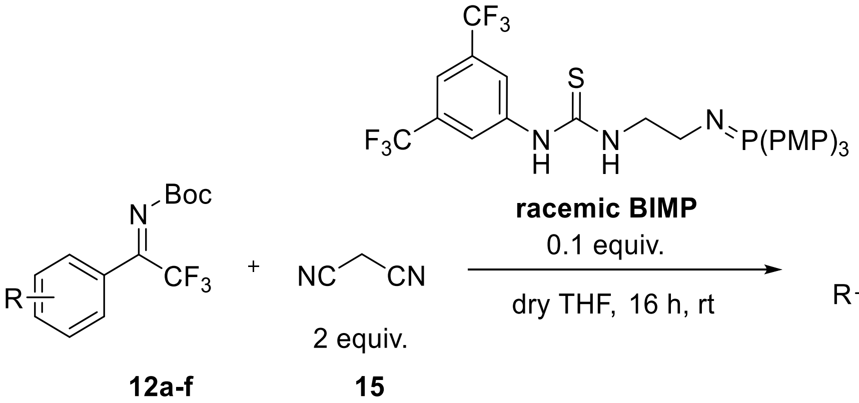

Under nitrogen atmosphere, a Schlenk tube was charged with ketimine **12a-f** (0.2 mmol, 1 equiv., 0.27 M in THF) and 14.6 mg of racemic BIMP (0.02 mmol, 0.1 equiv.). To the reaction mixture 26.4 mg of malononitrile **15** (0.4 mmol, 2 equiv.) were added. The reaction mixture was stirred at rt for 16h, then THF was removed under reduced pressure and the crude was purified by silica gel column chromatography (n-Hexane/AcOEt from 100:0 to 90:10) to afford compounds **16a-f**.

2.6 Synthesis of quaternary α-amino esters (17af)

General procedure for oxidative decyanation of 16a-f (GP5)

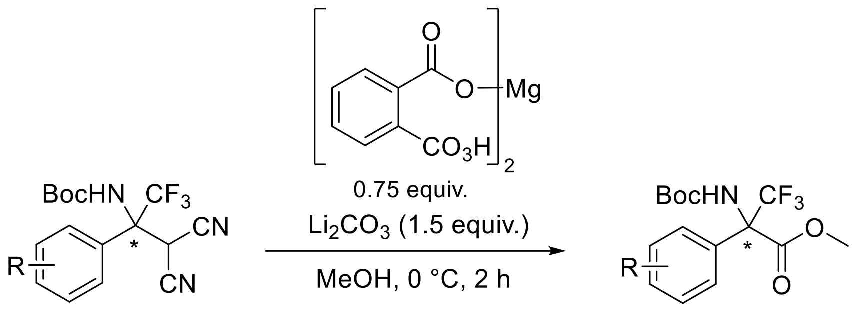

16a-f **17a-f**

Under nitrogen atmosphere, a flask was charged with compounds **16a-f** (1 equiv., 0.1 M in dry methanol) and dry methanol at 0°C. To the stirring solution, was added magnesium monoperoxyphthalate hexahydrate (0.75 equiv.), Li_2CO_3 (1.5 equiv.). The reaction mixture was stirred for 2 h at 0 °C. The reaction was stopped and quenched by water and extracted with DCM (3 x 5 mL). The combined organic layers were dried over anhydrous Na_2SO_4, filtered and concentrated under reduced pressure to afford quaternary amino esters **17a-f**. Determination of enantiomeric excesses was conducted by chiral HPLC (column: Chiralpak AD, Lux Phenomenex 3µm Amylose-1; eluent: *n*-hexane/isopropanol 95:5, flow rate 1 mL/min).

methyl 2-((tert-butoxycarbonyl)amino)-3,3,3-trifluoro-2-phenylpropanoate (17a)

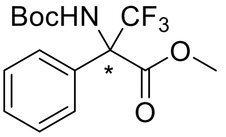

Starting from **16a** (46.2 mg, 0.136 mmol), methyl 2-((tert-butoxycarbonyl)amino)-3,3,3-trifluoro-2-phenylpropanoate **17a** was prepared according to **GP5**. It was obtained in 93% yield and 74% enantiomeric excess (42.2 mg) as a white solid.

^{1}H NMR (300 MHz, $CDCl_3$) δ 7.52 – 7.37 (m, 5H), 5.62 (s, 1H), 3.83 (s, 3H), 1.41 (s, 9H). **^{19}F NMR** (282 MHz, $CDCl_3$) δ -71.7. **^{13}C NMR** (75 MHz, $CDCl_3$) δ 166.9, 153.7, 132.9, 129.6, 128.97 (2C), 126.74 (2C), 123.8 (q, *J* = 286.9 Hz), 81.6, 67.8 (d, *J* = 27.6 Hz), 53.5, 28.18 (3C). **HPLC conditions**: Lux Phenomenex 3µm Amylose-1 Hex_IPA_ 95_5 1 mL/min, t_{major} 10.17 min, t_{minor} 7.11 min, 74% *e.e.*.

HRMS (ESI$^+$): [M+Na] *m/z* calcd for $C_{15}H_{18}NO_4F_3$: 356.1086; found: 356.1087.

methyl 2-((tert-butoxycarbonyl)amino)-2-(4-chlorophenyl)-3,3,3-trifluoropropanoate (17b)

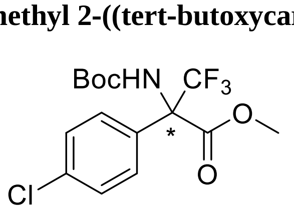

Starting from **16b** (54.6 mg, 0.146 mmol), methyl 2-((tert-butoxycarbonyl)amino)-2-(4-chlorophenyl)-3,3,3-trifluoropropanoate **17b** was prepared according to **GP5**. It was obtained in 88% yield and 63% enantiomeric excess (47.3 mg) as a colourless oil.

^{1}H NMR (300 MHz, $CDCl_3$) δ 7.47 – 7.41 (m, 2H), 7.37 (dq, *J* = 9.0, 2.4 Hz, 2H), 5.72 (s, 1H), 3.82 (s, 3H), 1.39 (s, 10H). **^{19}F NMR** (282 MHz, $CDCl_3$) δ -71.61. **^{13}C NMR** (75 MHz, CDCl3) δ 166.5, 153.6, 135.6, 131.3, 129.01 (2C), 128.45 (2C), 123.6 (q, J = 287.3 Hz), 81.8, 67.5 (q, J = 28.7 Hz), 53.8, 28.13 (3C). **HPLC conditions**: Chiralpak AD Hex_IPA_ 95_5 1 mL/min, t_{major} 10.02 min, t_{minor} 6.32 min, 63% *e.e.*.

HRMS (ESI$^+$): [M+Na] *m/z* calcd for $C_{15}H_{17}NO_4F_3Cl$: 390.0696; found: 390.0712.

methyl 2-((tert-butoxycarbonyl)amino)-3,3,3-trifluoro-2-(4-methoxyphenyl)propanoate (17c)

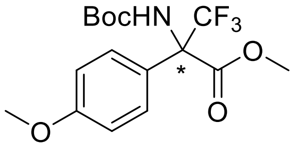

Starting from **16c** (66.5 mg, 0.180 mmol), methyl 2-((tert-butoxycarbonyl)amino)-3,3,3-trifluoro-2-(4-methoxyphenyl)propanoate **17c** was prepared according to **GP5**. It was obtained in 78% yield and 60% enantiomeric excess (51.0 mg) as a white solid.

^{1}H NMR (300 MHz, $CDCl_3$) δ 7.36 (d, *J* = 8.0 Hz, 2H), 6.91 (d, *J* = 6.9 Hz, 2H), 5.59 (s, 1H), 3.81 (s, 6H), 1.42 (s, 9H). **^{19}F NMR** (282 MHz, $CDCl_3$) δ -72.04. **^{13}C NMR** (75 MHz, $CDCl_3$) δ 167.0, 160.4, 153.7, 128.08 (2C), 124.7, 123.9 (q, *J* = 287.1 Hz), 114.3 (2C), 81.5, 67.3 (q, *J* = 28.3 Hz), 55.5, 53.4, 28.17 (3C). **HPLC conditions**: Chiralpak AD Hex_IPA_ 95_5 1 mL/min, t_{major} 18.30 min, t_{minor} 10.50 min, 60% *e.e.*.

HRMS (ESI$^+$): [M+Na] *m/z* calcd for $C_{16}H_{20}NO_5F_3$: 386.1191; found: 386.1192.

methyl 2-((tert-butoxycarbonyl)amino)-2-(4-(tert-butyl)phenyl)-3,3,3-trifluoropropanoate (17d)

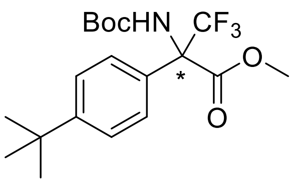

Starting from **16d** (47.5 mg, 0.120 mmol), methyl 2-((tert-butoxycarbonyl)amino)-2-(4-(tert-butyl)phenyl)-3,3,3-trifluoropropanoate **17d** was prepared according to **GP5**. It was obtained in 93% yield and 75% enantiomeric excess (43.5 mg) as a white solid.

^{1}H NMR (300 MHz, $CDCl_3$) δ 7.46 – 7.38 (m, 2H), 7.38 – 7.30 (m, 2H), 5.60 (s, 1H), 3.83 (s, 3H), 1.42 (s, 9H), 1.31 (s, 9H). **^{19}F NMR** (282 MHz, $CDCl_3$) δ -71.95. **^{13}C NMR** (101 MHz, $CDCl_3$) δ 167.0, 153.8, 152.8, 126.37 (2C), 125.97 (3C), 123.9 (d, *J* = 287.0 Hz), 81.5, 67.5 (d, *J* = 27.6 Hz), 53.3, 34.8, 31.27 (3C), 28.16 (3C). **HPLC conditions**: Lux Phenomenex 3μm Amylose-1 Hex_IPA_ 95_5 1 mL/min, t_{major} 6.38 min, t_{minor} 4.95 min, 75% *e.e.*.

HRMS (ESI$^+$): [M+Na] *m/z* calcd for $C_{19}H_{26}NO_4F_3$: 412.1712; found: 412.1707.

methyl 2-((tert-butoxycarbonyl)amino)-3,3,3-trifluoro-2-(m-tolyl)propanoate (17e)

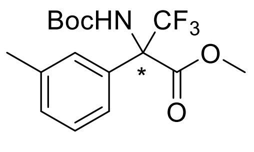

Starting from **16e** (53.2 mg, 0.150 mmol), methyl 2-((tert-butoxycarbonyl)amino)-3,3,3-trifluoro-2-(m-tolyl)propanoate **17e** was prepared according to **GP5**. It was obtained in 94% yield and 66% enantiomeric excess (49.2 mg) as a white solid.

^{1}H NMR (300 MHz, $CDCl_3$) δ 7.32 – 7.17 (m, 4H), 5.61 (s, 1H), 3.79 (s, 3H), 2.34 (s, 3H), 1.38 (s, 9H). **^{19}F NMR** (282 MHz, $CDCl_3$) δ -71.79. **^{13}C NMR** (75 MHz, $CDCl_3$) δ 166.9, 153.7, 138.8, 132.7, 130.4, 128.9, 127.2, 123.7, 123.7 (d, *J* = 287.3 Hz), 81.5, 67.5 (d, *J* = 28.2 Hz), 53.4, 28.16 (3C), 21.7. **HPLC conditions**: Lux Phenomenex 3μm Amylose-1 Hex_IPA_ 95_5 1 mL/min, t_{major} 6.85 min, t_{minor} 5.86 min, 66% *e.e.*.

HRMS (ESI$^+$): [M+Na] *m/z* calcd for $C_{16}H_{20}NO_4F_3$: 370.1242; found: 370.1234.

methyl 2-((tert-butoxycarbonyl)amino)-2-(3-chloro-5-fluorophenyl)-3,3,3-trifluoropropanoate (17f)

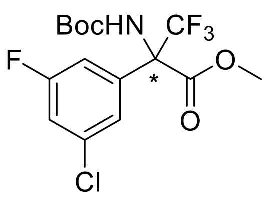

Starting from **16f** (61.1 mg, 0.156 mmol), methyl 2- ((tert-butoxycarbonyl)amino)-2-(3-chloro-5-fluorophenyl)-3,3,3-trifluoropropanoate **17f** was prepared according

to **GP5**. It was obtained in 93% yield and 38% enantiomeric excess (56.0 mg) as a white solid.

[1]**H NMR** (300 MHz, $CDCl_3$) δ 7.36 (s, 1H), 7.22 (dt, *J* = 9.8, 2.1 Hz, 1H), 7.13 (dt, *J* = 8.0, 2.1 Hz, 1H), 5.78 (s, 1H), 3.84 (s, 3H), 1.39 (s, 9H). [19]**F NMR** (282 MHz, $CDCl_3$) δ -71.35, -109.56. [13]**C NMR** (75 MHz, $CDCl_3$) δ 165.7, 162.4 (d, *J* = 250.0 Hz), 153.3, 136.1 (d, *J* = 8.5 Hz), 135.4 (d, *J* = 10.6 Hz), 123.4, 123.1 (q, *J* = 287.5 Hz), 117.2 (d, *J* = 24.6 Hz), 113.4 (d, *J* = 24.6 Hz), 82.0, 67.3 (q, *J* = 28.8 Hz), 54.1, 27.97 (3C). **HPLC conditions**: Lux Phenomenex 3µm Amylose-1 Hex_IPA_ 95_5 1 mL/min, t_{major} 4.98 min, t_{minor} 4.50 min, 38% *e.e.*.

HRMS (ESI$^+$): [M+Na] *m/z* calcd for $C_{15}H_{16}NO_4F_4Cl$: 408.0602; found: 408.0592.

2.7 Assignment of the absolute configuration of compound 16a

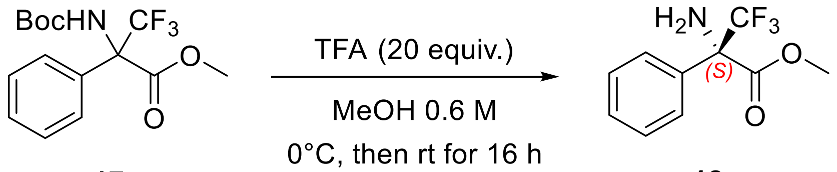

17a **18**

A flask was charged with 40 mg of compound **17a** (0.12 mmol, 1 equiv., 0.6 M in methanol) and 200 µL of methanol (2.4 mmol, 20 equiv.). The solution was cooled down to 0°C and 200µL of trifluoroacetic acid (TFA, 2.4 mmol, 20 equiv.) were added. The reaction mixture was allowed to warm to rt and stirred for 16 h. TFA was evaporated under nitrogen flux and a solution of 302 mg of sodium bicarbonate (3.6 mmol, 30 equiv.) in isopropanol (0.5 mL) was added to the resulting crude. After a filtration, the filtrated was concentrated under reduced pressure to give compound **18** as a colourless oil in 90% yield and 74% enantiomeric excess (25.2 mg). All the analytical data are in agreement with the literature.[10]

[1]**H NMR** (300 MHz, DMSO) δ 7.60 – 7.50 (m, 2H), 7.49 – 7.40 (m, 3H), 3.68 (s, 3H), 3.17 (s, 2H). [19]**F NMR** (282 MHz, DMSO) δ -73.53. (+)-(*S*)-Amino ester **18**. $[\alpha]_D^{18}$ = 2.58 (*c* 0.01 g/mL, MeOH).

2.8 Enantioselective synthesis of compound 20

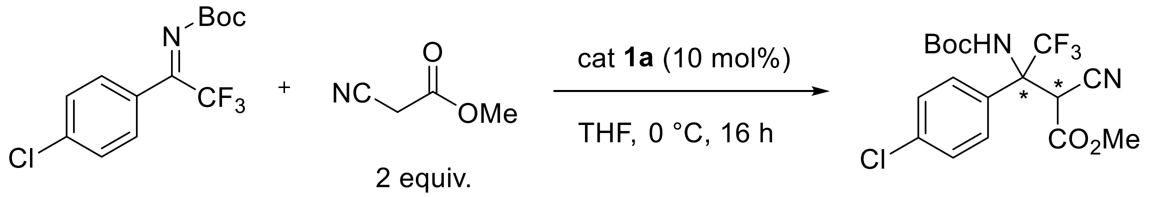

12b **19** **20**

55% yield, 50:50 *dr*

Under nitrogen atmosphere, a Schlenk tube was charged with 74.75 mg of ketimine **12b** (0.2 mmol, 1 equiv., 0.27 M in THF) and 14.6 mg of iminophosphorane **cat. 1a** (0.02 mmol, 0.1 equiv.). The reaction mixture was cooled down to 0°C, and 28µL of methyl 2-cyanoacetate **19** (0.4 mmol, 2 equiv.) were added. The reaction

mixture was stirred for 16h at 0°C. Then, THF was removed under reduced pressure and the crude was purified by silica gel column chromatography (n-Hexane/AcOEt from 100:0 to 90:10) to afford compound **20** in 55% yield as a colourless oil as a mixture of diastereomers 50:50 (44.7 mg).

^{1}H NMR (400 MHz, $CDCl_3$) δ 7.61 (d, J = 8.8 Hz, 2H), 7.52 (d, J = 8.5 Hz, 2H), 7.47 – 7.37 (m, 4H), 5.92 (s, 1H), 5.86 (s, 1H), 4.78 (s, 1H), 4.61 (s, 1H), 3.85 (s, 3H), 3.77 (s, 3H), 1.42 (s, 18H). **^{19}F NMR** (282 MHz, $CDCl_3$) δ -69.07. **^{13}C NMR** (101 MHz, $CDCl_3$) δ 163.5, 163.2, 153.4, 153.0, 136.3, 136.1, 131.5, 131.1, 129.16 (2C), 129.13 (2C), 128.4, 128.4, 128.2, 128.2, 124.4 (dq, J = 289.6, 6.3 Hz, 2C), 113.0, 112.8, 82.14 (2C), 65.4 (d, J = 28.7 Hz, 2C), 54.5, 54.4, 43.7, 42.4, 28.14 (6C). **Rf** = 0.19 (hexane: ethyl acetate = 8:2).

HRMS (ESI^{+}): [M+Na] *m/z* calcd for $C_{17}H_{18}N_3O_4F_3Cl$: 429.0805; found: 429.0804.

2.9 Racemic synthesis of compound 20

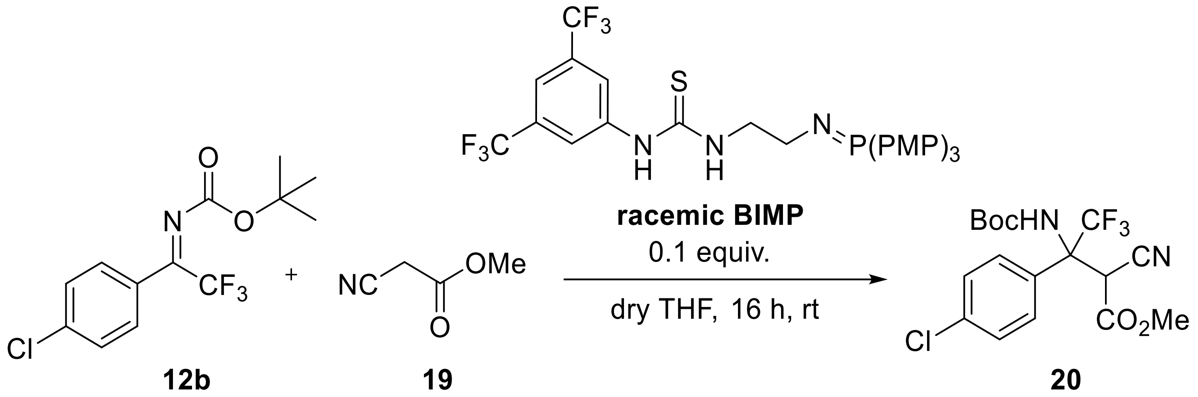

Under nitrogen atmosphere, a Schlenk tube was charged with 74.75 mg of ketimine **12b** (0.2 mmol, 1 equiv., 0.27 M in toluene) and 14.6 mg of racemic BIMP (0.02 mmol, 0.1 equiv.). To the reaction mixture 28µL of methyl 2-cyanoacetate **19** (0.4 mmol, 2 equiv.) were added. The reaction mixture was stirred at rt for 16 h. Then, THF was removed under reduced pressure and the crude was purified by column chromatography on silica gel (n-Hexane/AcOEt from 100:0 to 90:10) to afford compound **20**.

3. NMR spectra

(S)-3-((1-azido-3,3-dimethylbutan-2-yl)amino)-4-((3,5-bis(trifluoromethyl)phenyl)amino)cyclobut-3-ene-1,2-dione (11a)

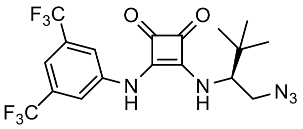

^{1}H NMR (300 MHz, DMSO-d_6)

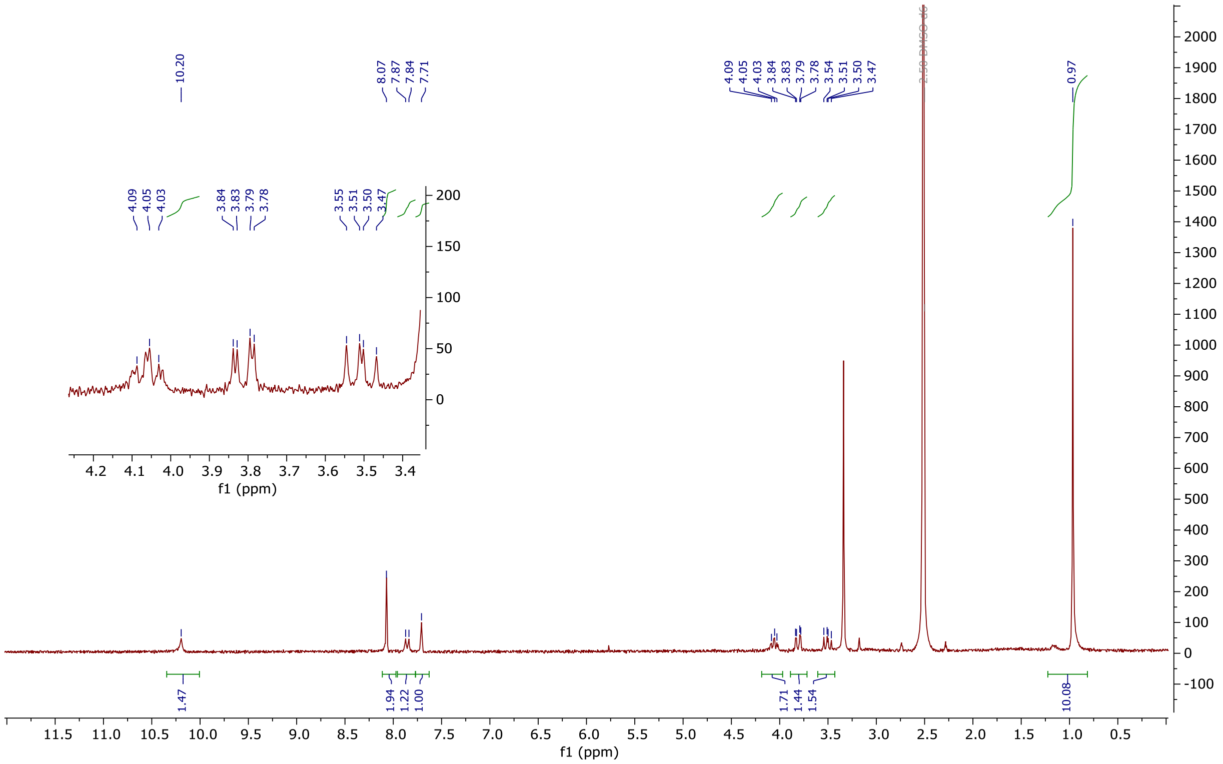

^{19}F NMR (57 MHz, DMSO-d_6)

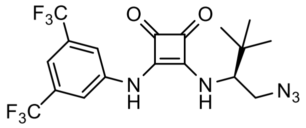

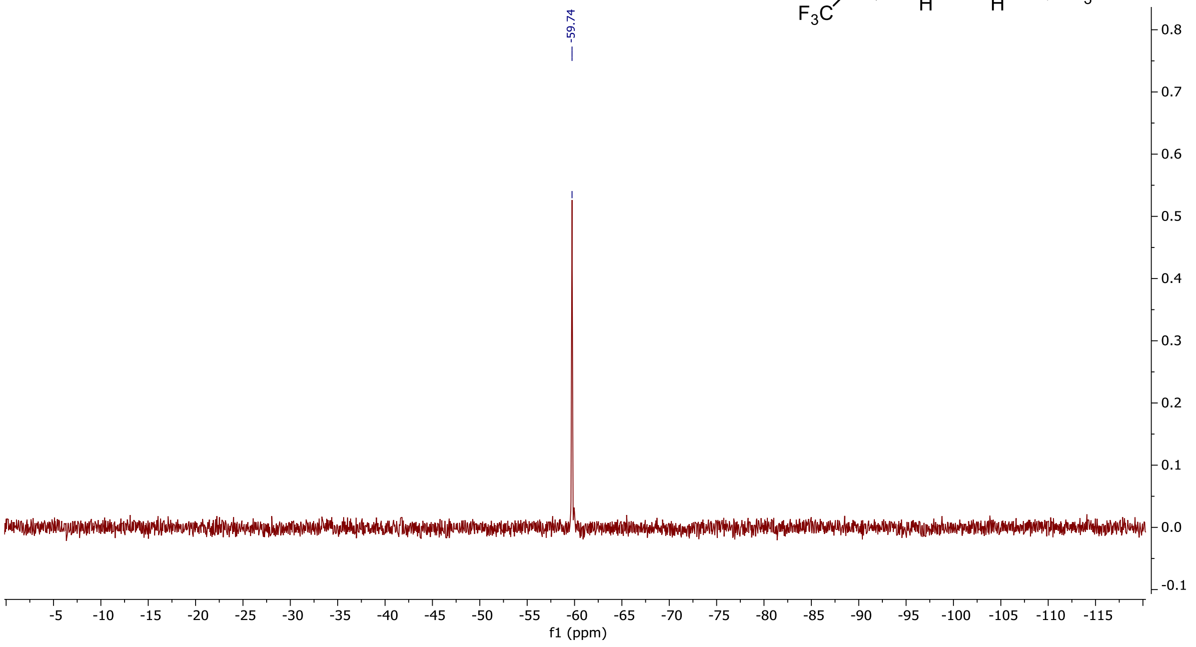

COSY (DMSO-d_6)

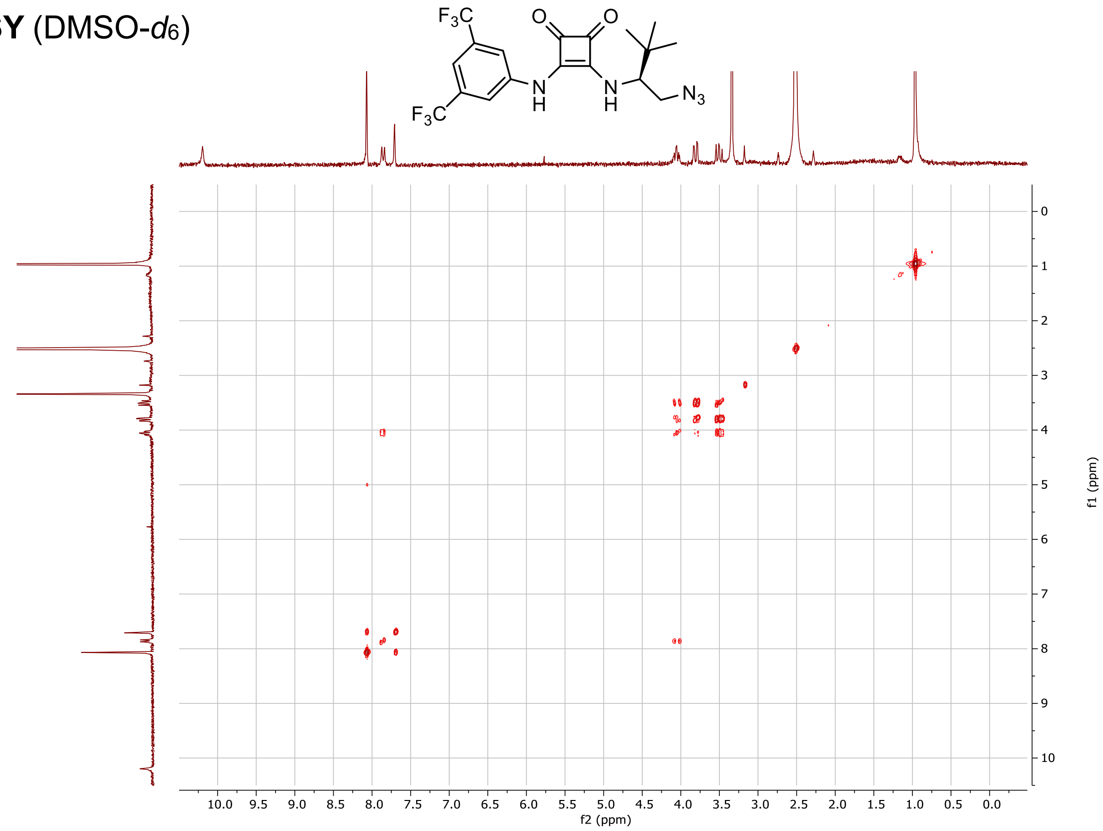

^{13}C NMR (75 MHz, DMSO-d_6)

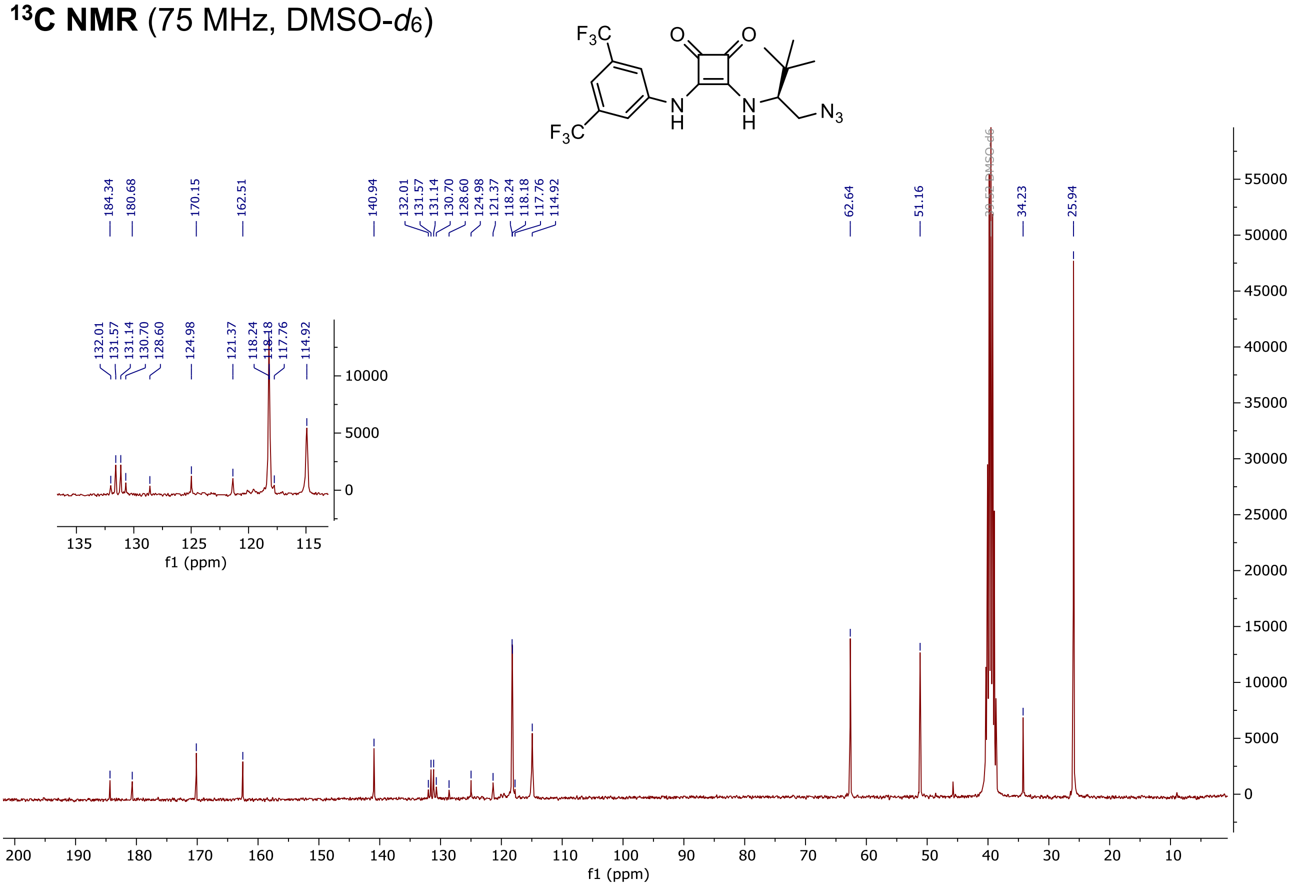

(S)-3-((1-azido-3,3-dimethylbutan-2-yl)amino)-4-((3,5 bis(trifluoromethyl)benzyl)amino)cyclobut-3-ene-1,2-dione (11b)

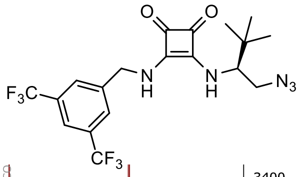

^{1}H NMR (300 MHz, DMSO-d_6)

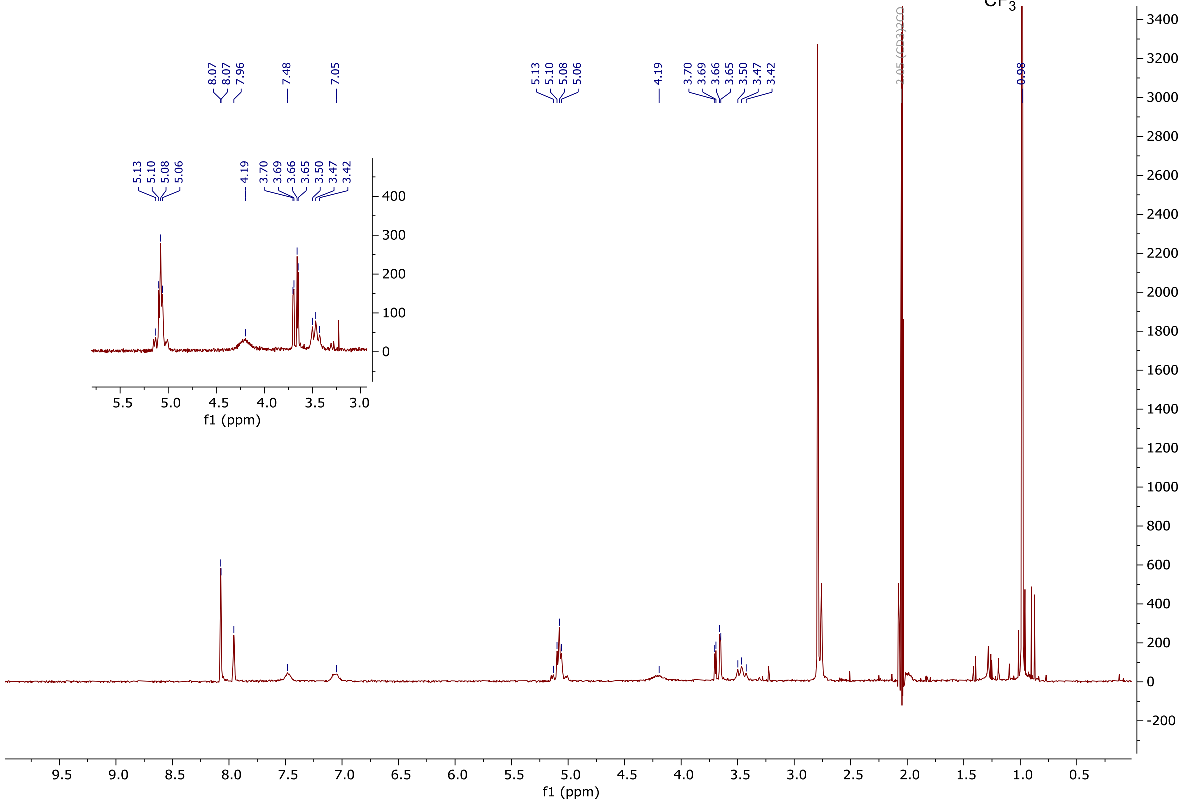

^{19}F NMR (57 MHz, DMSO-d_6)

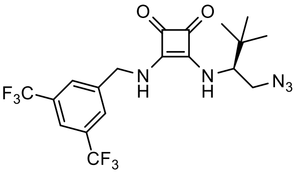

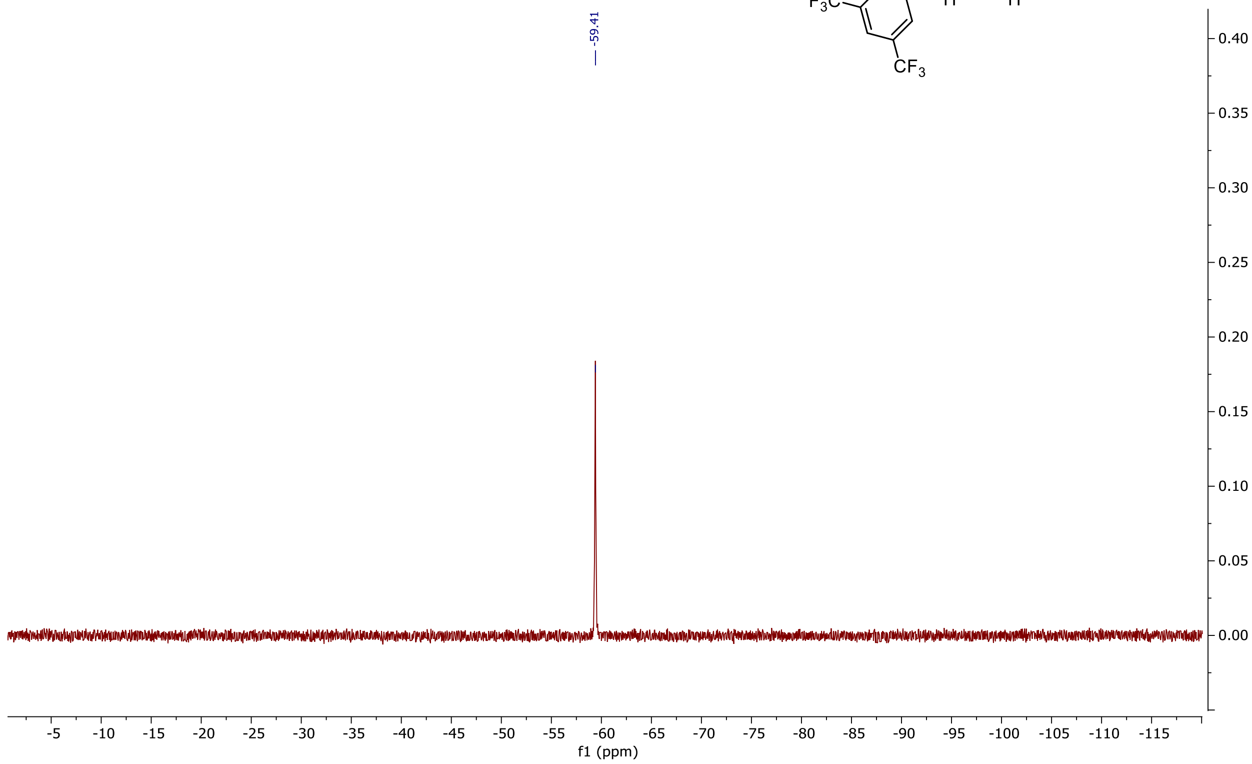

^{13}C NMR (75 MHz, DMSO-*d*6)

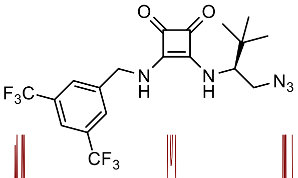

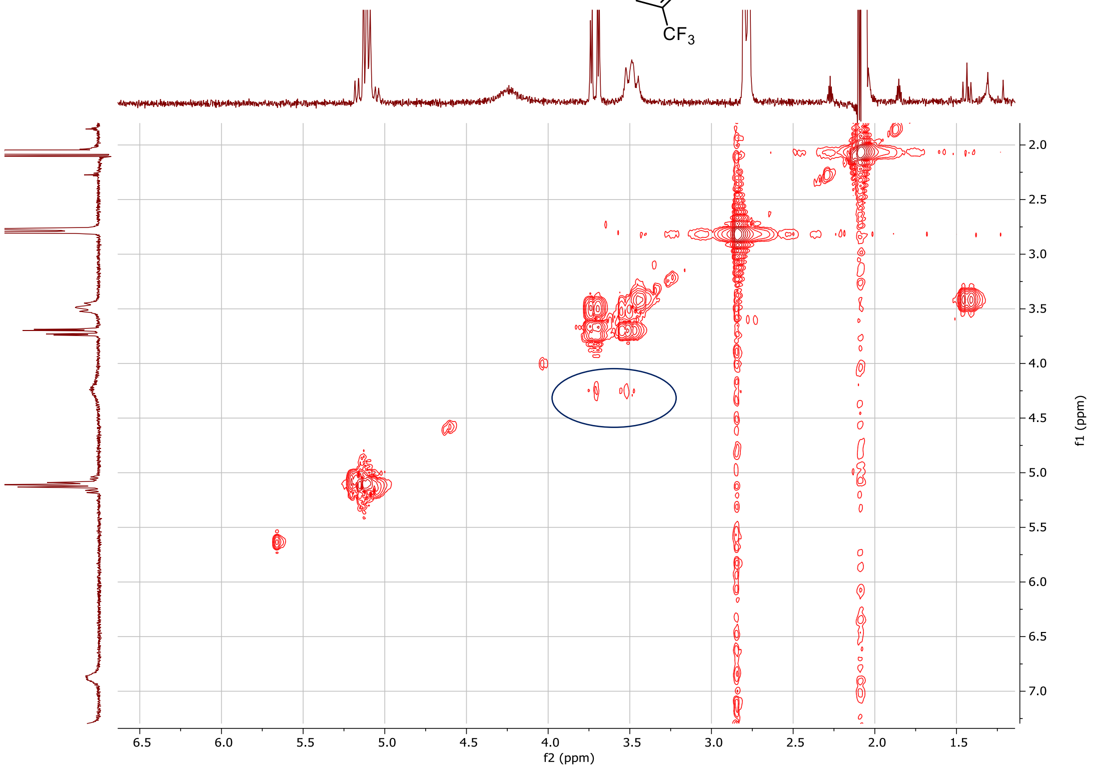

COSY (DMSO-*d*6)

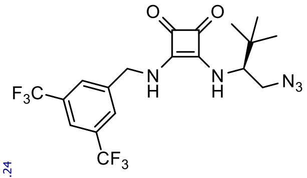

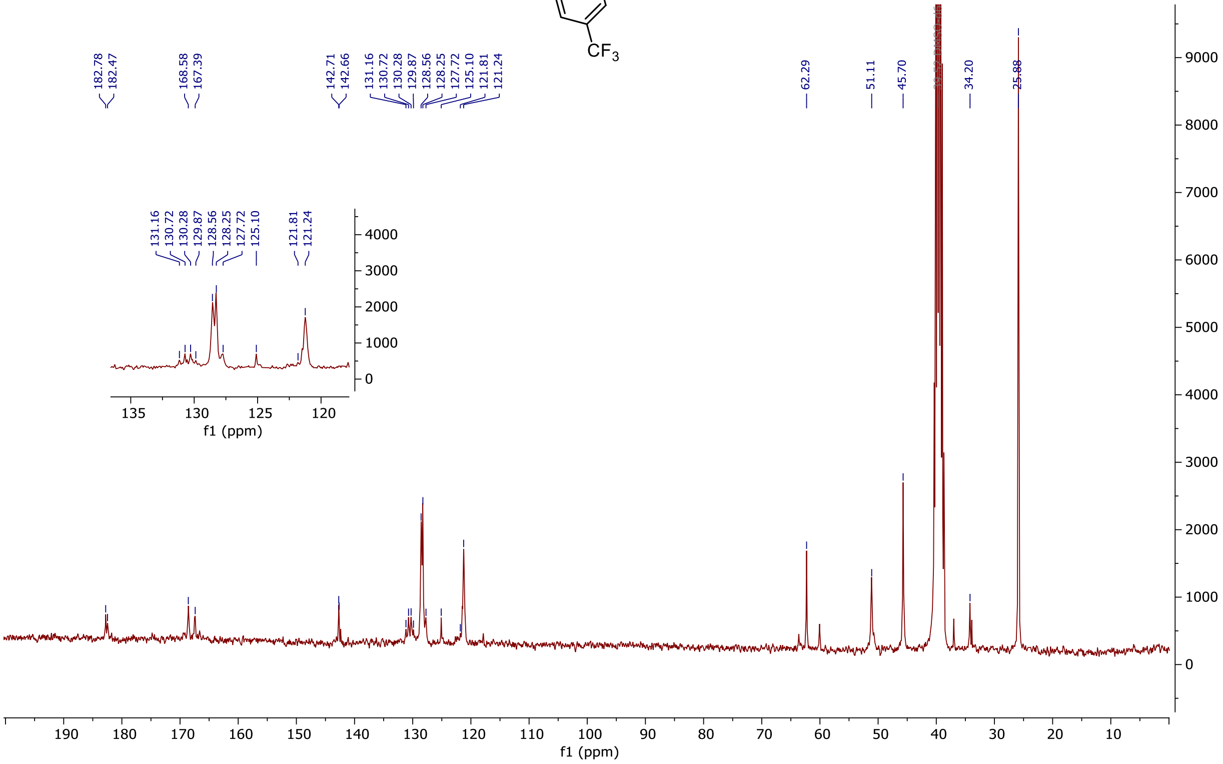

tert-butyl (3,3-dicyano-1,1,1-trifluoro-2-phenylpropan-2-yl)carbamate (16a)

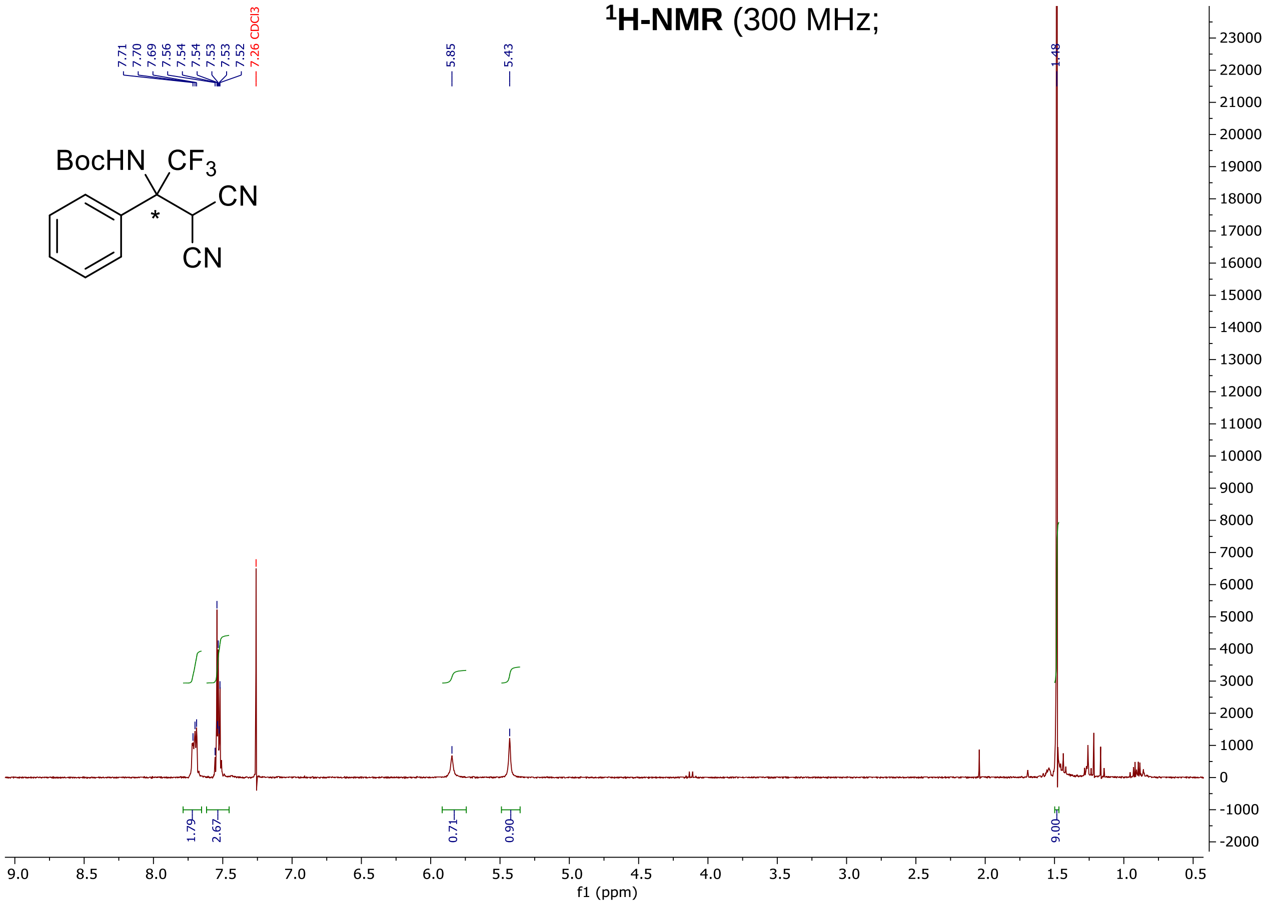

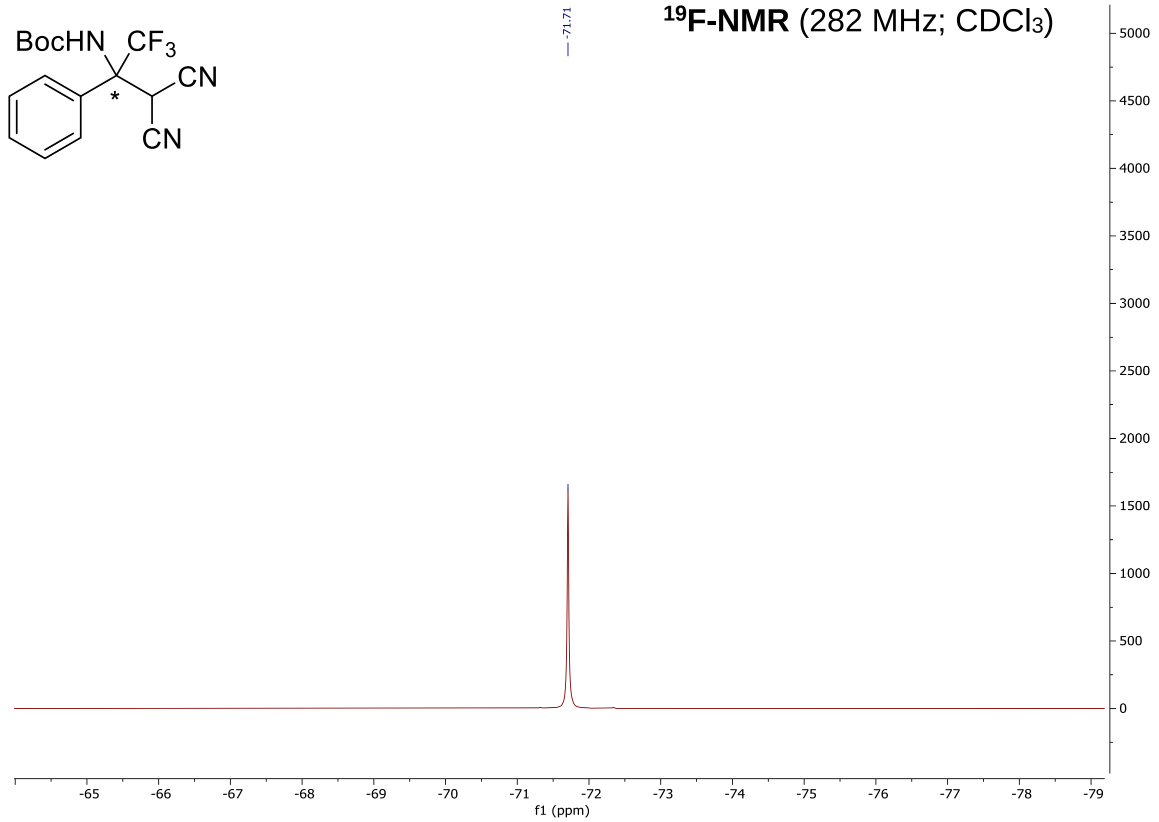

^{13}C-NMR (400 MHz;

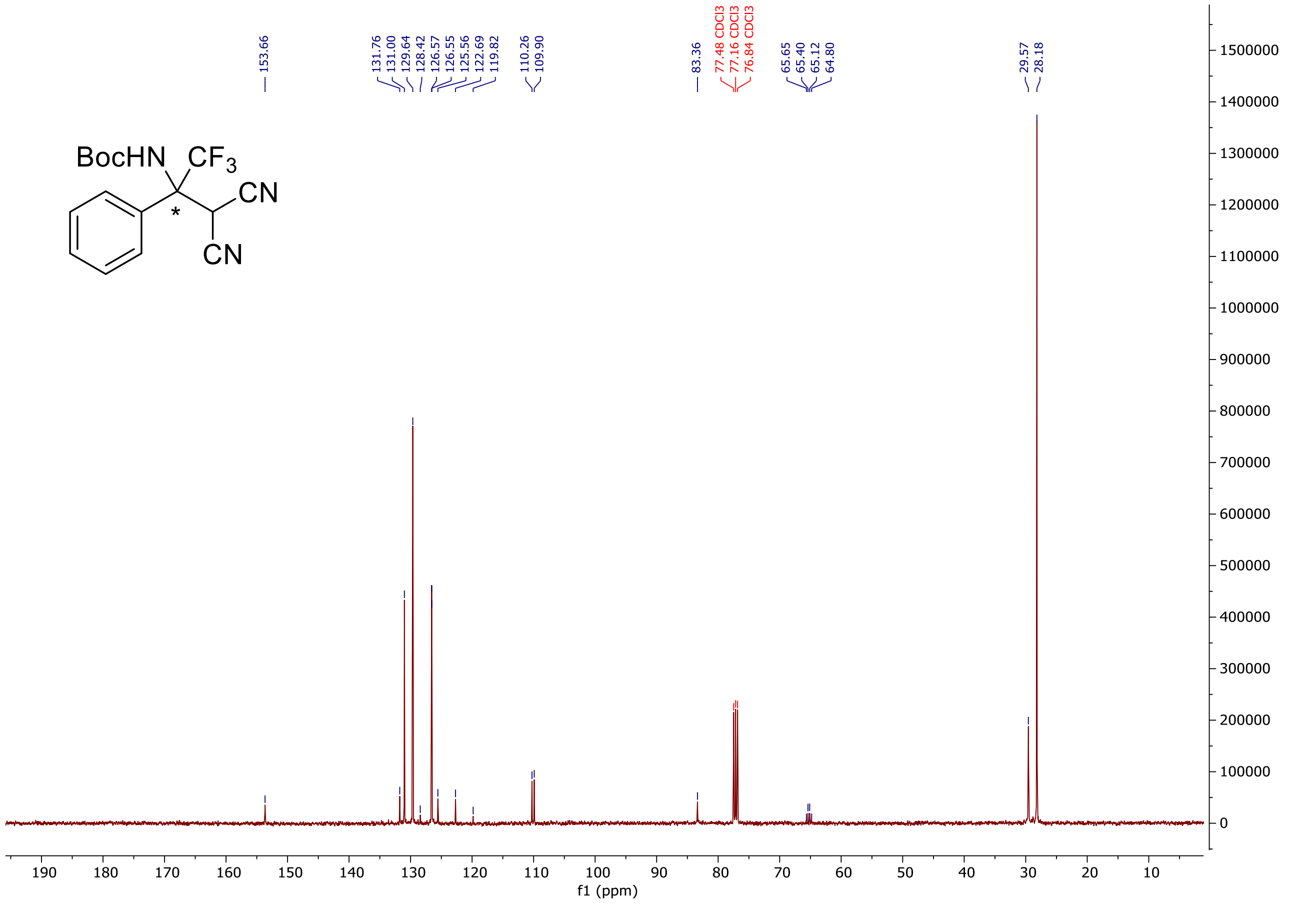

tert-butyl (2-(4-chlorophenyl)-3,3-dicyano-1,1,1-trifluoropropan-2-yl)carbamate (16b)

^{1}H-NMR (300 MHz;

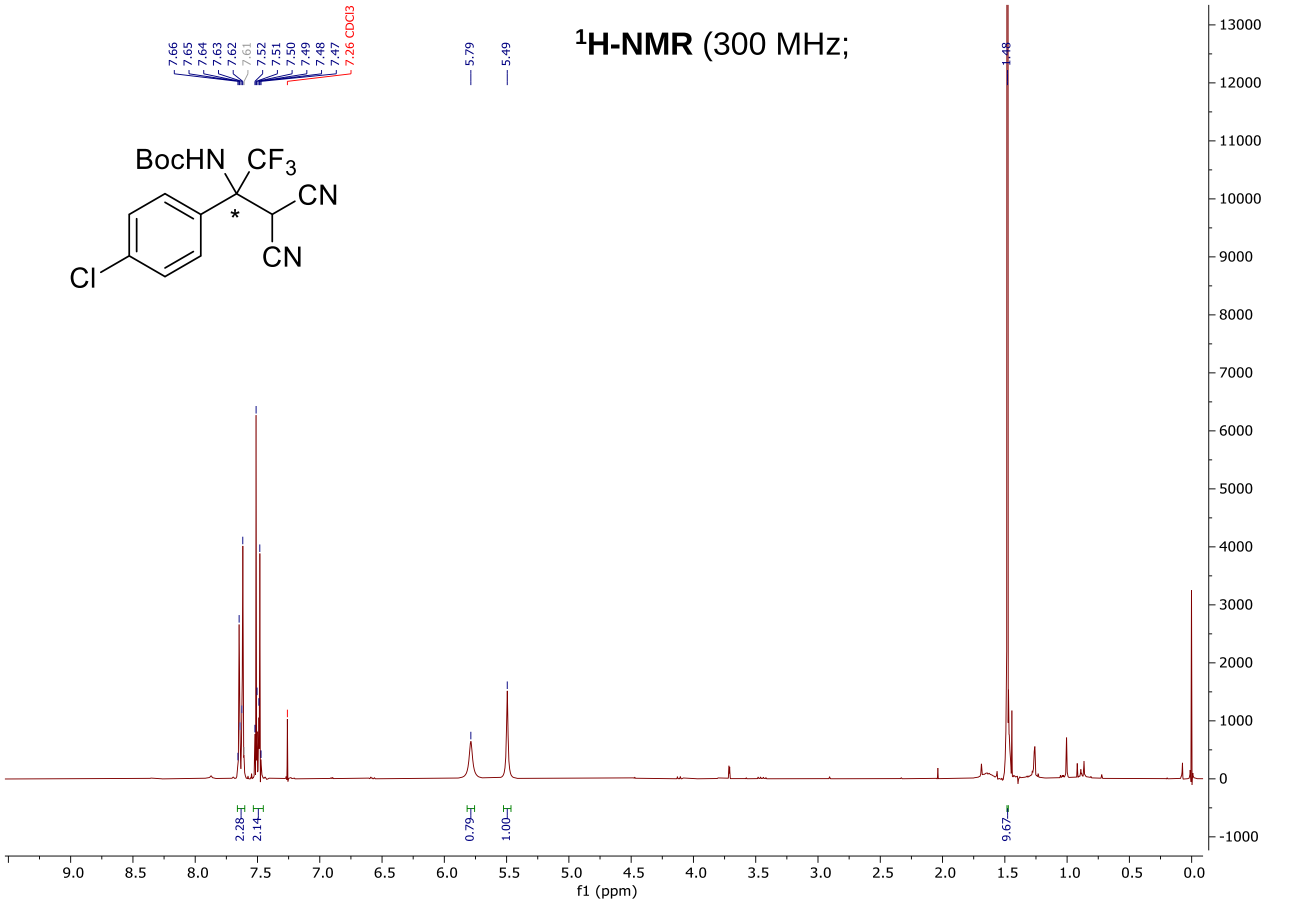

^{19}F-NMR (282 MHz; $CDCl_3$)

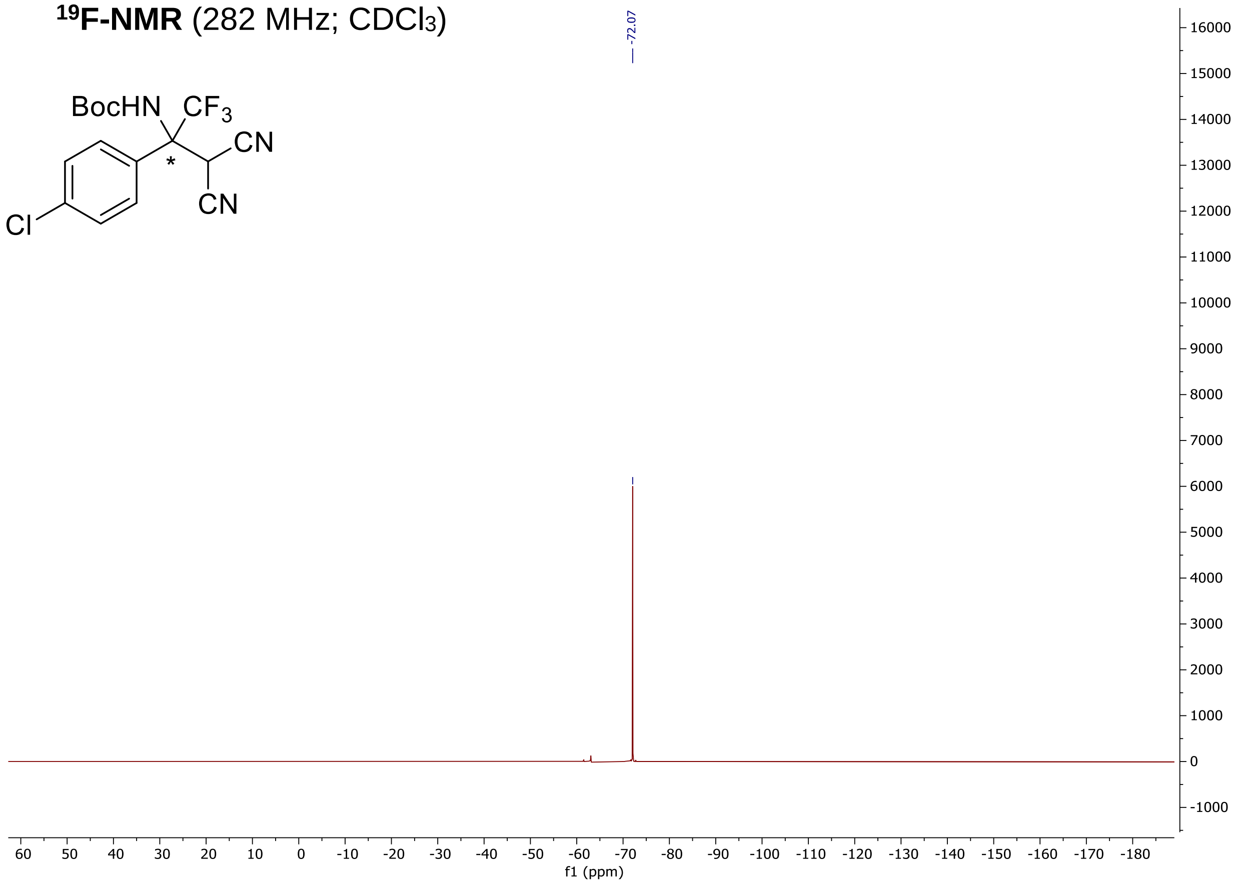

^{13}C-NMR (75 MHz; $CDCl_3$)

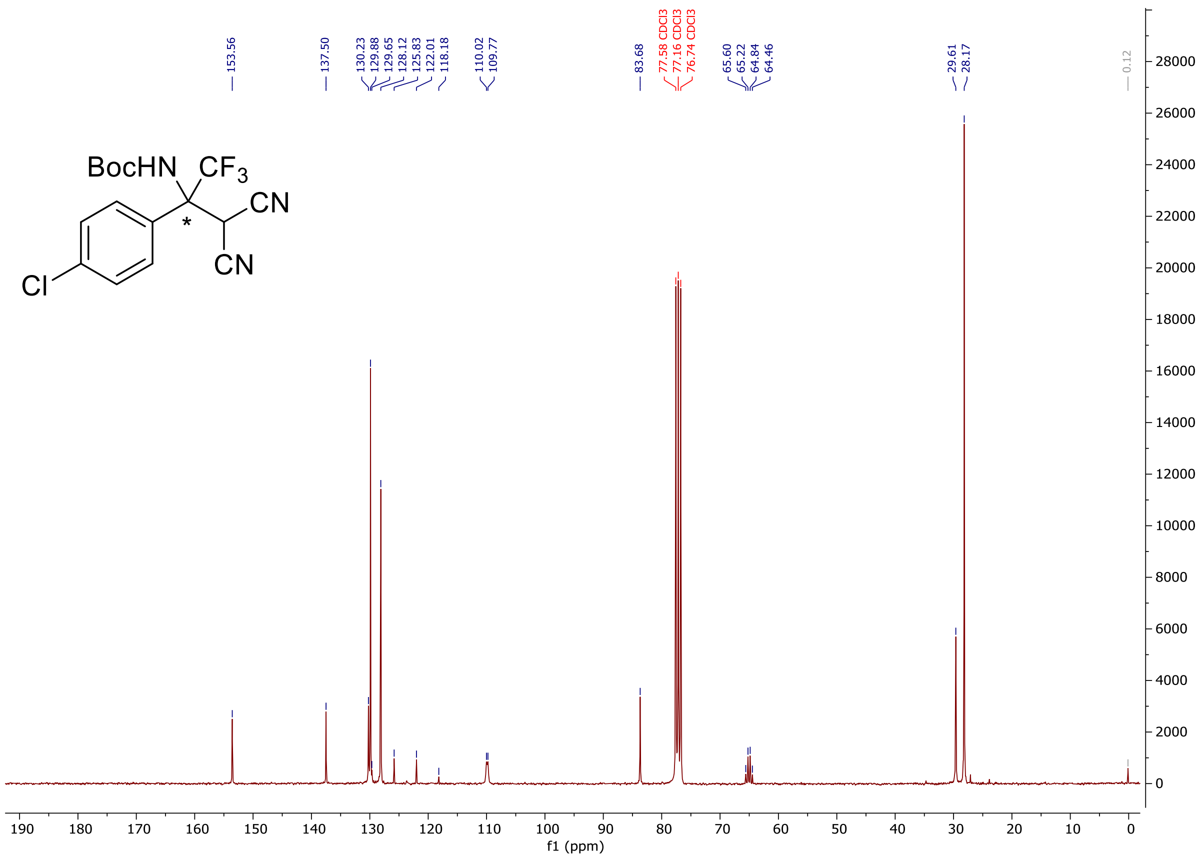

tert-butyl (3,3-dicyano-1,1,1-trifluoro-2-(4-methoxyphenyl)propan-2-yl)carbamate (16c)

^{1}H-NMR (300 MHz;

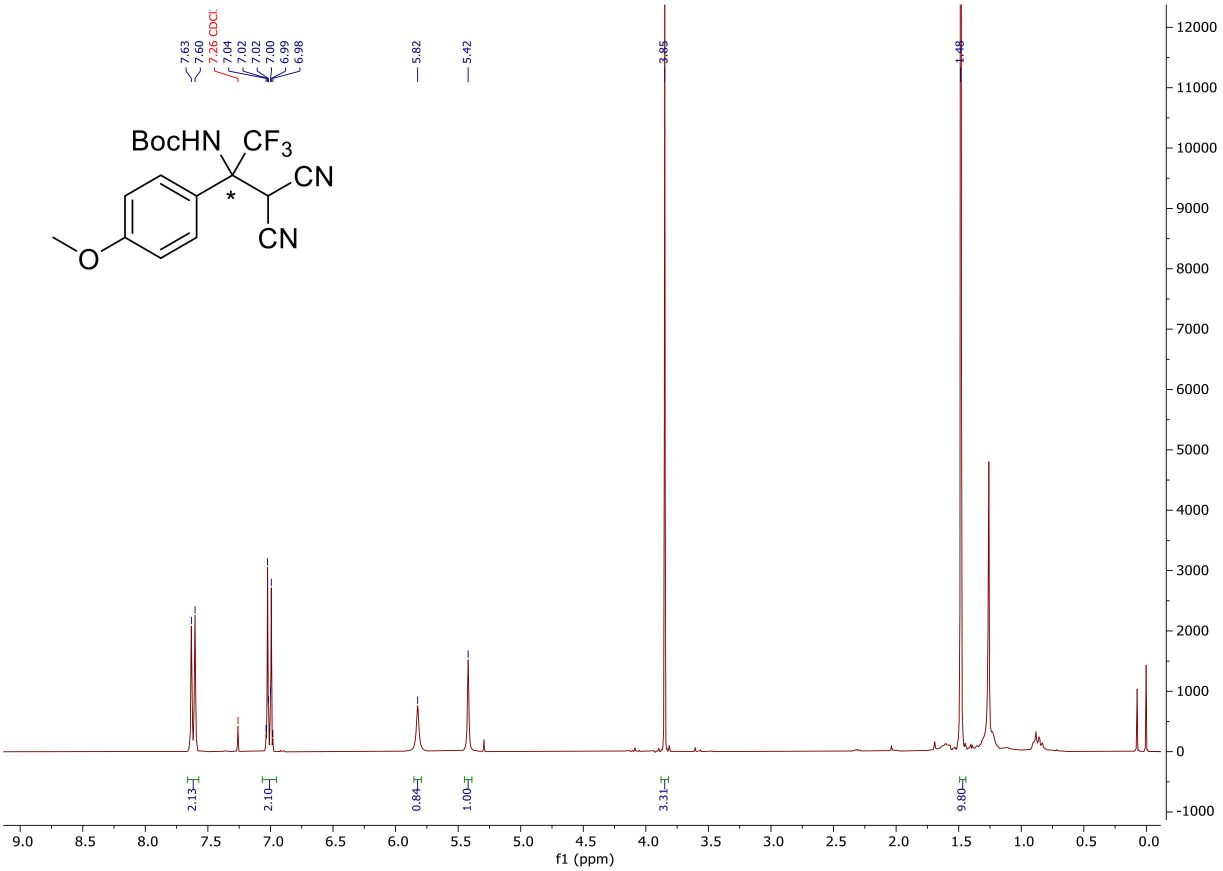

^{19}F-NMR (282 MHz; $CDCl_3$)

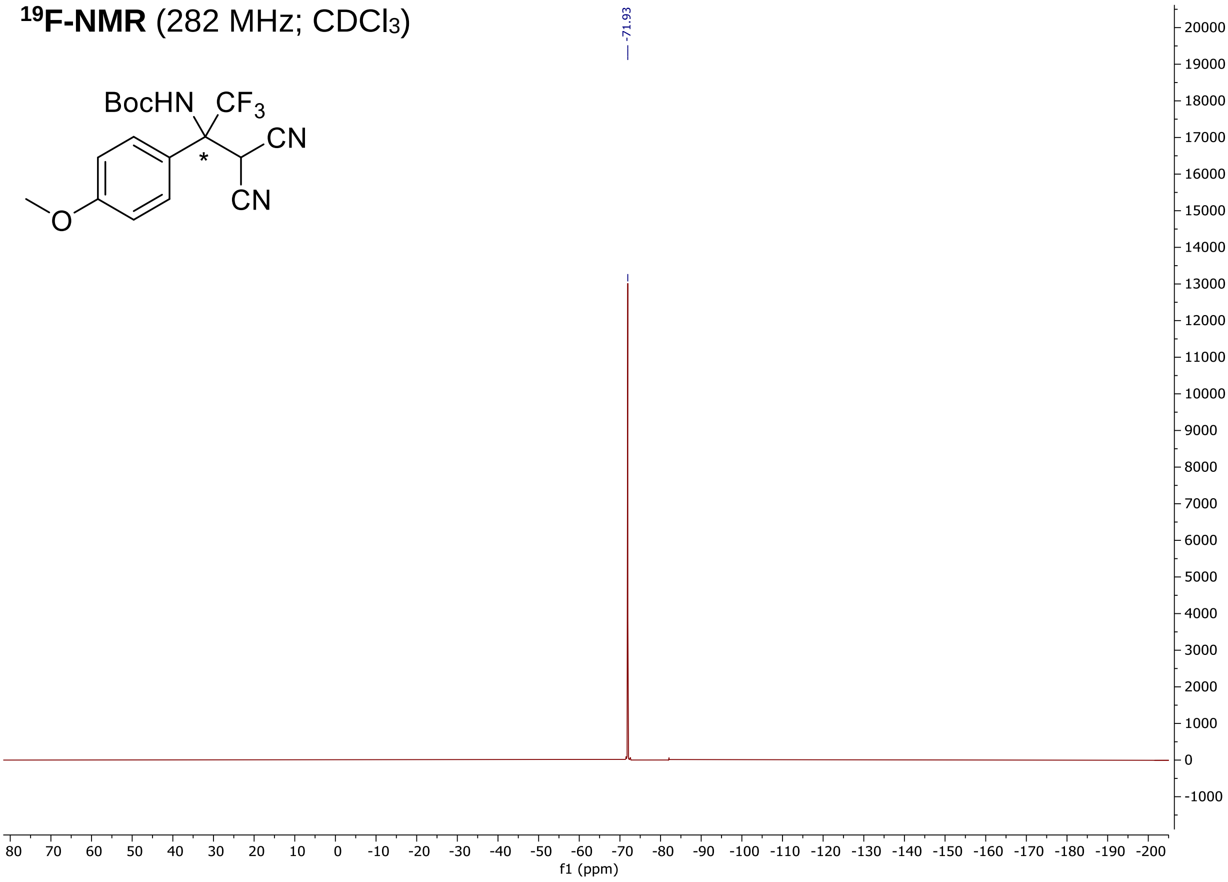

^{13}C-NMR (75 MHz; $CDCl_3$)

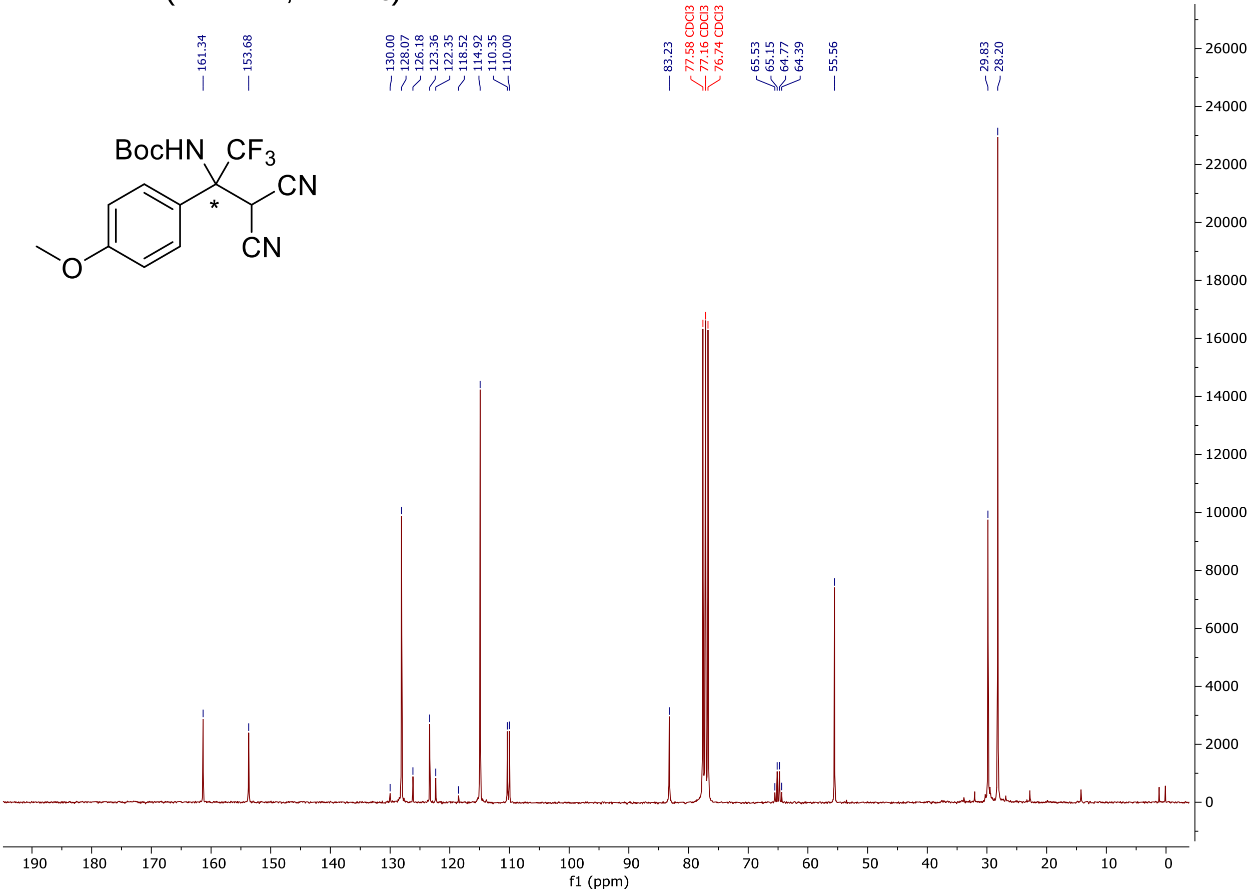

tert-butyl (2-(4-(tert-butyl)phenyl)-3,3-dicyano-1,1,1-trifluoropropan-2-yl)carbamate (16d)

^{1}H-NMR (300 MHz;

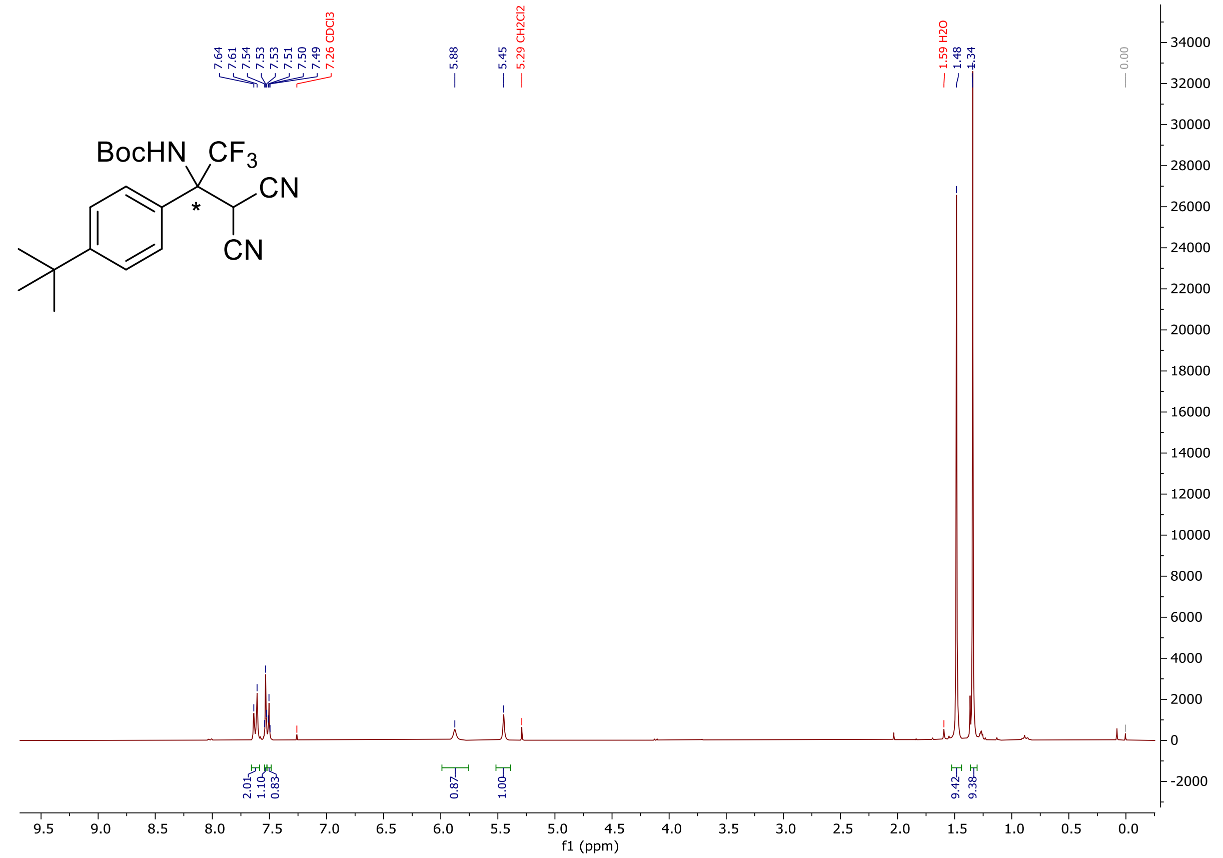

^{19}F-NMR (282 MHz; $CDCl_3$)

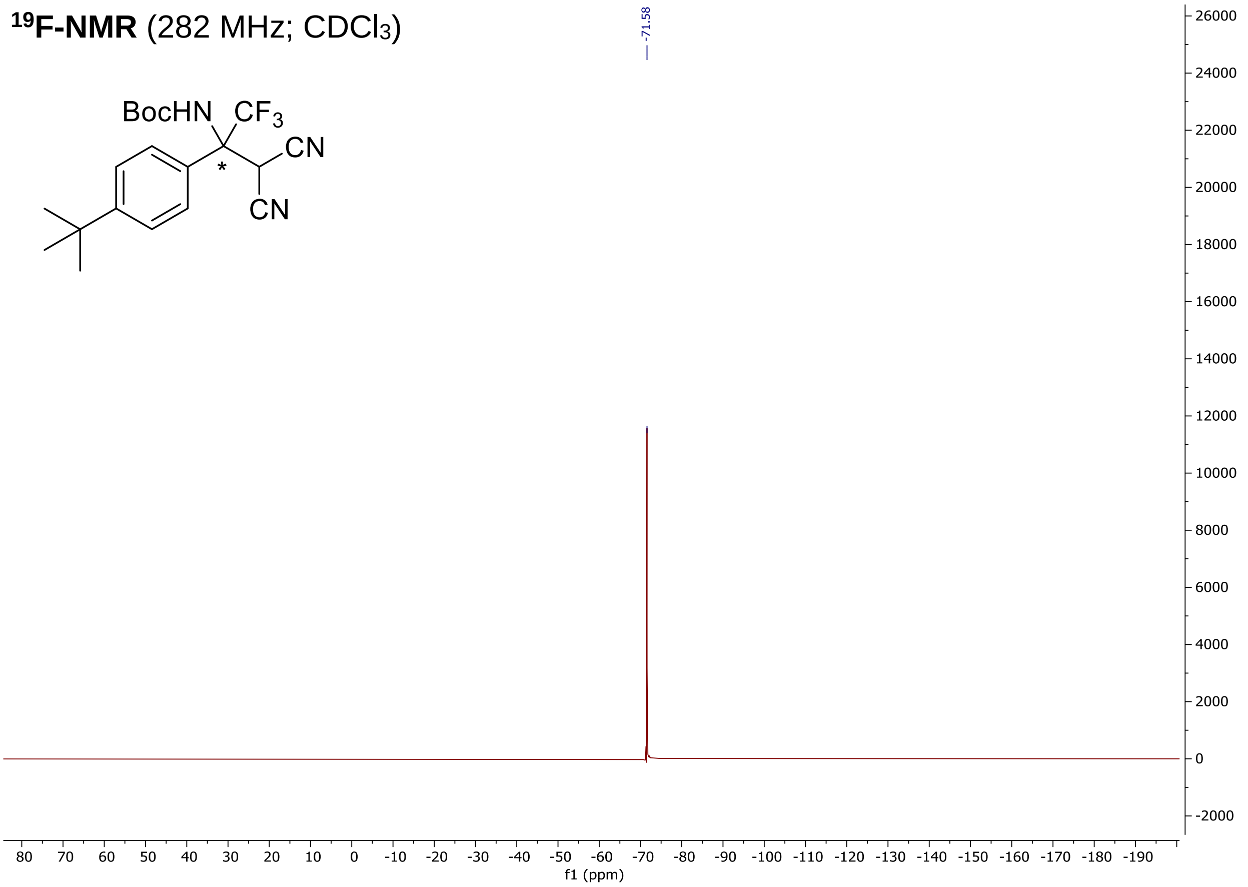

^{13}C-NMR (75 MHz; $CDCl_3$)

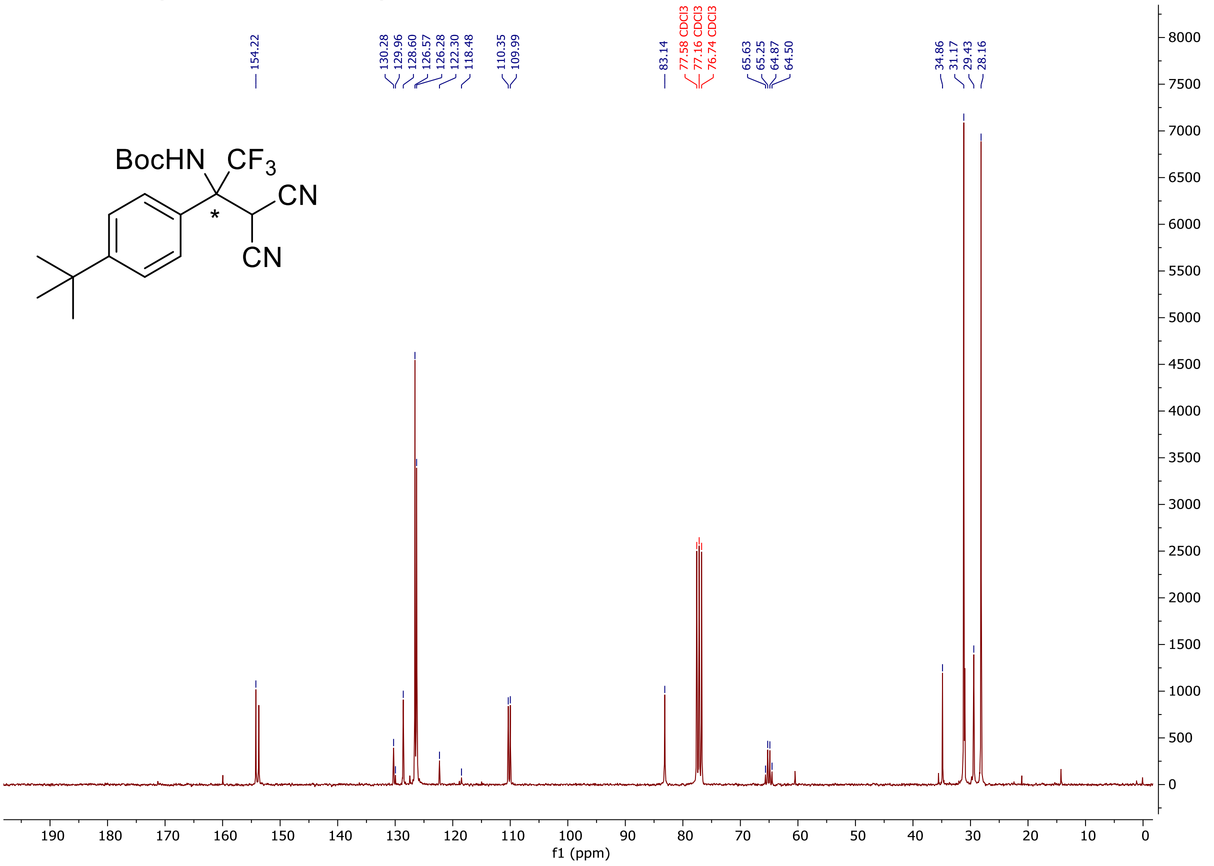

tert-butyl (3,3-dicyano-1,1,1-trifluoro-2-(m-tolyl)propan-2-yl)carbamate (16e)

^{1}H-NMR (300 MHz;

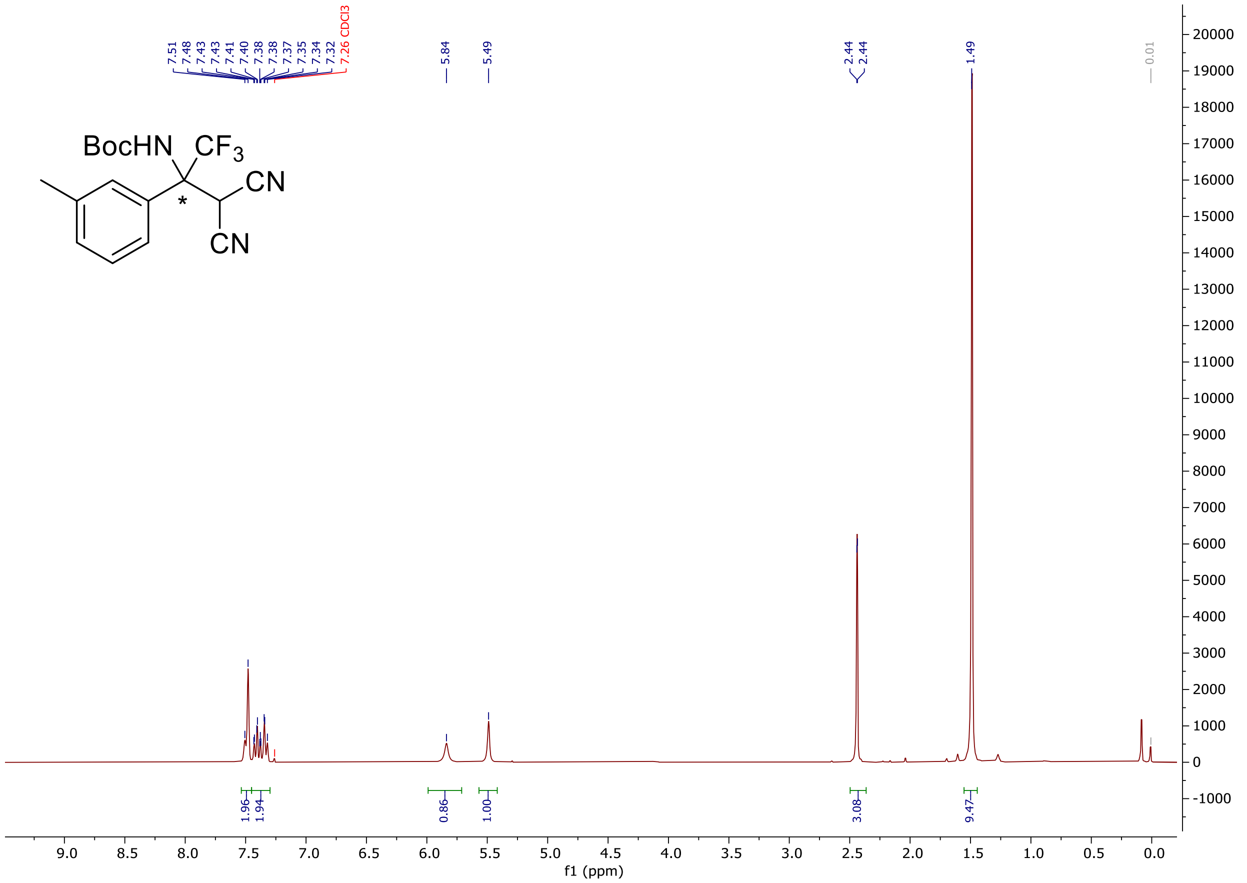

^{19}F-NMR (282 MHz; $CDCl_3$)

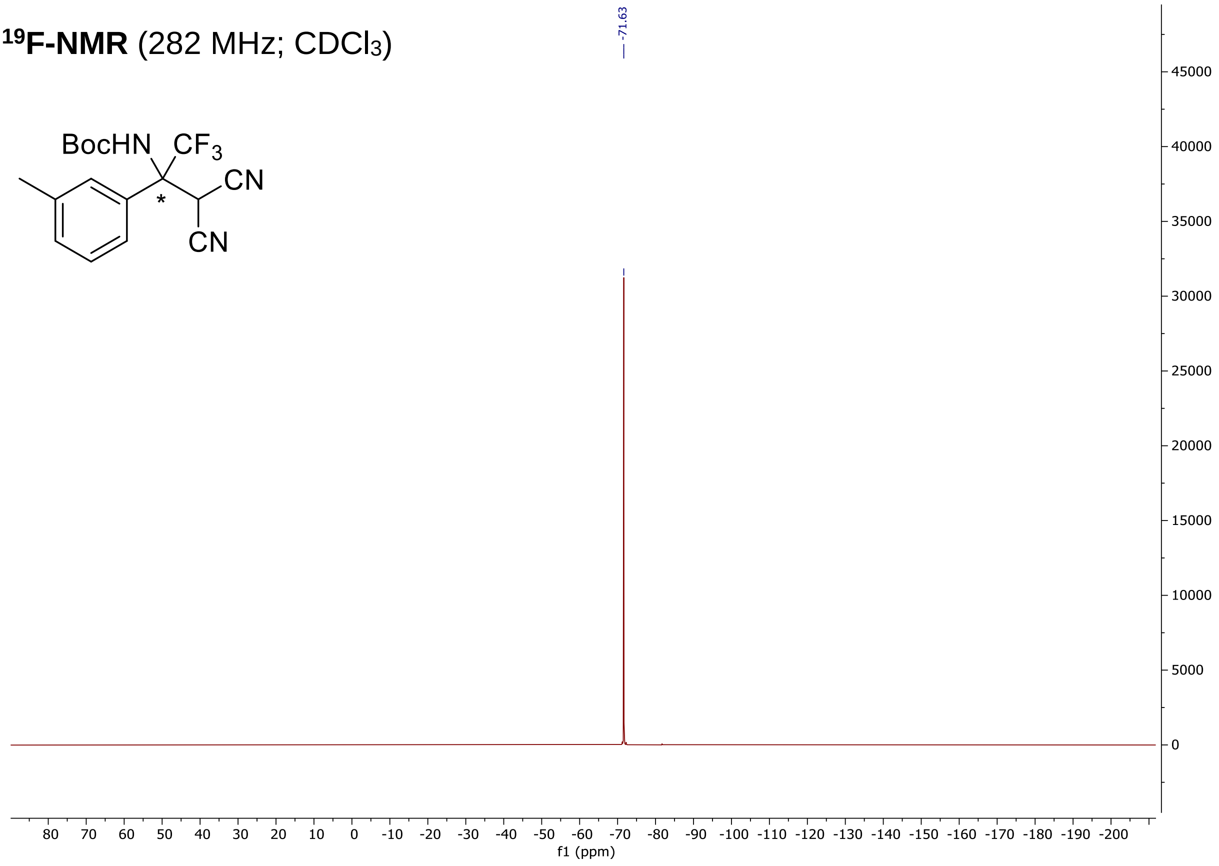

^{13}C-NMR (75 MHz; CDCl3)

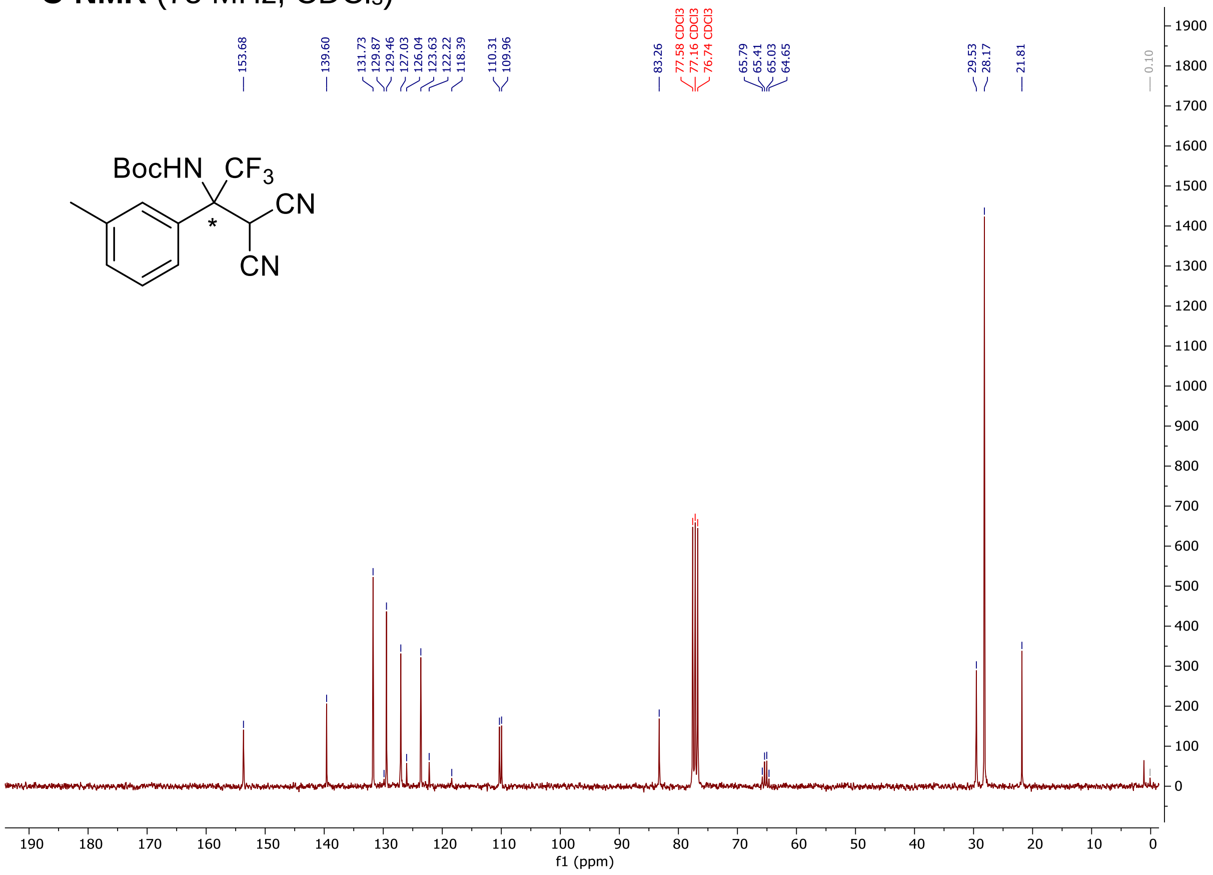

tert-butyl (2-(3-chloro-5-fluorophenyl)-3,3-dicyano-1,1,1-trifluoropropan-2-yl)carbamate (16f)

^{1}H-NMR (300 MHz;

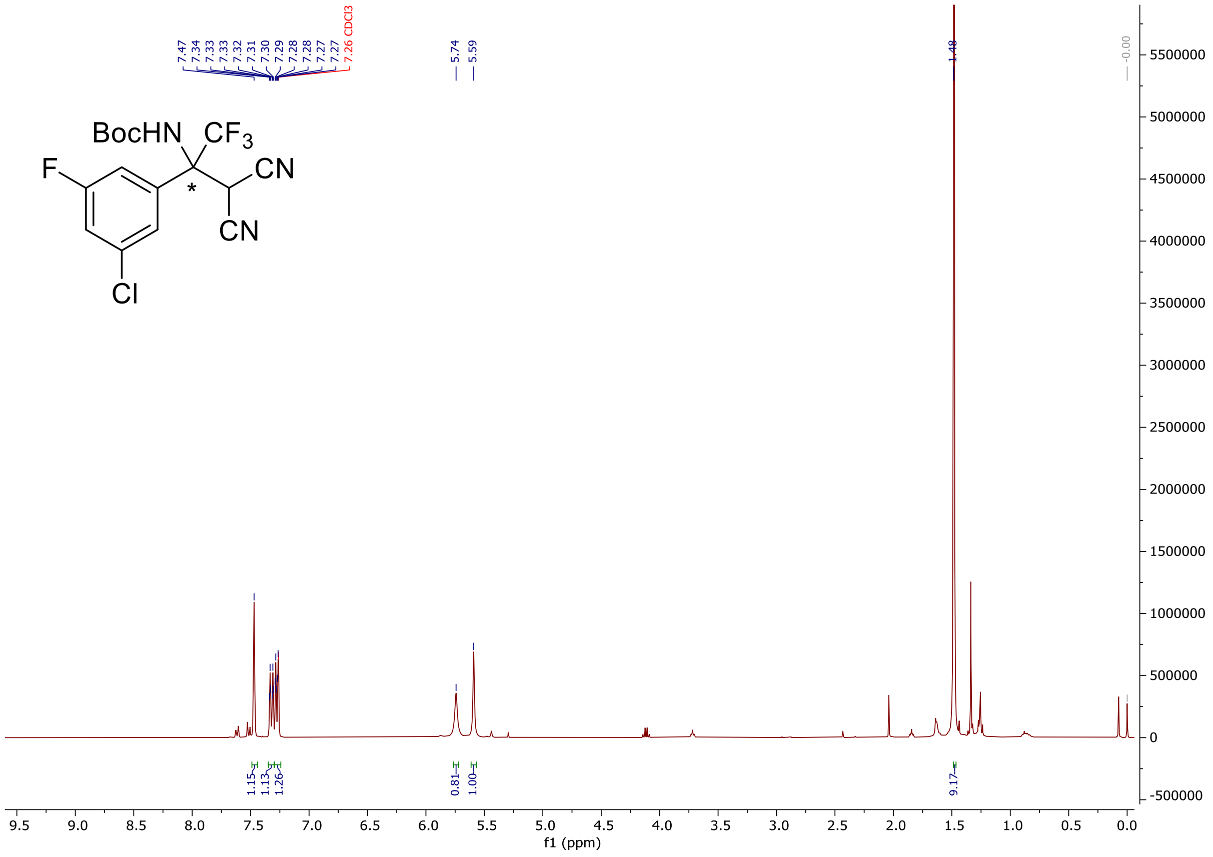

^{19}F-NMR (282 MHz; $CDCl_3$)

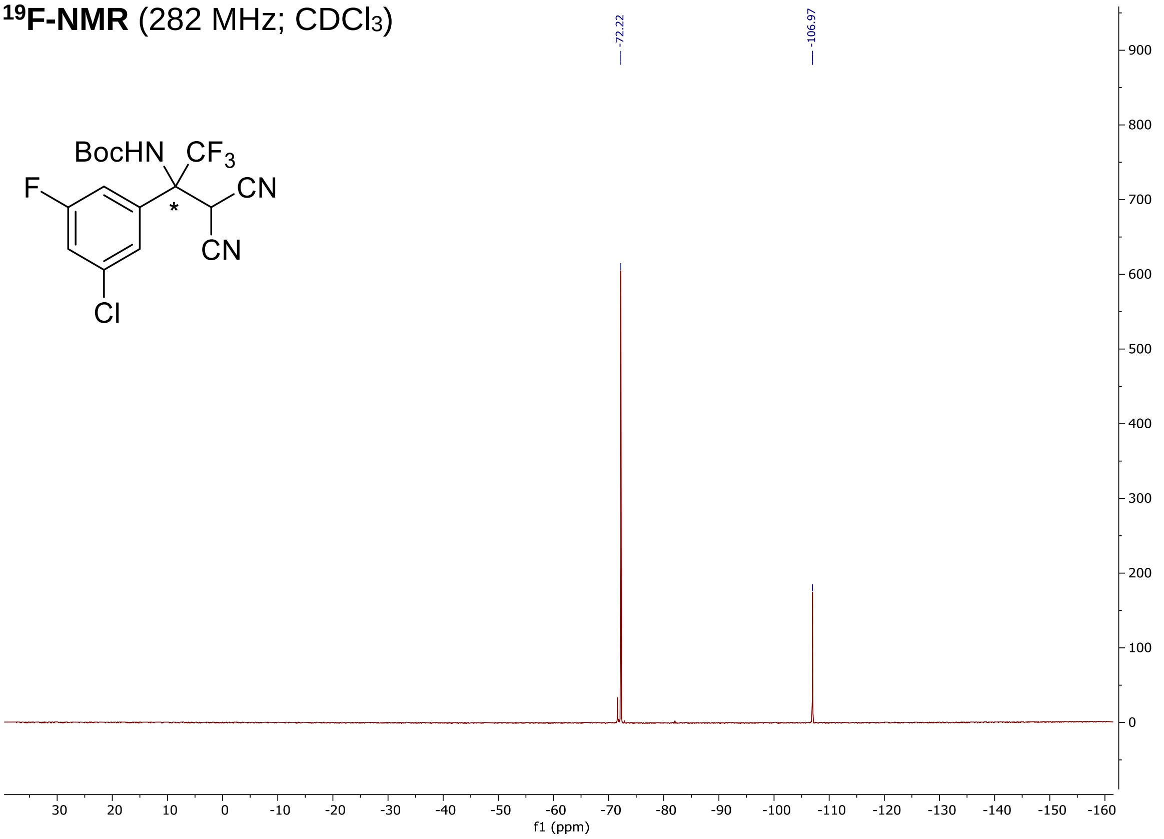

^{13}C-NMR (101 MHz;

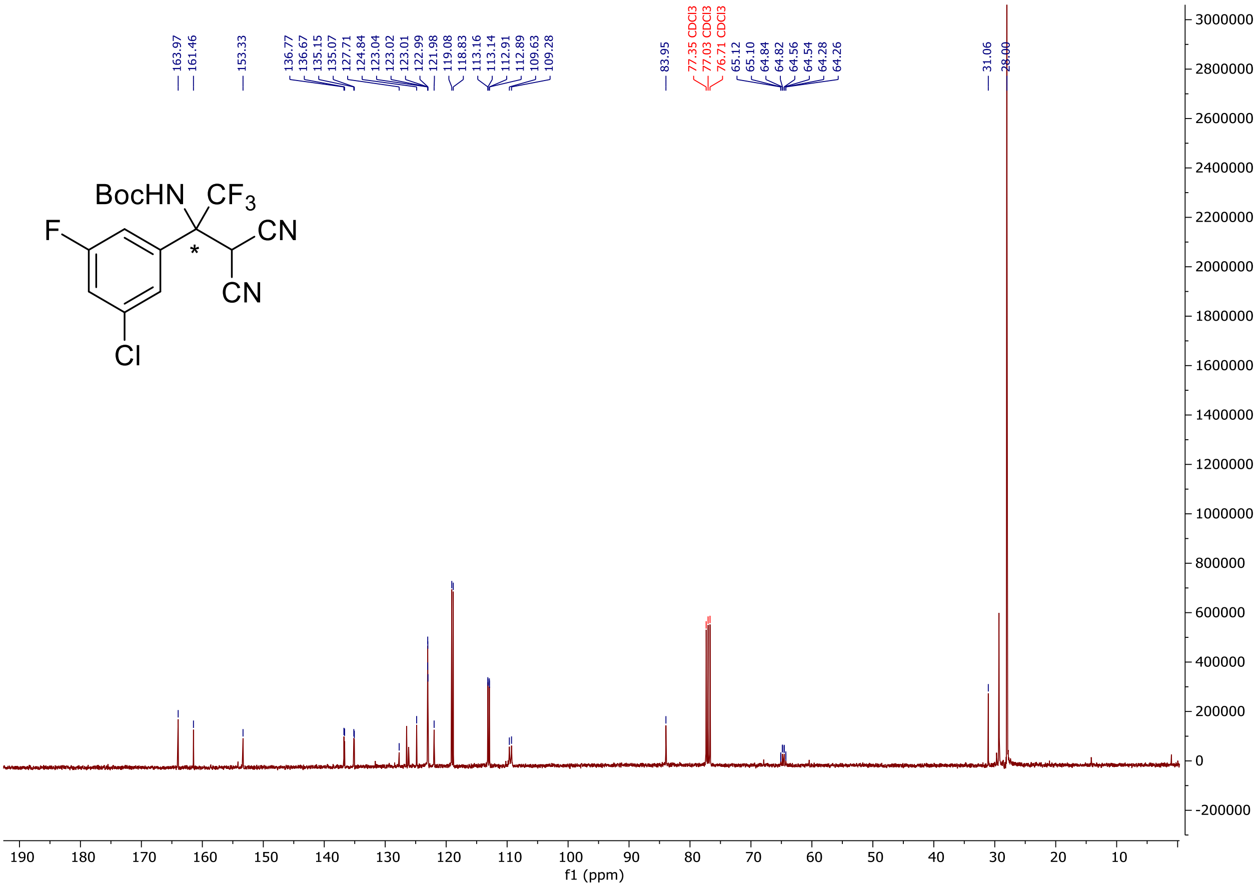

HSQC ($CDCl_3$)

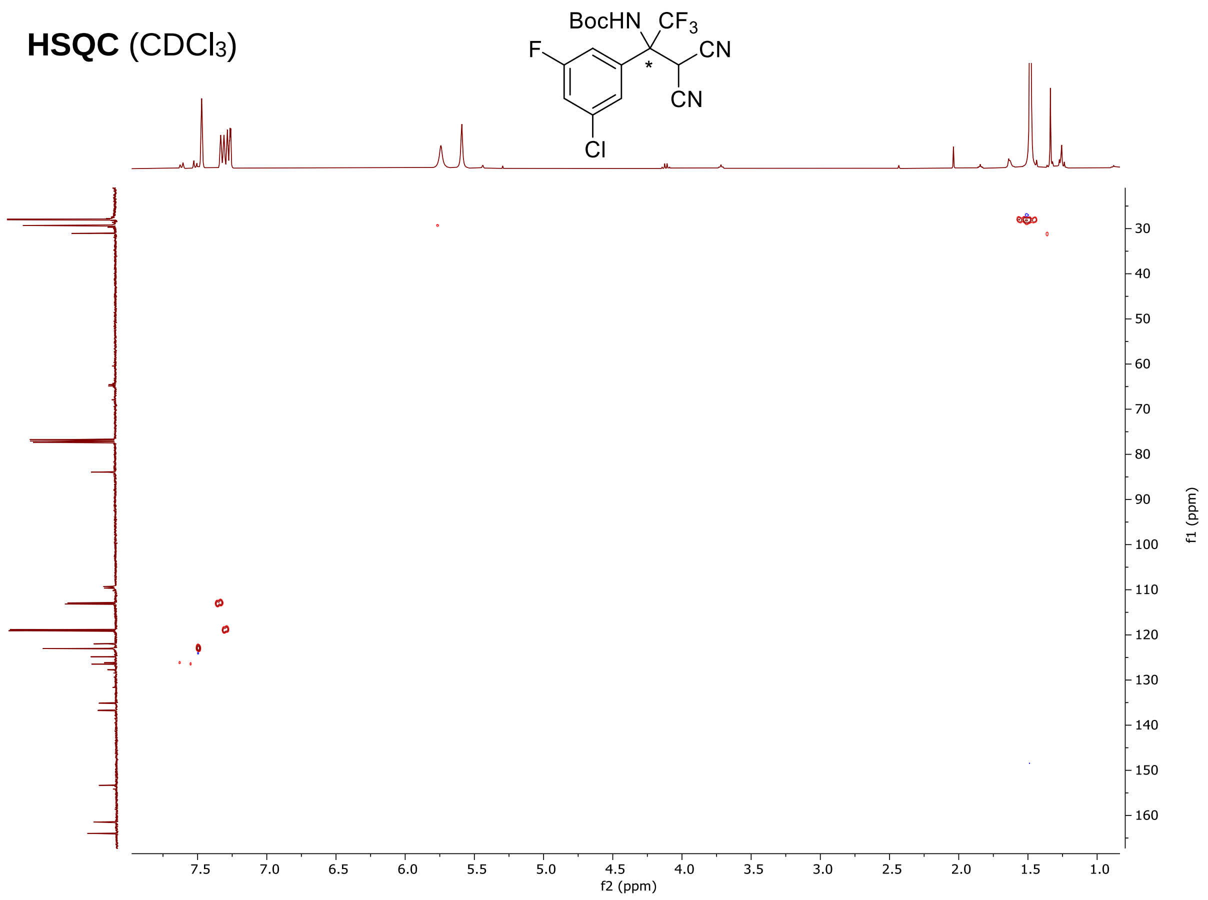

methyl 2-((tert-butoxycarbonyl)amino)-3,3,3-trifluoro-2-phenylpropanoate (17a)

^{1}H-NMR (300 MHz;

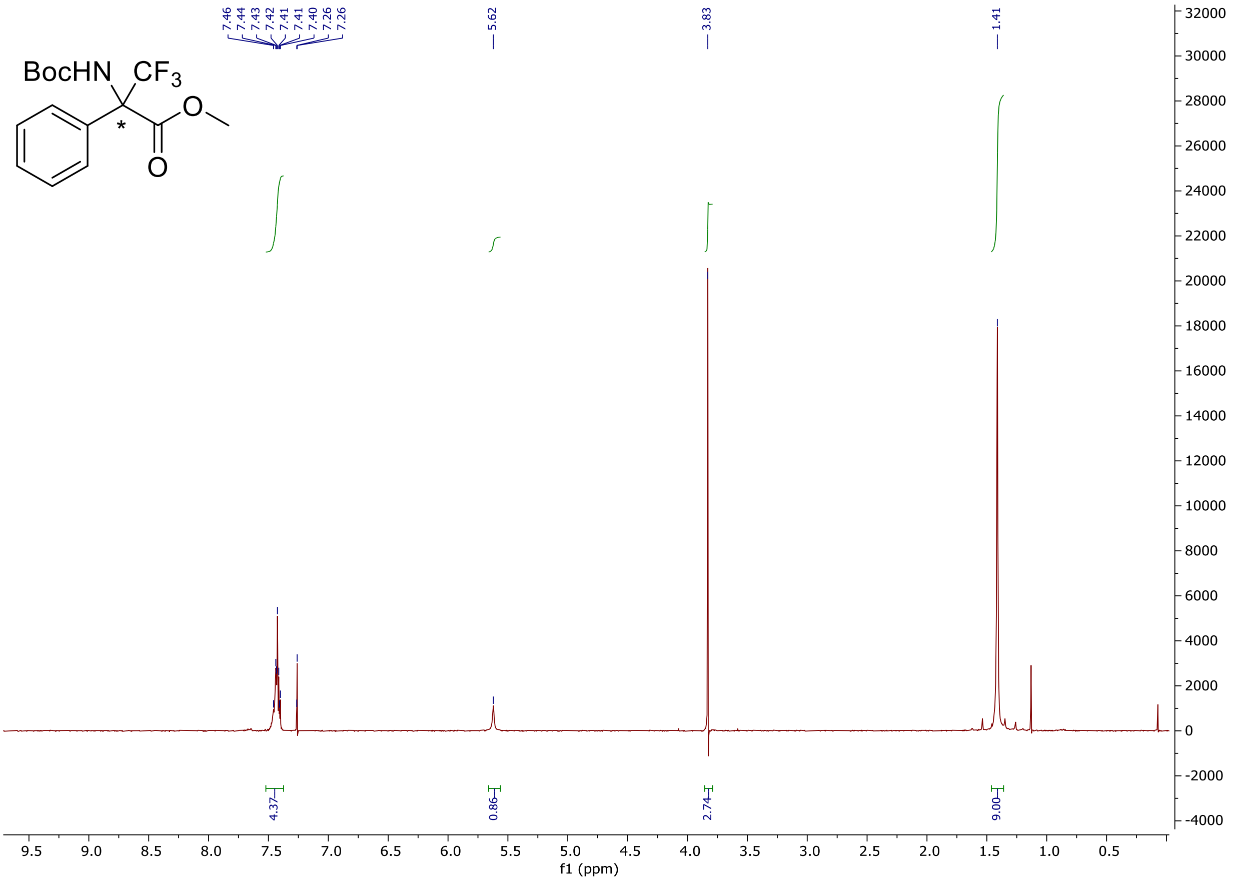

^{19}F-NMR (282 MHz; $CDCl_3$)

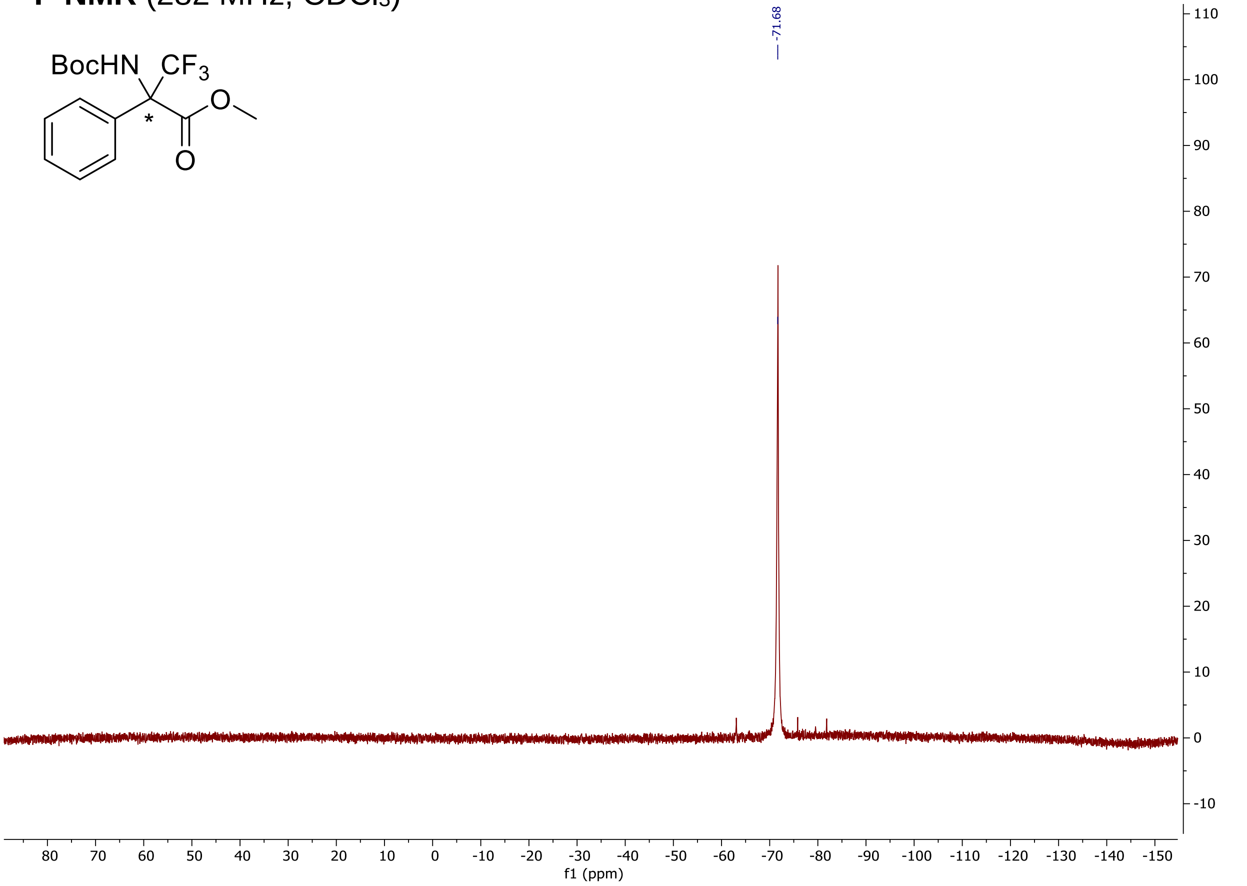

^{13}C-NMR (75 MHz; $CDCl_3$)

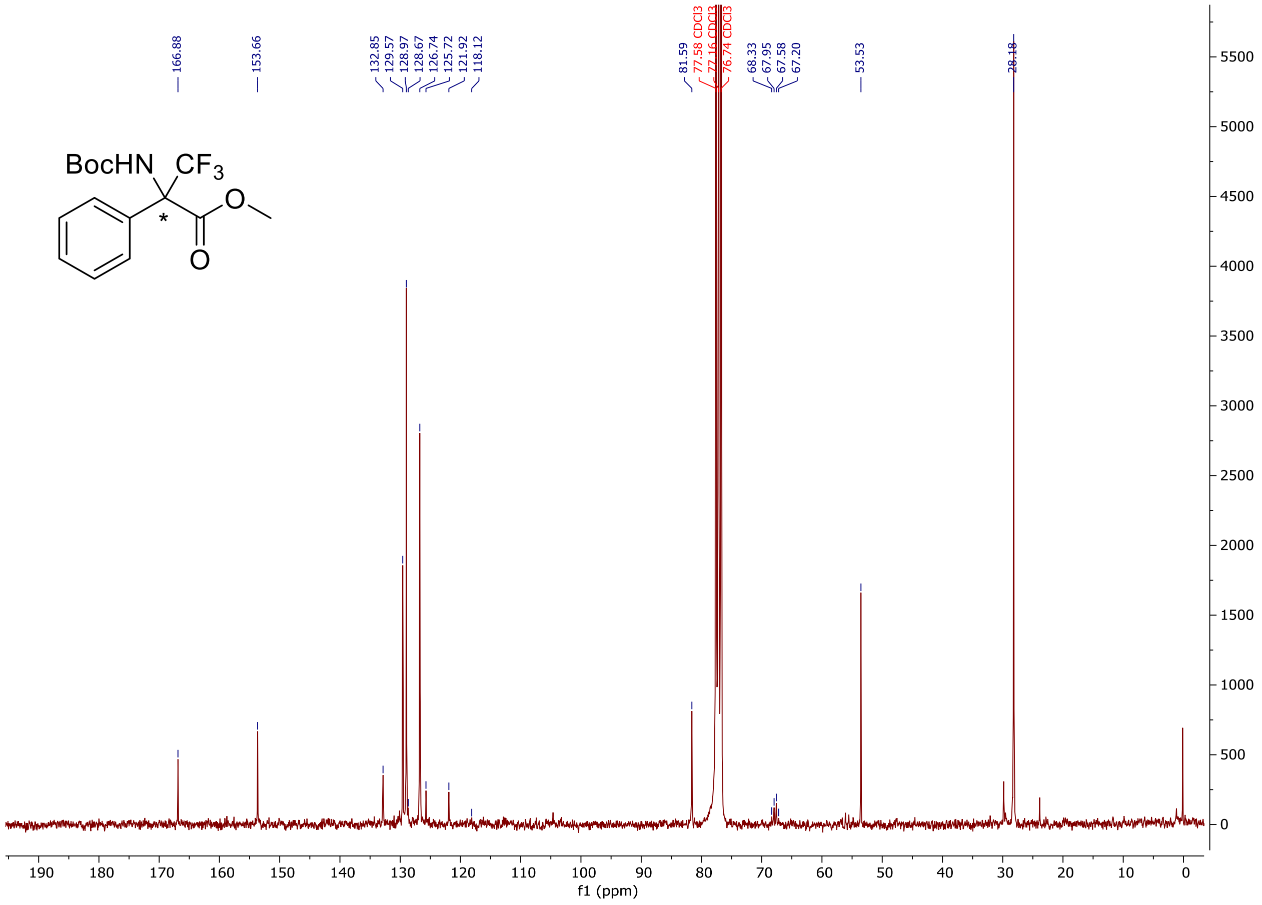

methyl 2-((tert-butoxycarbonyl)amino)-2-(4-chlorophenyl)-3,3,3-trifluoropropanoate (17b)

^{1}H-NMR (300 MHz;

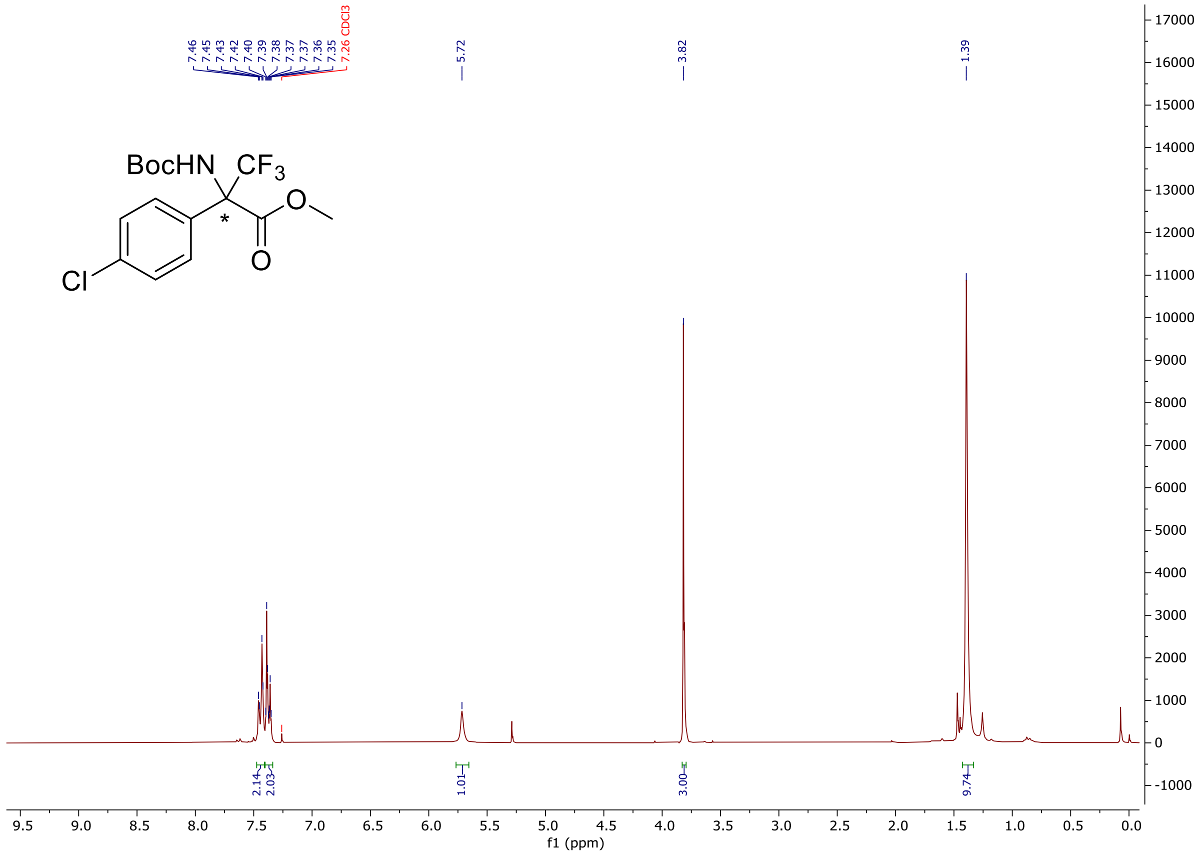

^{19}F-NMR (282 MHz; $CDCl_3$)

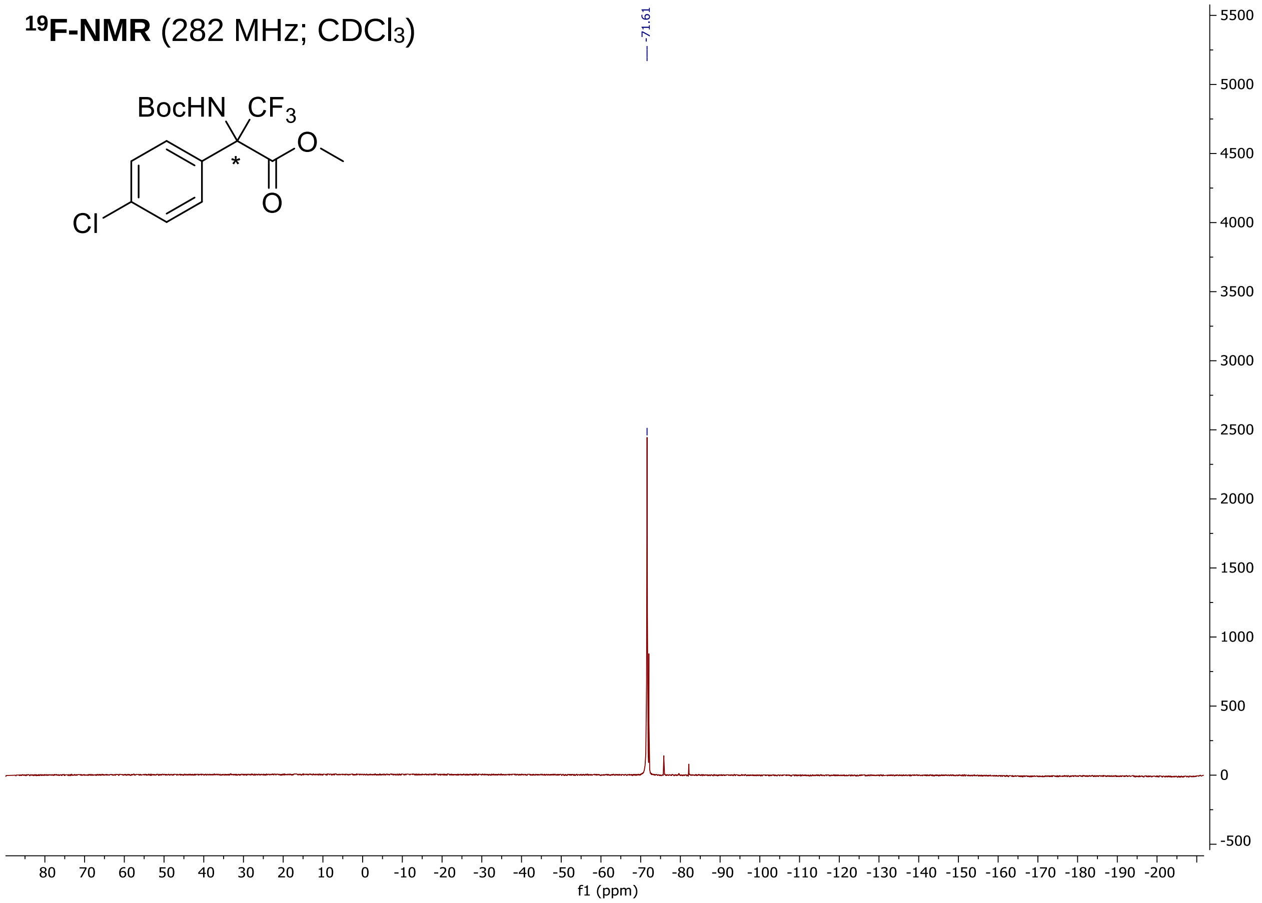

^{13}C-NMR (101 MHz;

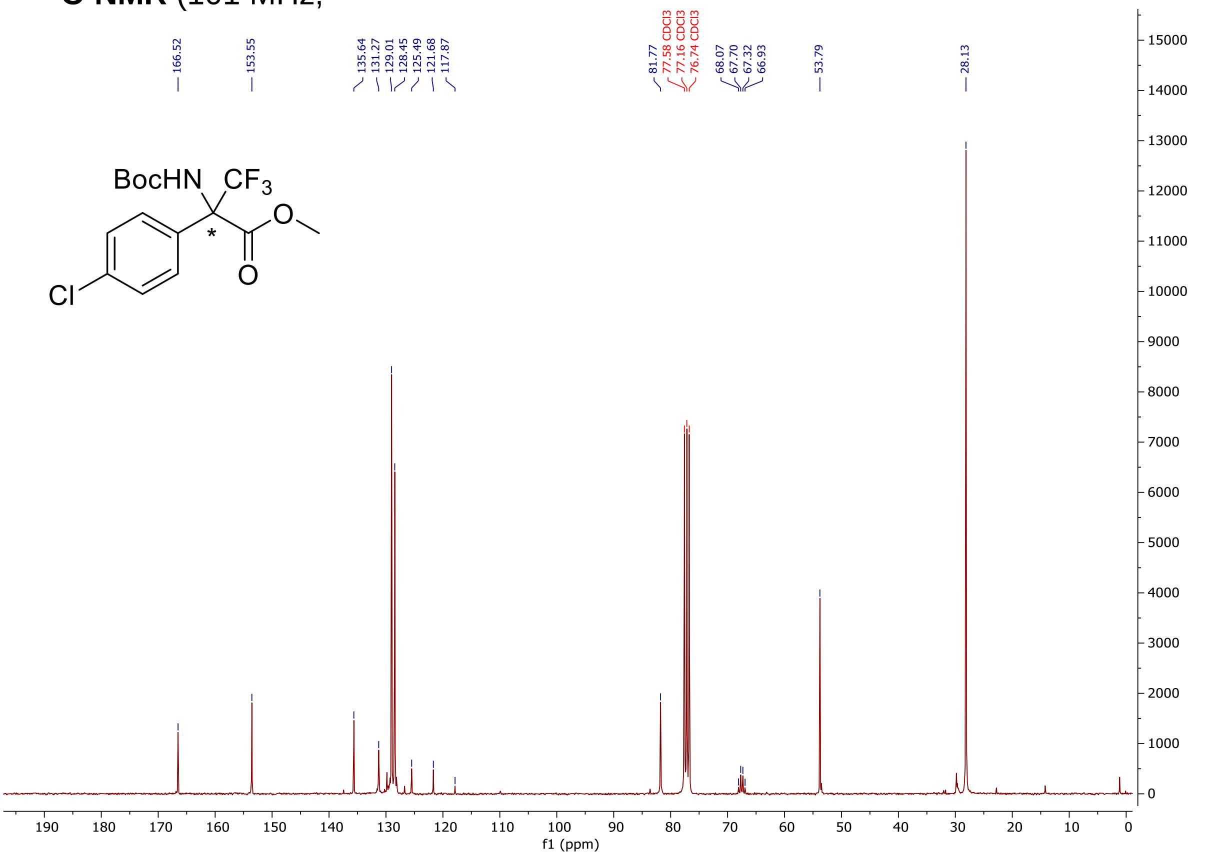

methyl 2-((tert-butoxycarbonyl)amino)-3,3,3-trifluoro-2-(4-methoxyphenyl)propanoate (17c)

^{1}H-NMR (300 MHz;

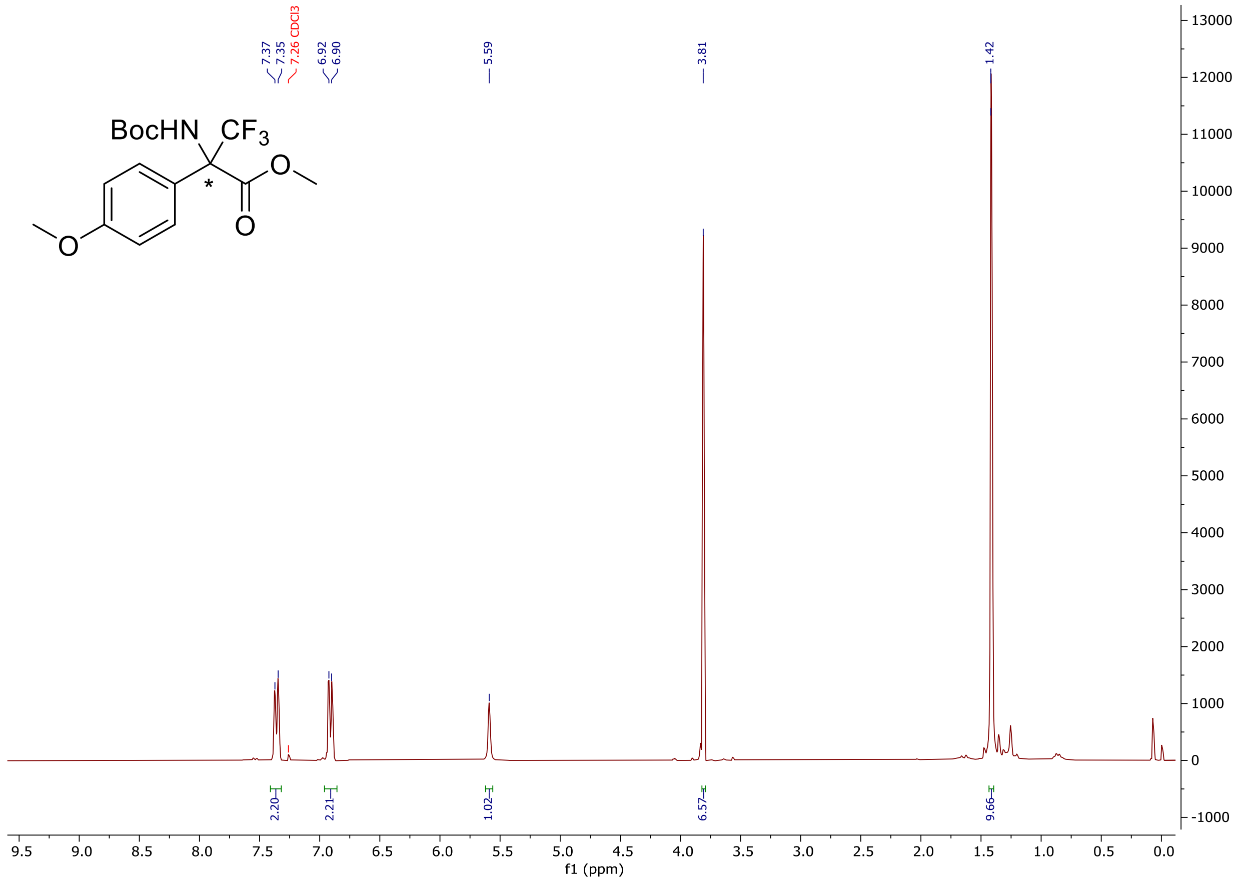

19F-NMR (282 MHz; $CDCl_3$)

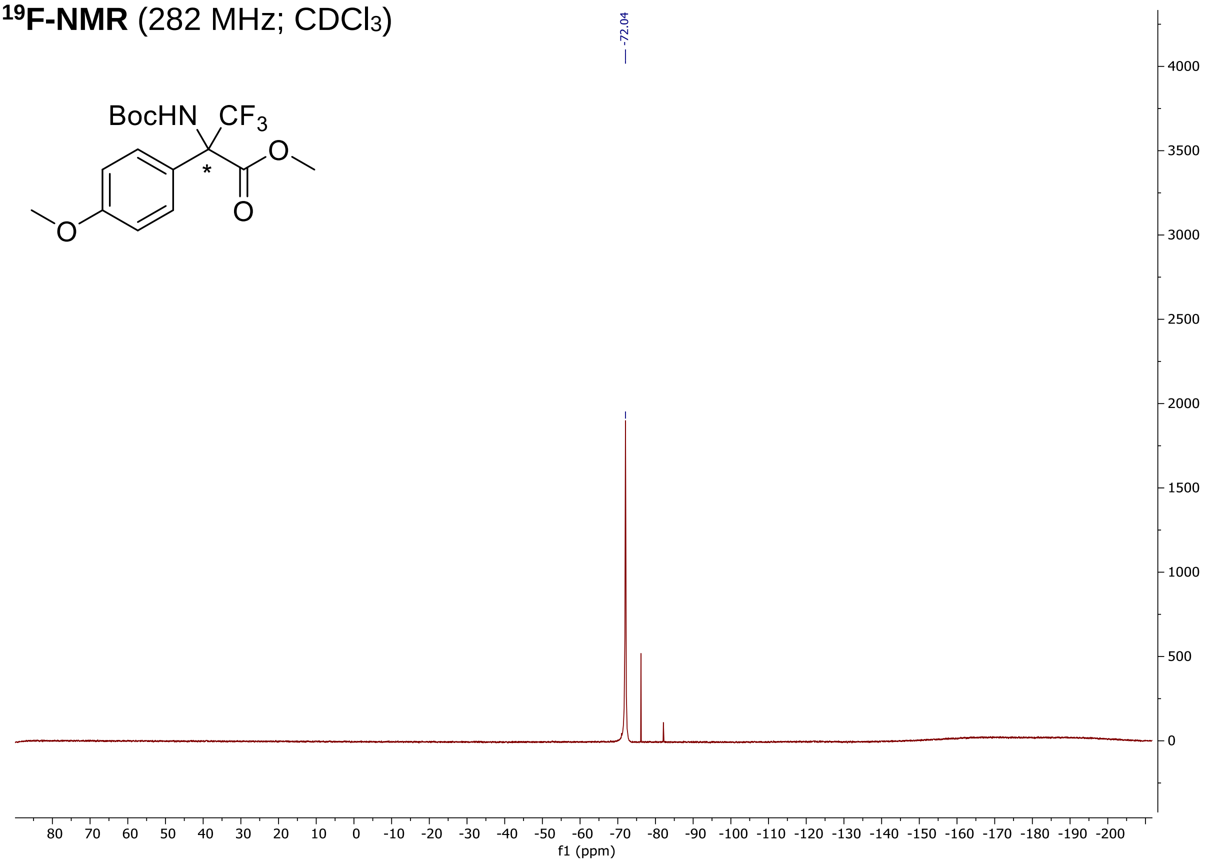

13C-NMR (101 MHz;

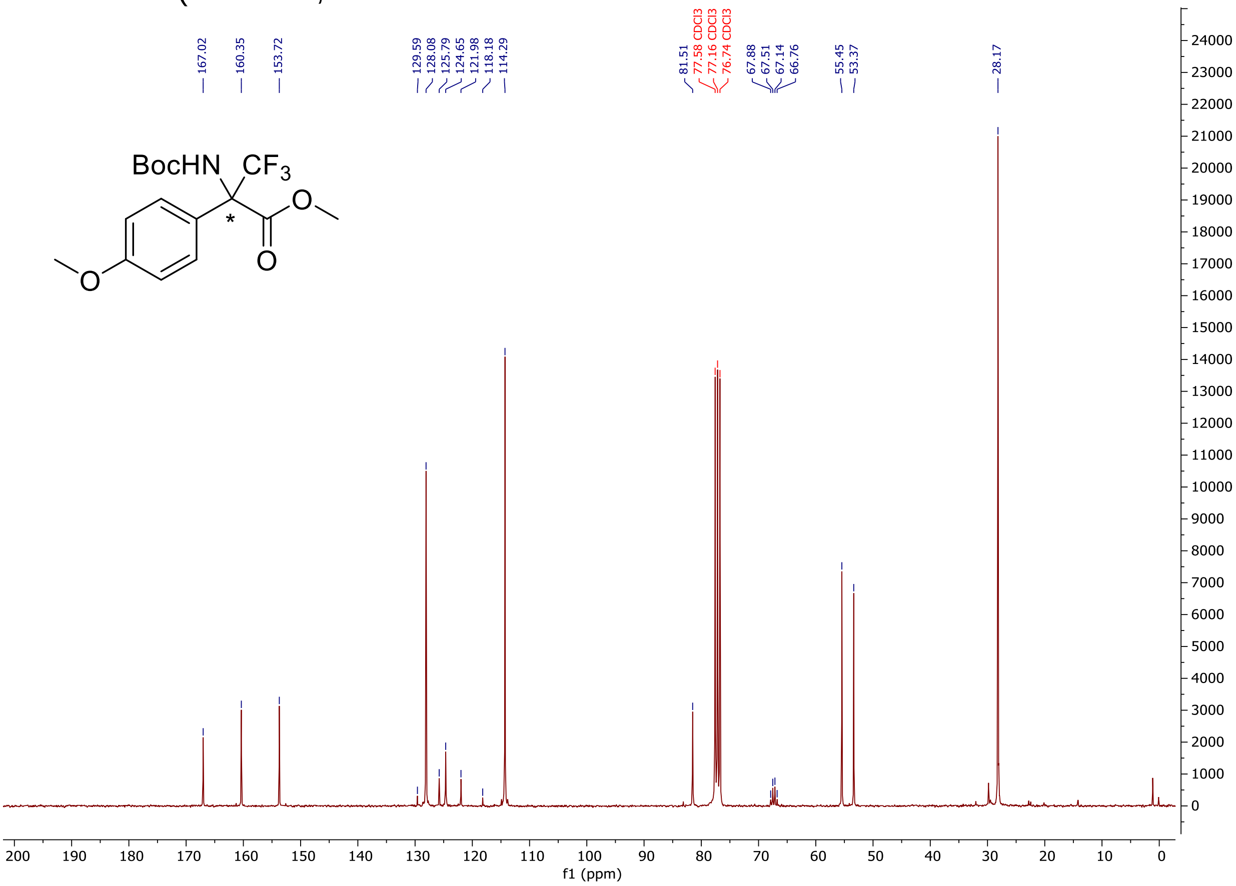

methyl 2-((tert-butoxycarbonyl)amino)-2-(4-(tert-butyl)phenyl)-3,3,3-trifluoropropanoate (17d)

^{1}H-NMR (400 MHz;

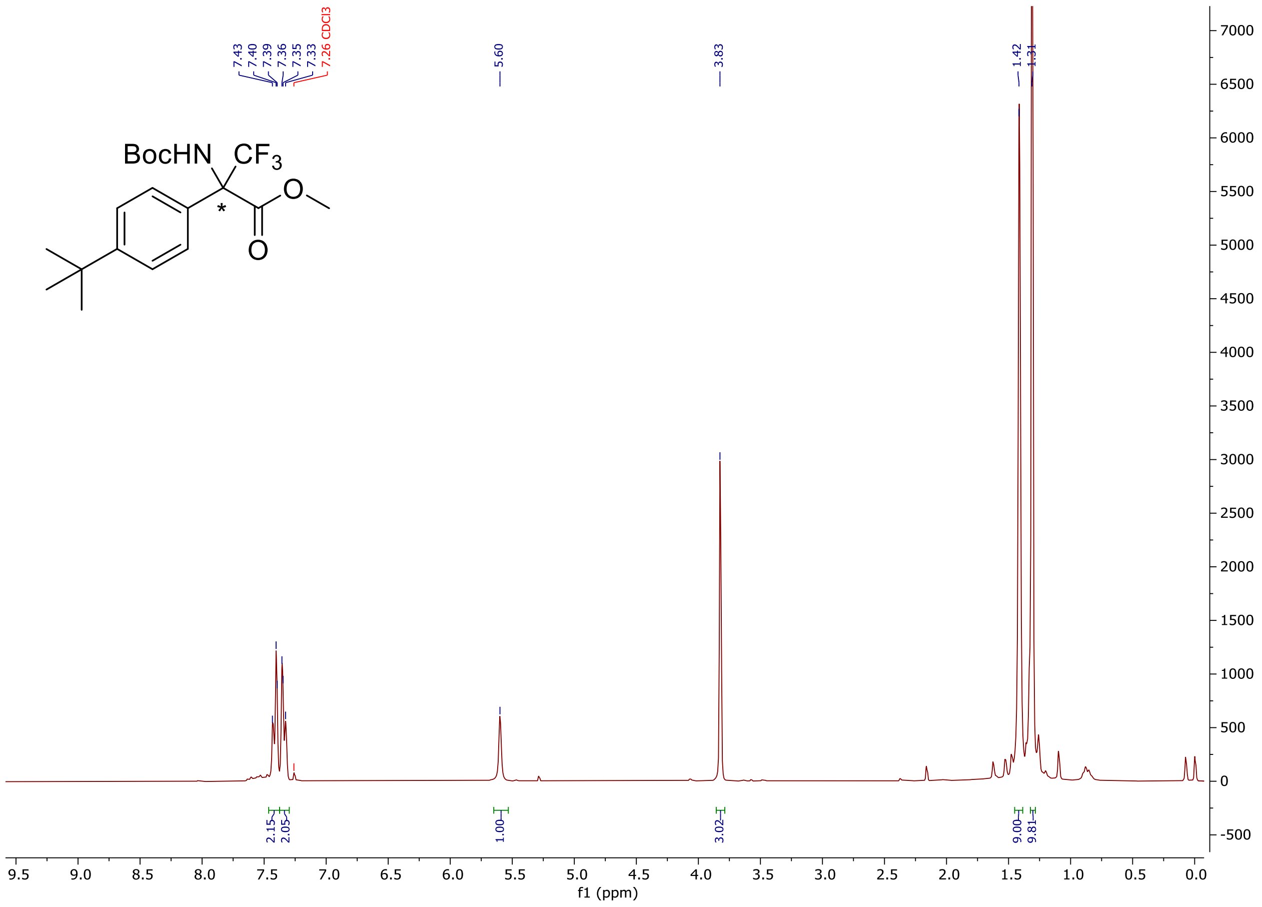

^{19}F-NMR (282 MHz;

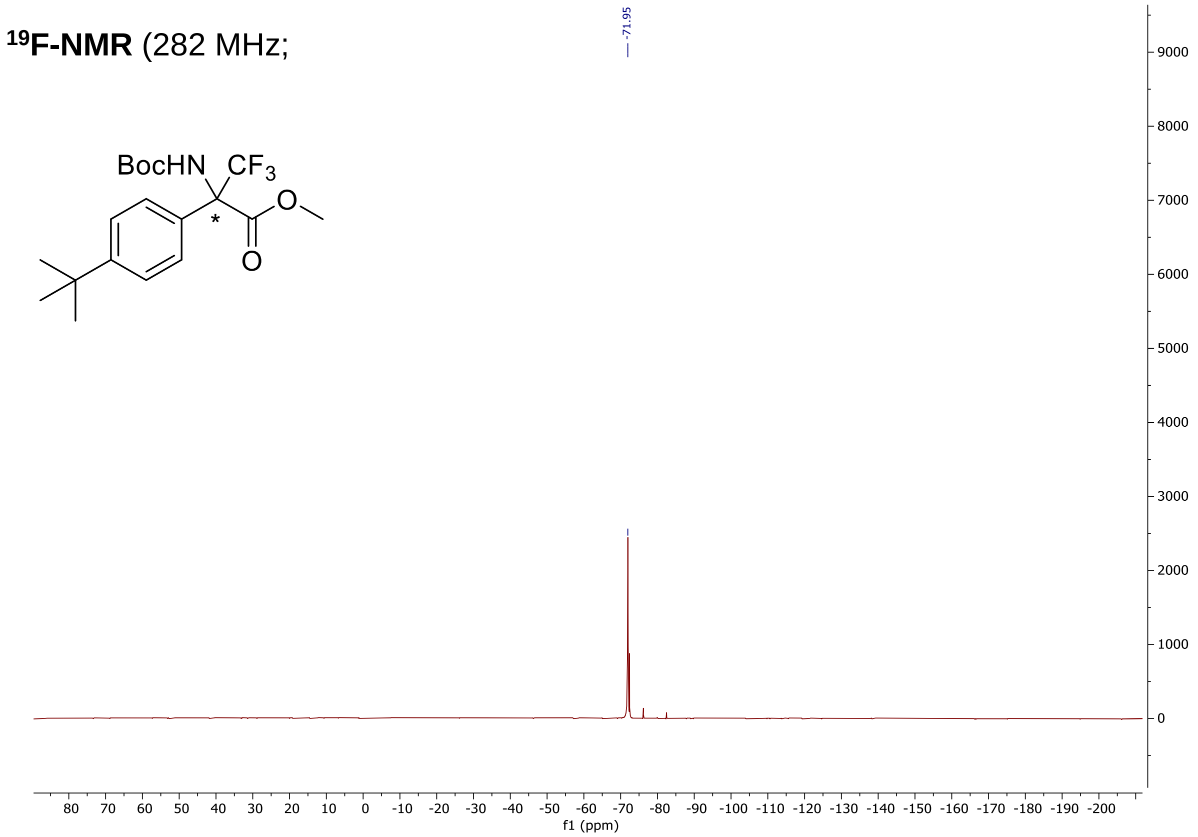

^{13}C-NMR (101 MHz;

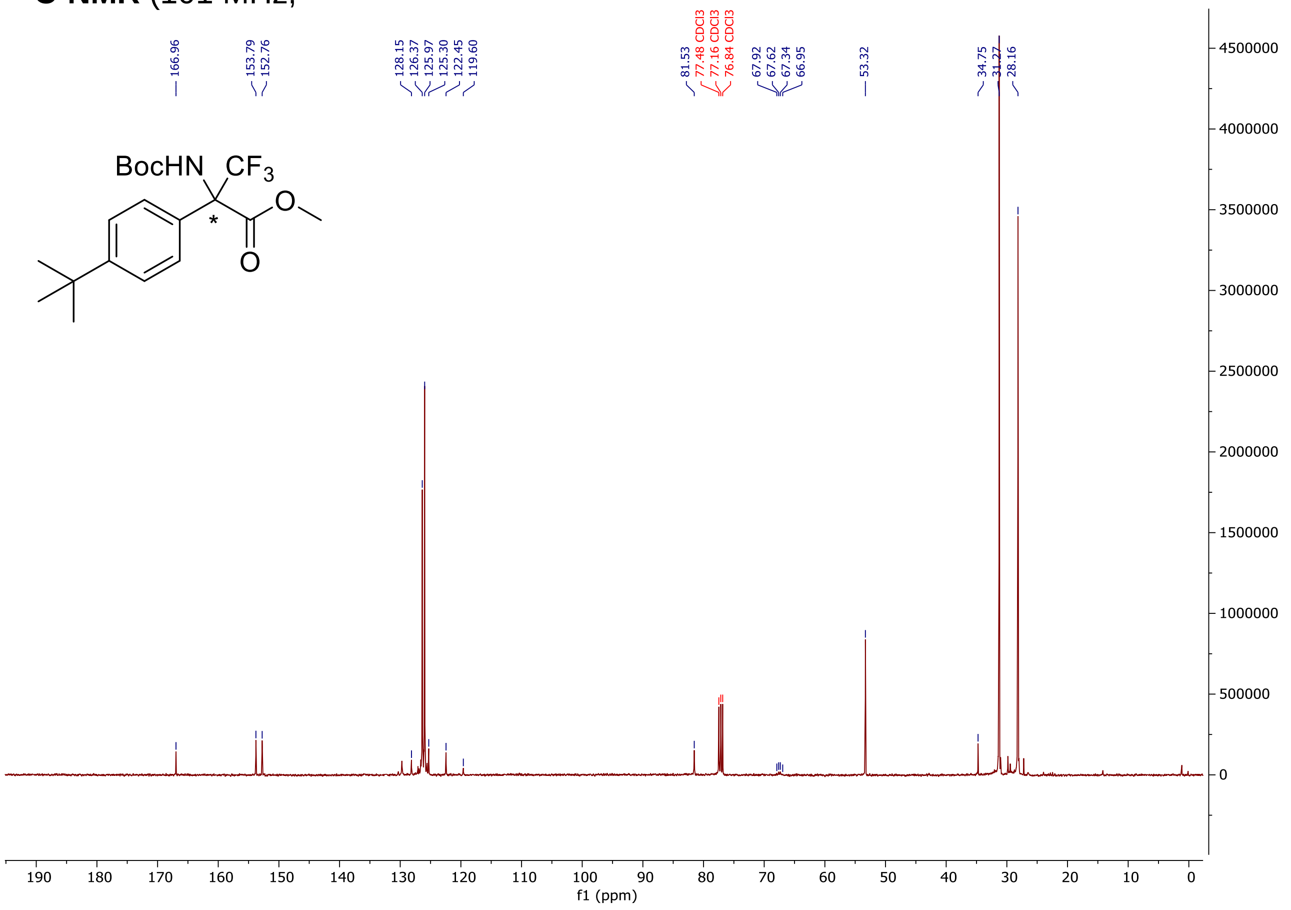

methyl 2-((tert-butoxycarbonyl)amino)-3,3,3-trifluoro-2-(m-tolyl)propanoate (17e)

^{1}H-NMR (300 MHz;

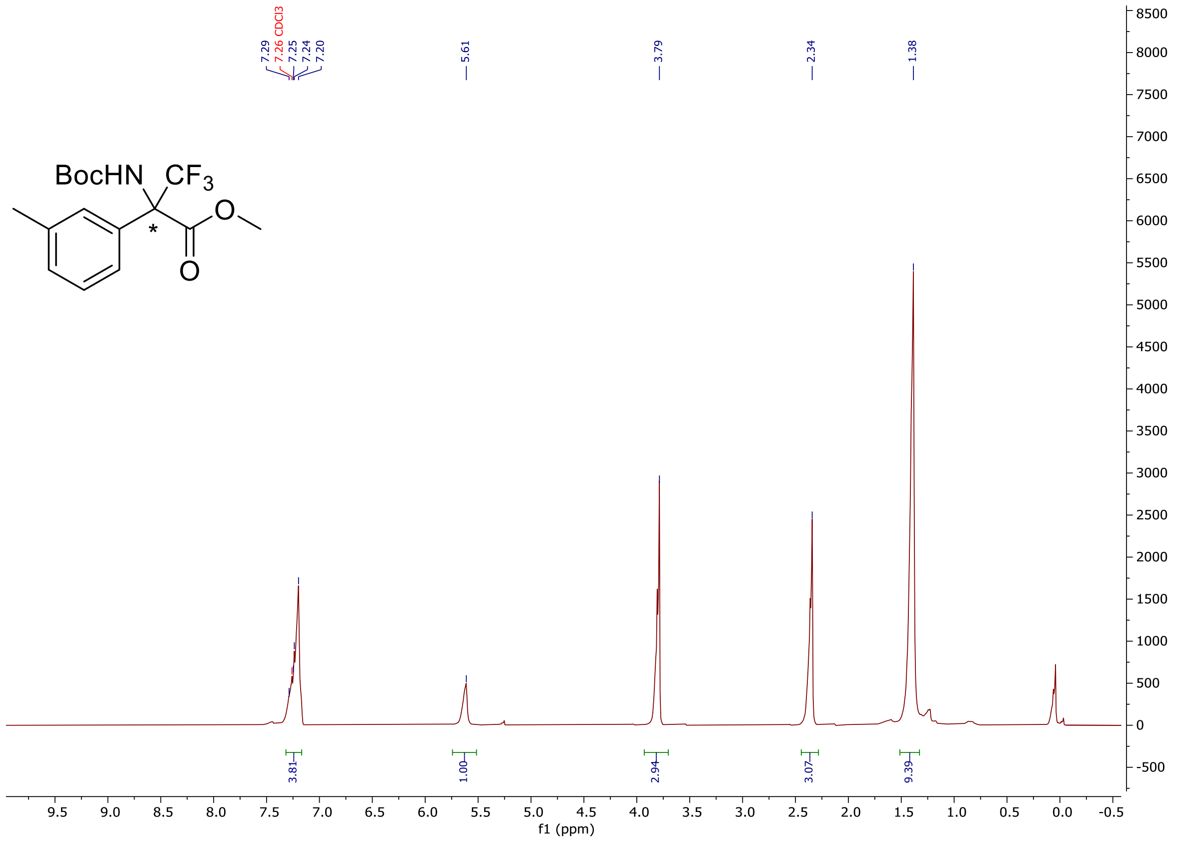

^{19}F-NMR (282 MHz; $CDCl_3$)

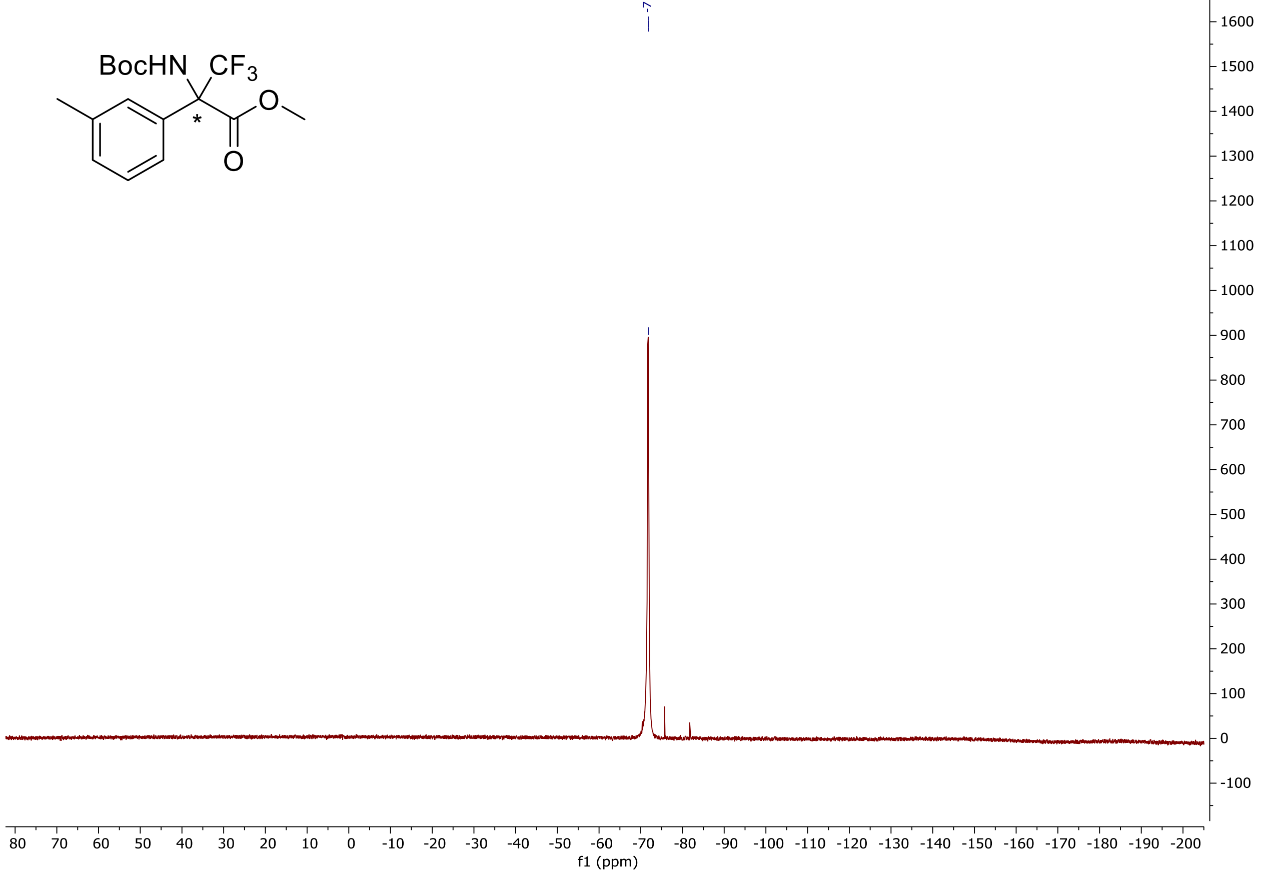

^{13}C-NMR (101 MHz;

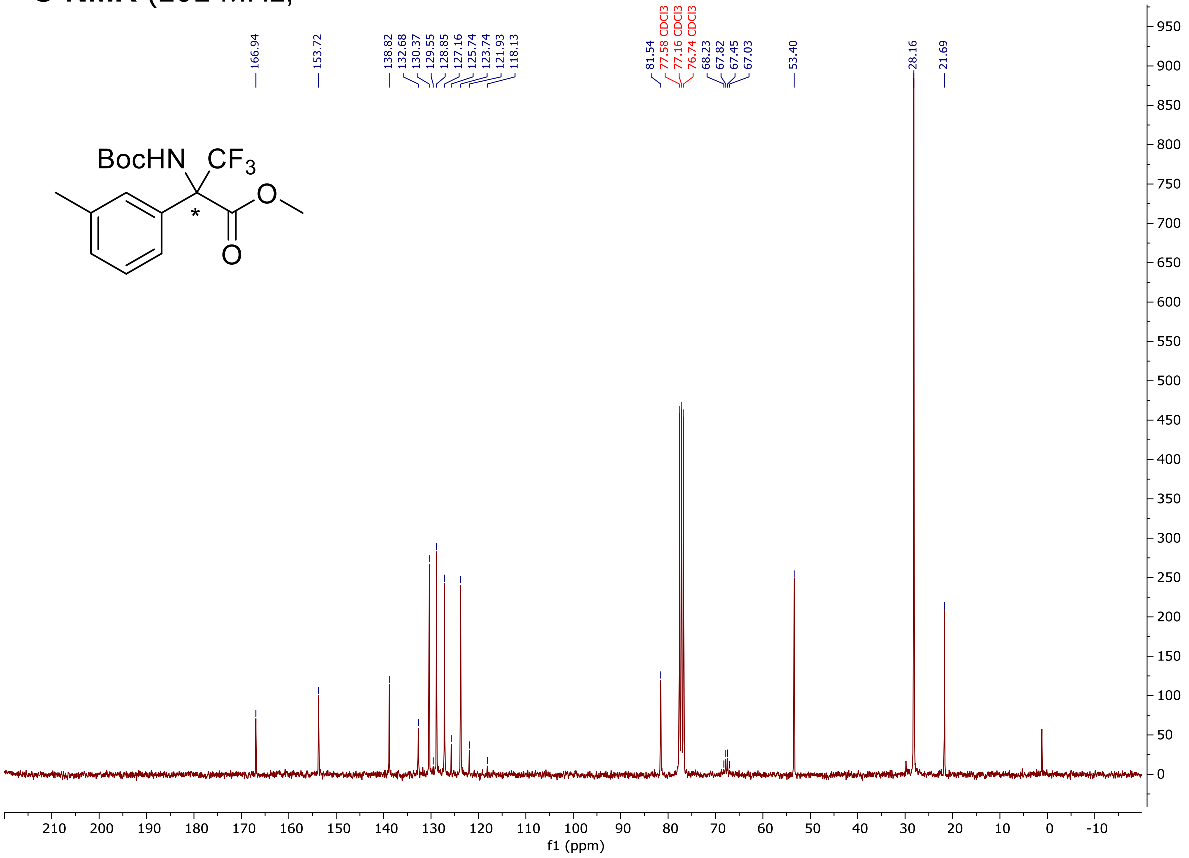

methyl 2-((tert-butoxycarbonyl)amino)-2-(3-chloro-5-fluorophenyl)-3,3,3-trifluoropropanoate (17f) **^{1}H-NMR** (300 MHz;

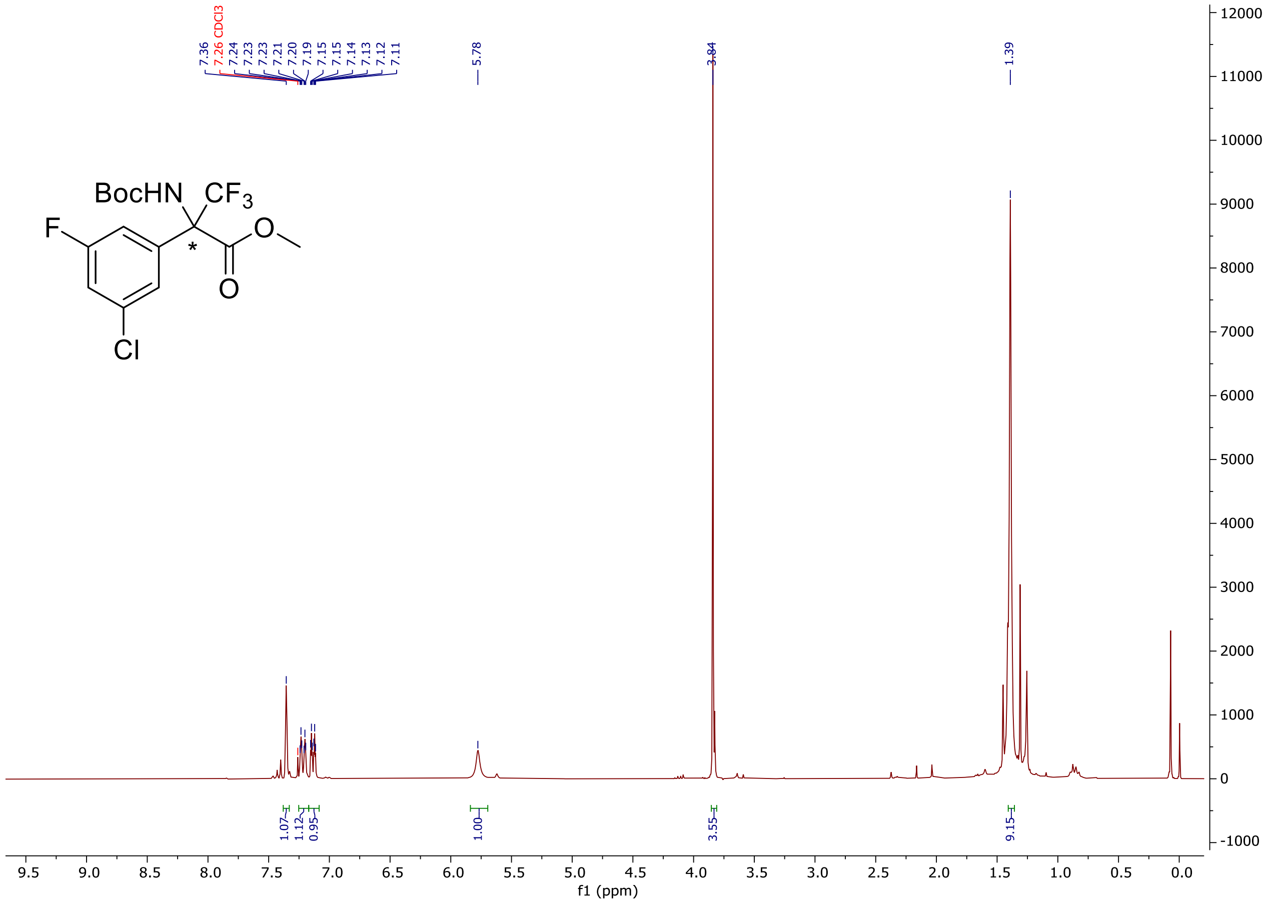

^{19}F-NMR (282 MHz; $CDCl_3$)

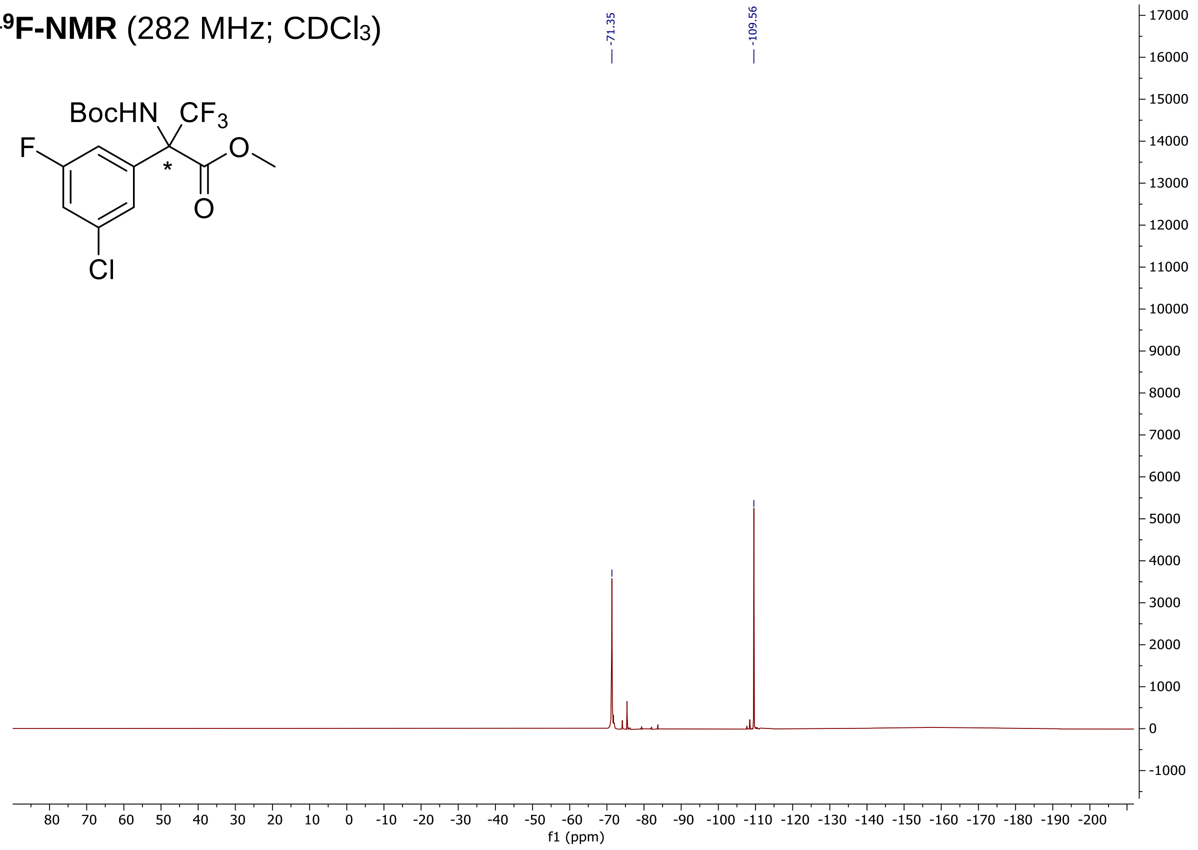

^{13}C-NMR (101 MHz;

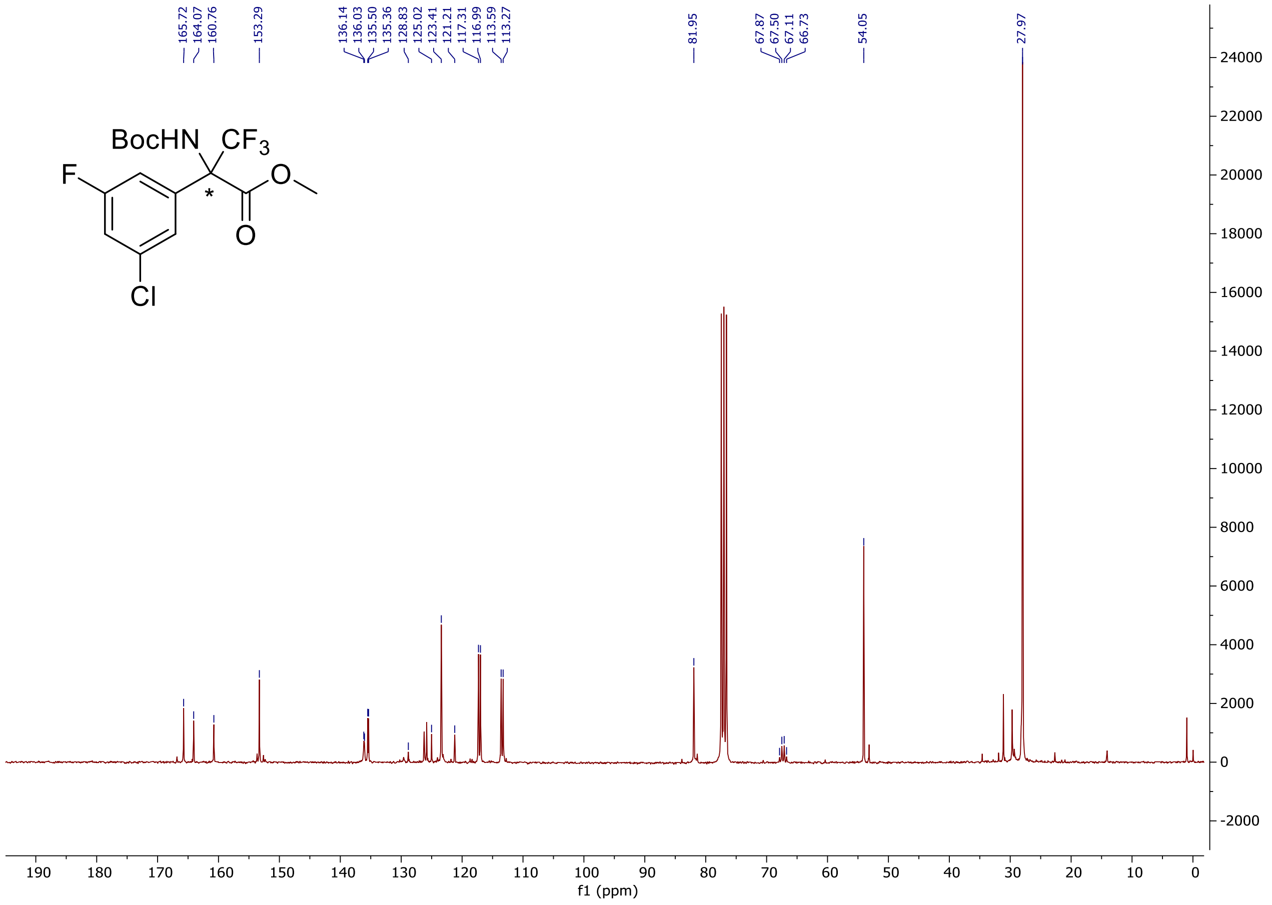

methyl 3-((tert-butoxycarbonyl)amino)-3-(4-chlorophenyl)-2-cyano-4,4,4-trifluorobutanoate (20) **^{1}H-NMR** (400 MHz;

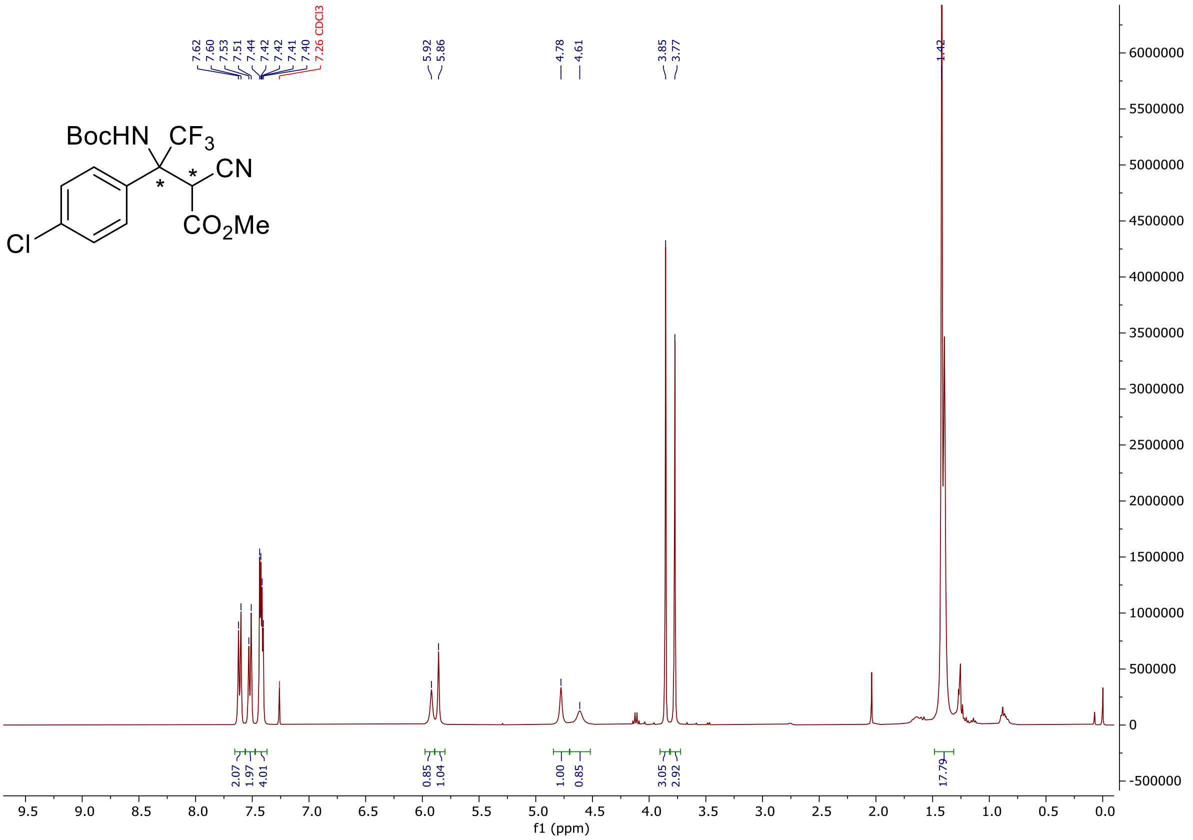

^{19}F-NMR (282 MHz;

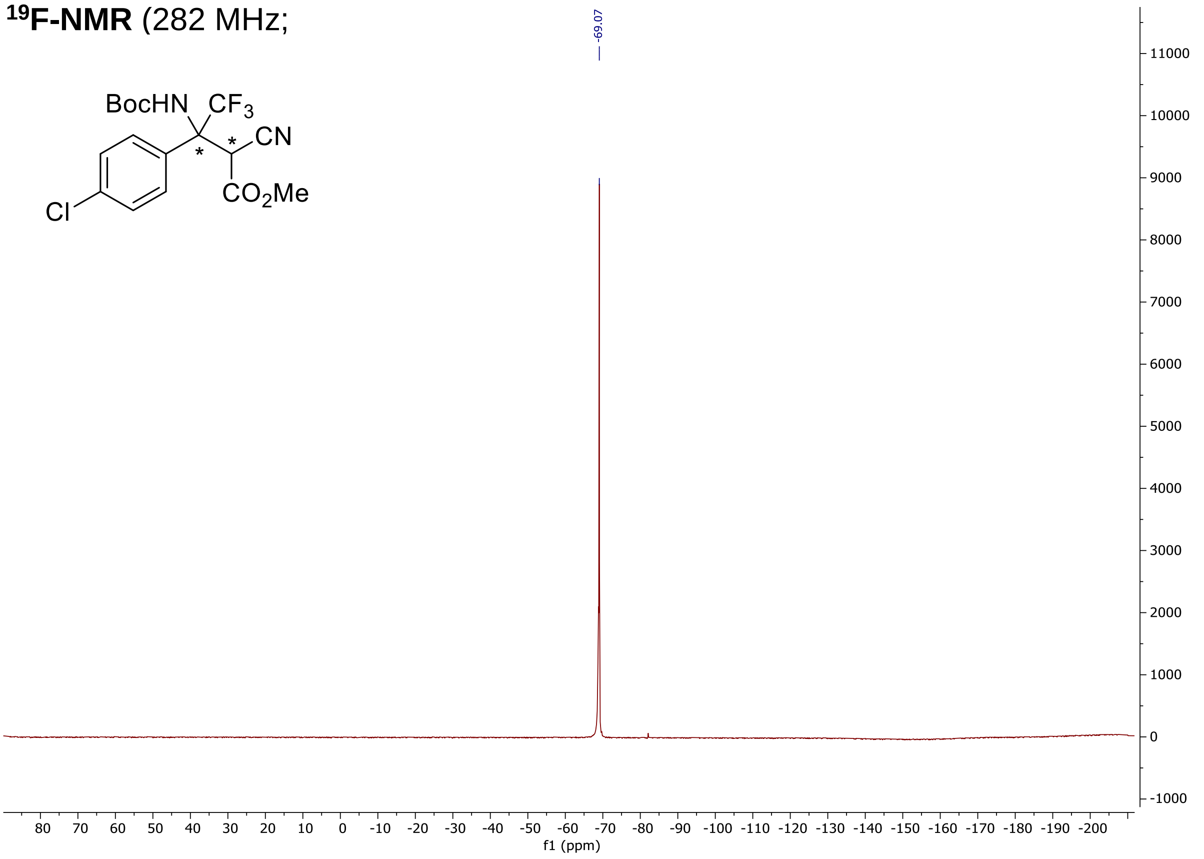

^{13}C-NMR (101 MHz;

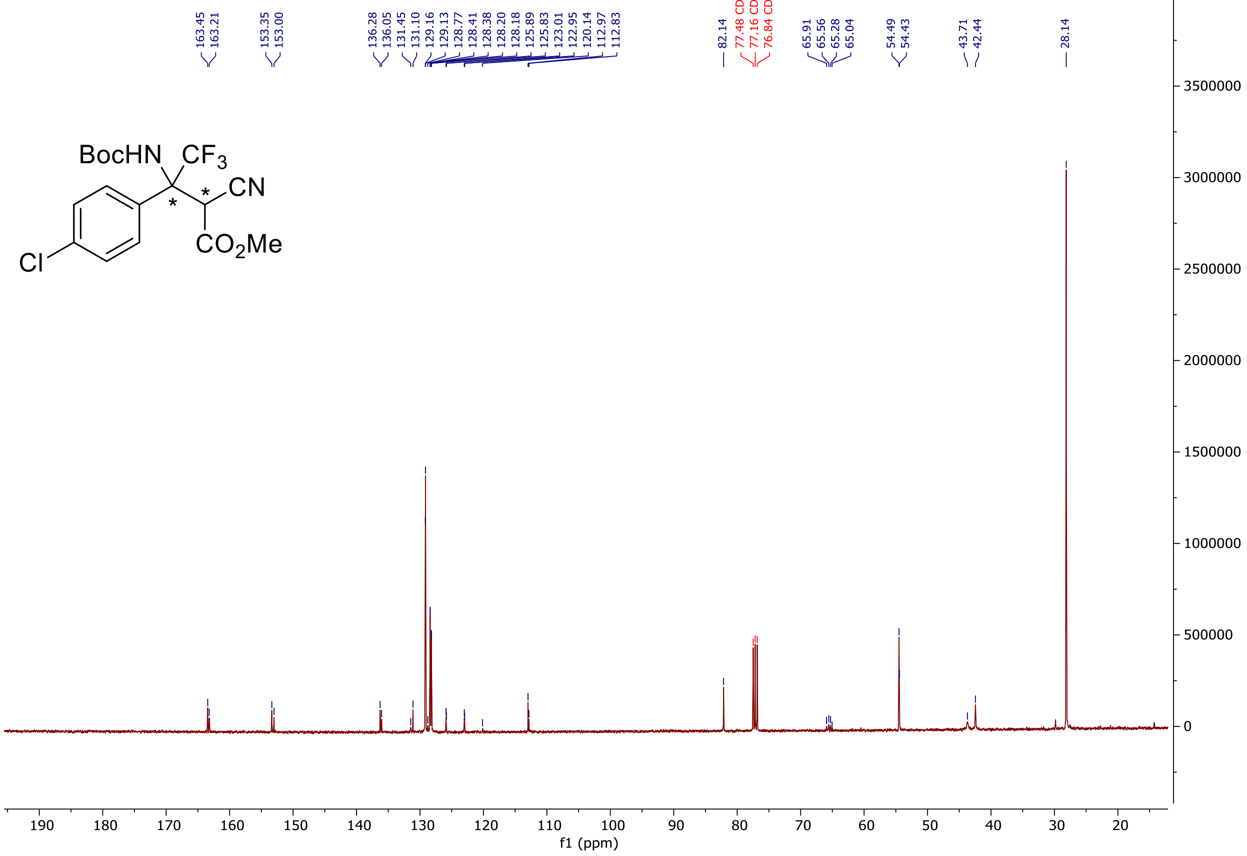

COSY ($CDCl_3$)

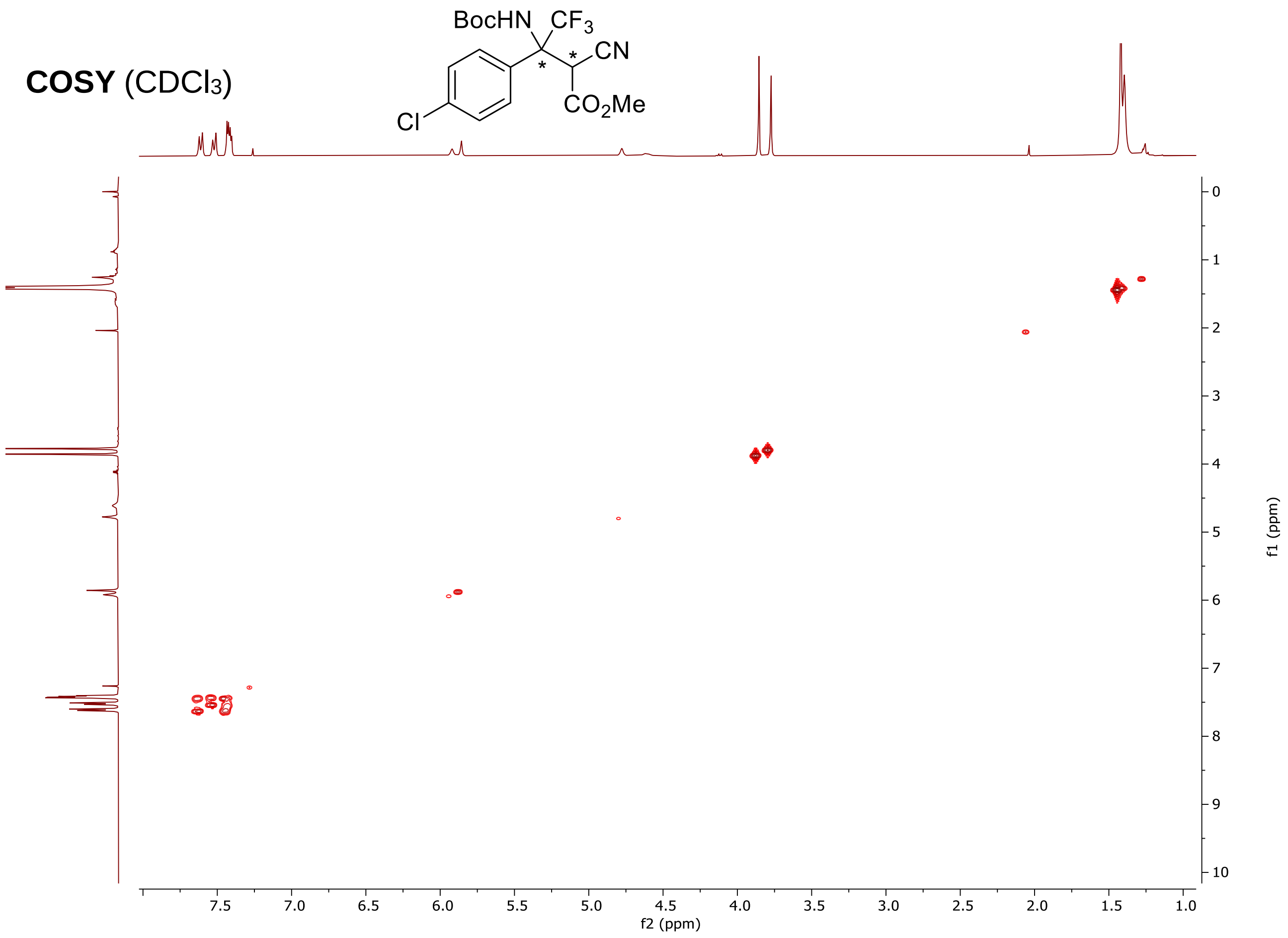

HSQC ($CDCl_3$)

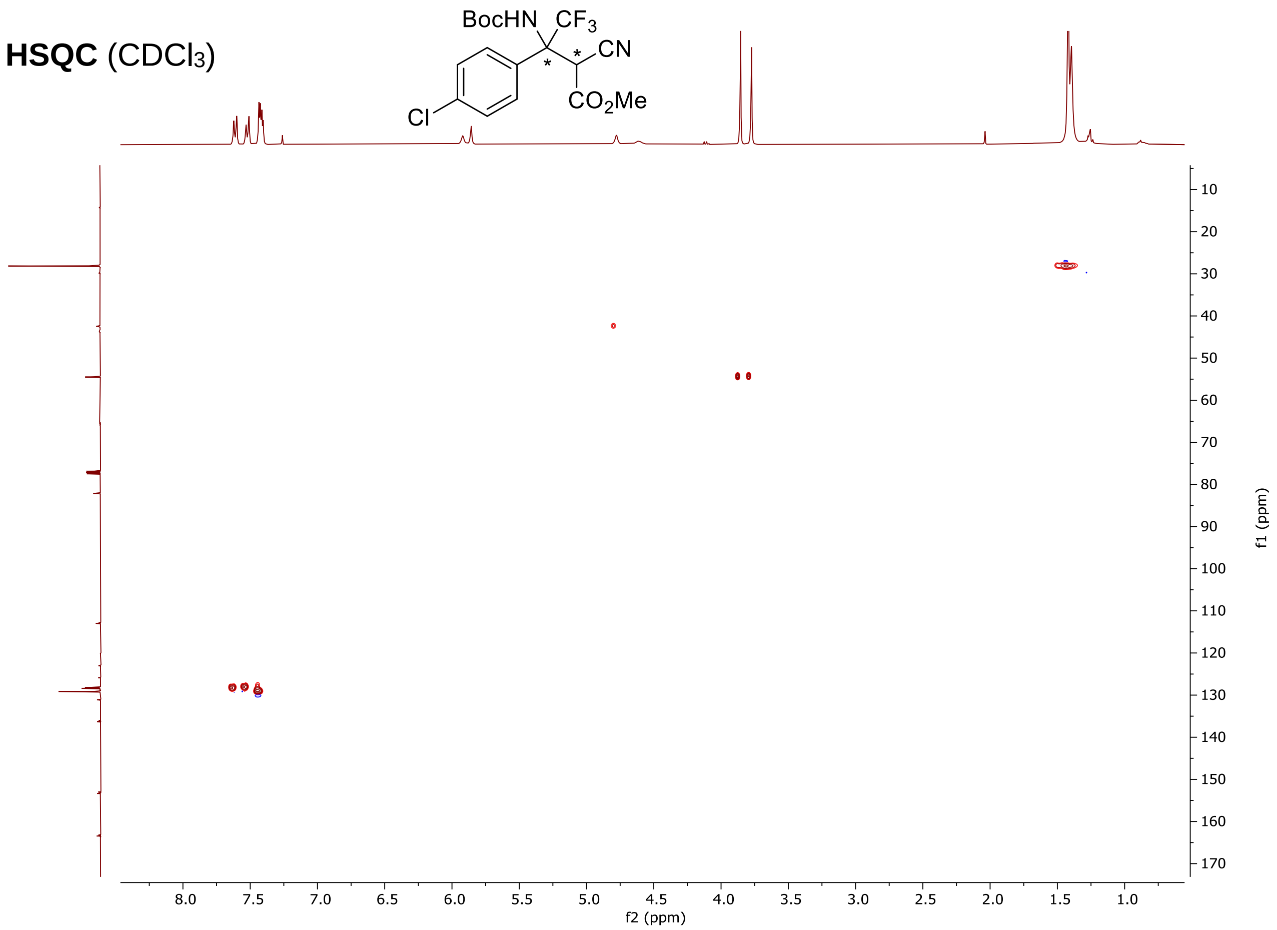

HMBC (CDCl3)

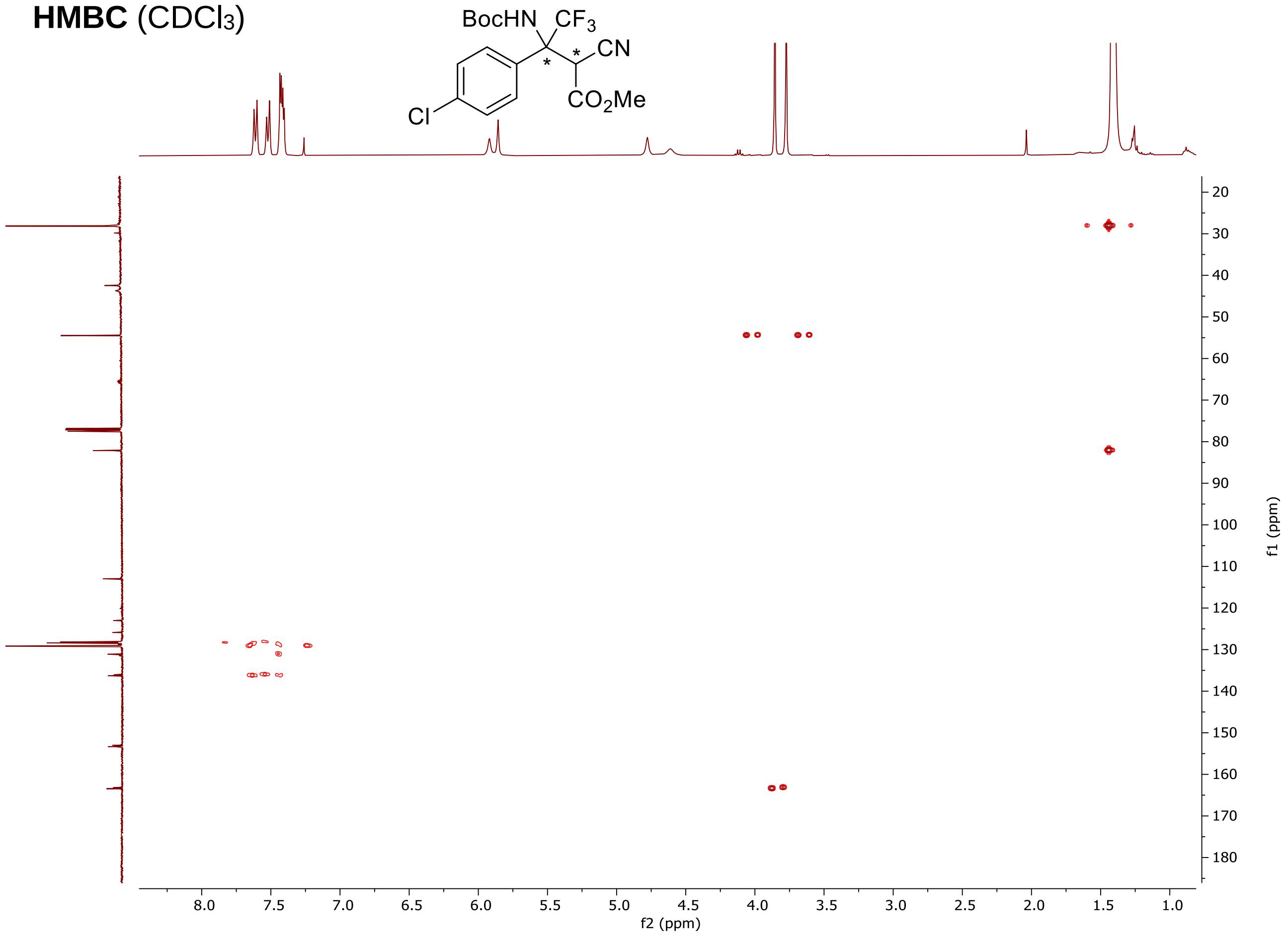

4. HPLC traces

methyl 2-((tert-butoxycarbonyl)amino)-3,3,3-trifluoro-2-phenylpropanoate (17a)

lUX AMYLOSE 1, 95:5, Hex:IPA, 1 ml/min, 94 bar

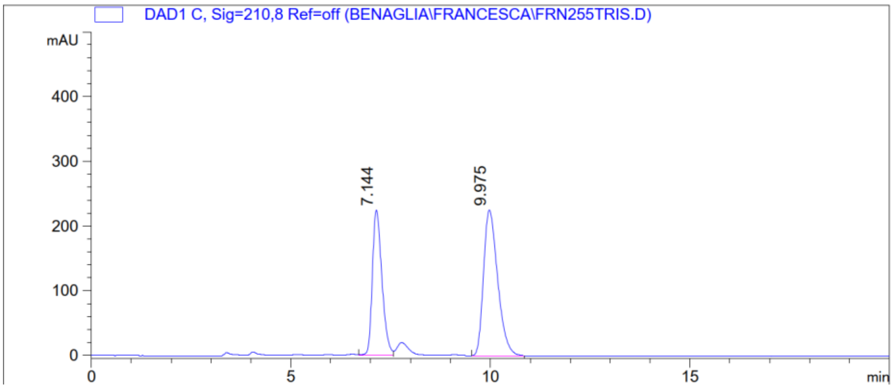

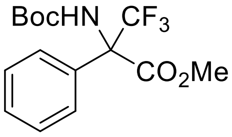

racemate

#	Meas.RT	Main Peak	Main Peak	Main Peak	Main Peak
1	7.144	0.257	3745.015	224.328	40.379
2	9.975	0.379	5529.624	224.935	59.621

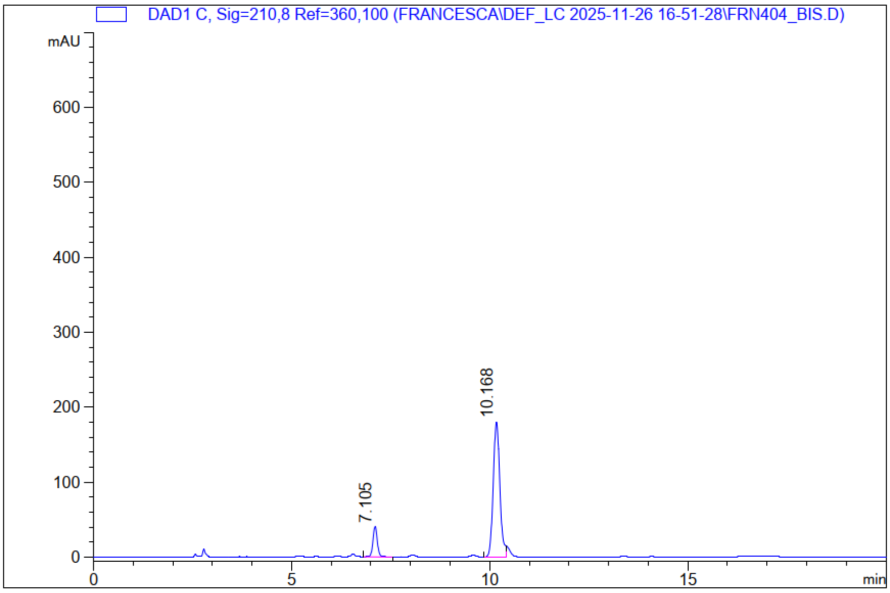

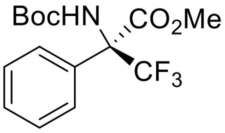

Signal 1: DAD1 C, Sig=210,8 Ref=360,100

Peak #	RT [min]	Type	Width [min]	Area	Area %	Name
1	7.105	BB	0.120	310.161	12.814	
2	10.168	MF	0.195	2110.339	87.186	

methyl 2-((tert-butoxycarbonyl)amino)-2-(4-chlorophenyl)-3,3,3-trifluoropropanoate (17b)

CHIRALPAK AD, 95:5 Hex:IPA, 1 ml/min, 36 bar

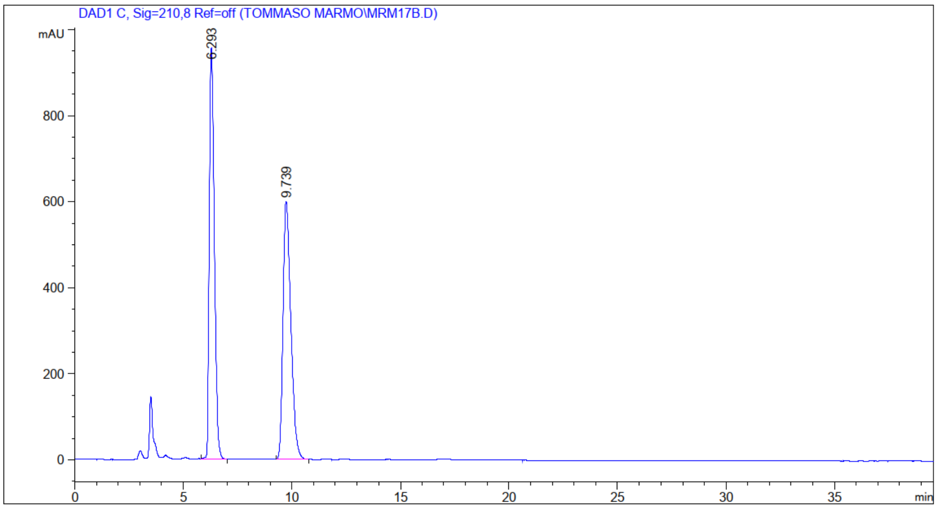

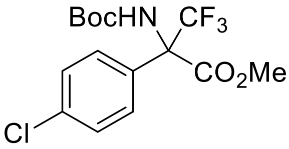

racemate

Peak #	RetTime [min]	Type	Width [min]	Area [mAU*s]	Height [mAU]	Area %
1	6.293	VB	0.2466	1.51106e4	954.84796	49.9328
2	9.739	BB	0.3889	1.51512e4	599.42786	50.0672
Totals :				3.02618e4	1554.27582	

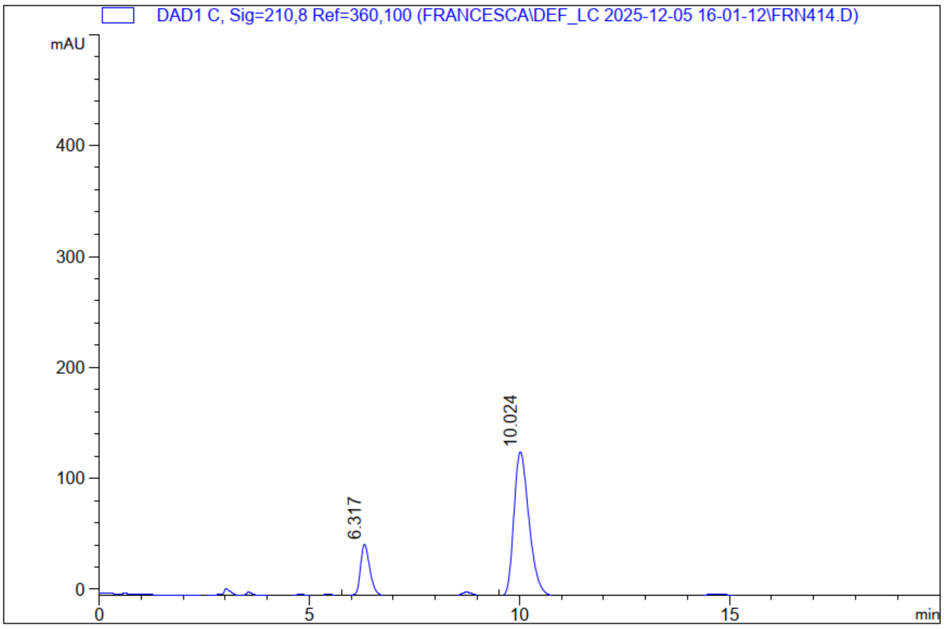

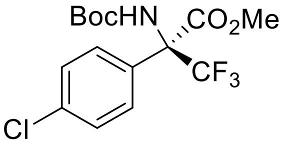

Signal 1: DAD1 C, Sig=210,8 Ref=360,100

Peak #	RT [min]	Type	Width [min]	Area	Area %	Name
1	6.317	BB	0.241	738.556	18.498	
2	10.024	BB	0.384	3254.090	81.502	

methyl 2-((tert-butoxycarbonyl)amino)-3,3,3-trifluoro-2-(4-methoxyphenyl)propanoate (17c)

Chiralpak AD, 95:5 Hex IPA, 1 ml/min,29 bar

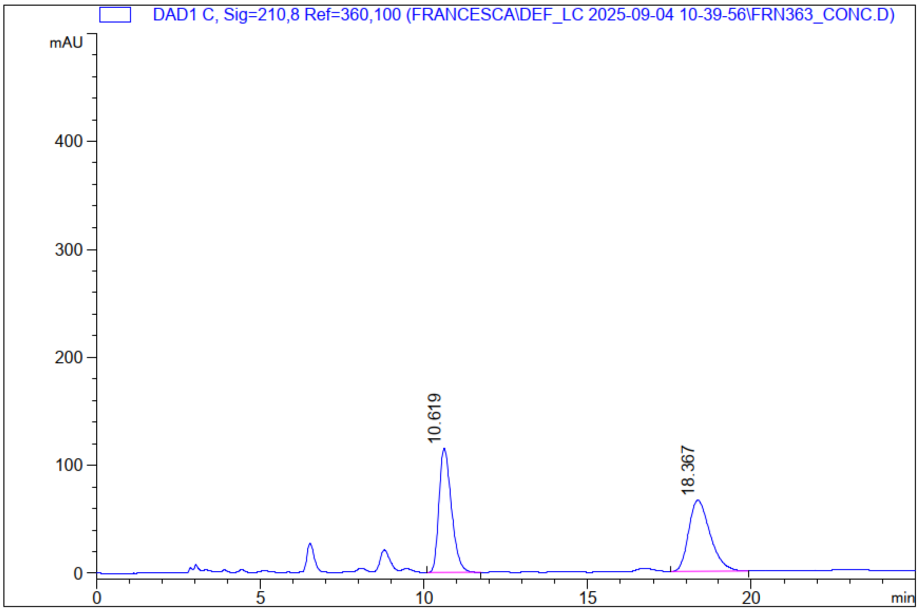

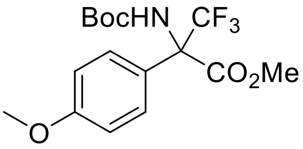

racemate

Signal 1: DAD1 C, Sig=210,8 Ref=360,100

Peak #	RT [min]	Type	Width [min]	Area	Area %	Name
1	10.619	BB	0.406	3030.243	50.217	
2	18.367	BB	0.704	3004.057	49.783	

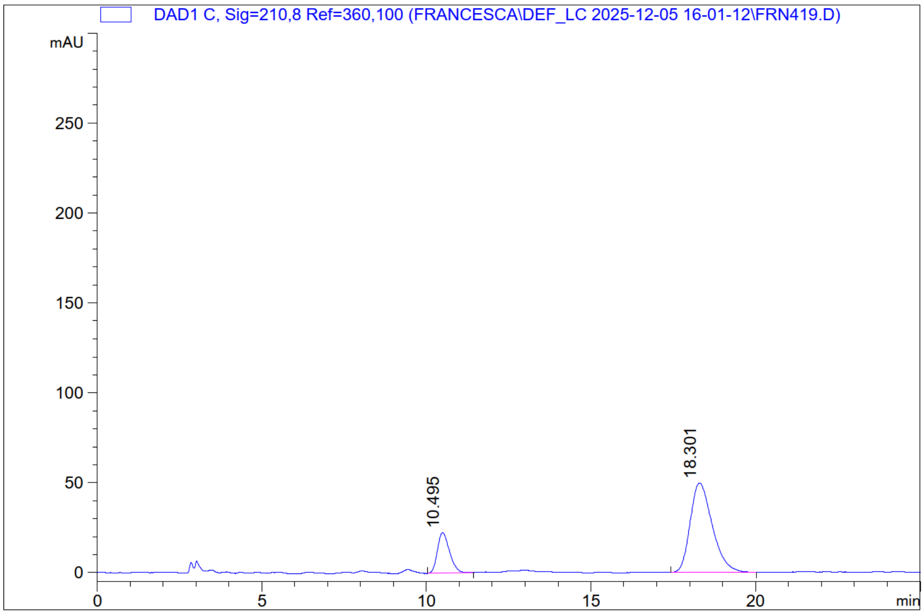

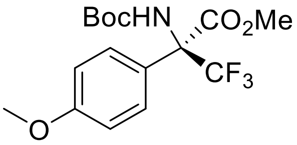

Signal 1: DAD1 C, Sig=210,8 Ref=360,100

Peak #	RT [min]	Type	Width [min]	Area	Area %	Name
1	10.495	BB	0.394	576.454	19.884	
2	18.301	BB	0.704	2322.593	80.116	

ethyl 2-((tert-butoxycarbonyl)amino)-2-(4-(tert-butyl)phenyl)-3,3,3-trifluoropropanoate (17d)

lux phenomenex 3um amylose-1, 95:5, Hex:IPA, 1 ml/min, 90 bar

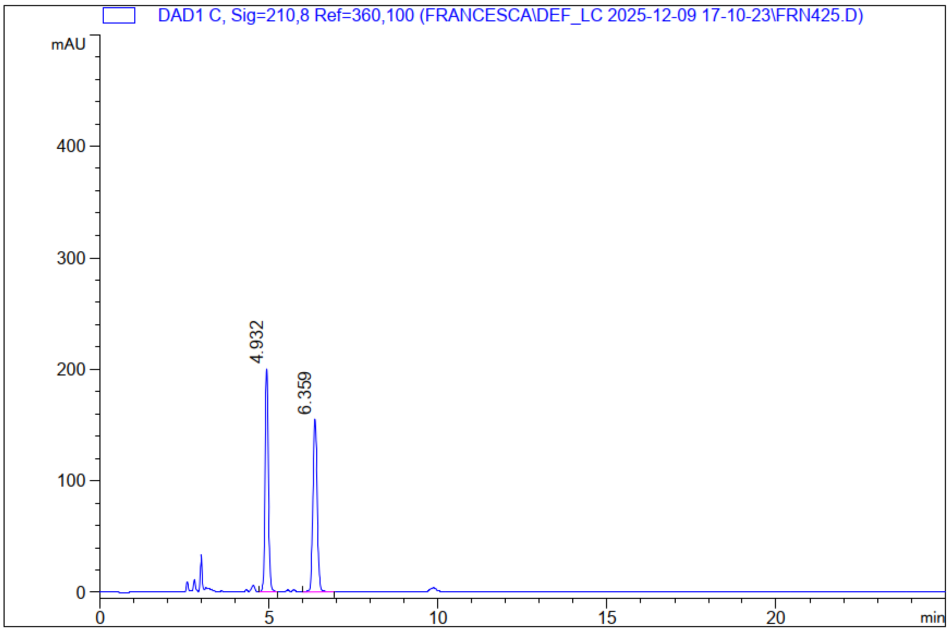

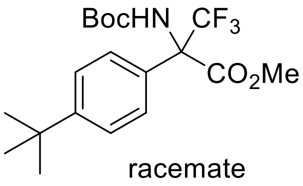

Signal 1: DAD1 C, Sig=210,8 Ref=360,100

Peak #	RT [min]	Type	Width [min]	Area	Area %	Name
1	4.932	BV	0.108	1377.464	50.013	
2	6.359	BB	0.140	1376.739	49.987	

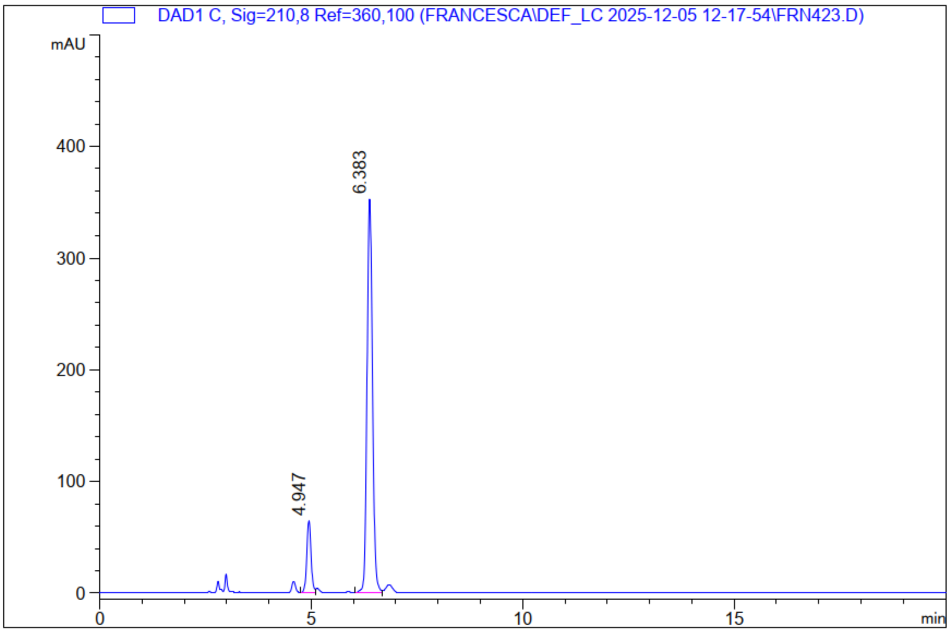

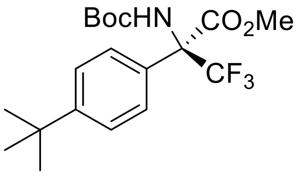

Signal 1: DAD1 C, Sig=210,8 Ref=360,100

Peak #	RT [min]	Type	Width [min]	Area	Area %	Name
1	4.947	VV	0.108	451.509	12.518	
2	6.383	VV	0.140	3155.365	87.482	

methyl 2-((tert-butoxycarbonyl)amino)-3,3,3-trifluoro-2-(m-tolyl)propanoate (17e)

lUX AMYLOSE 1, 95:5, Hex:IPA, 1 ml/min, 94 bar

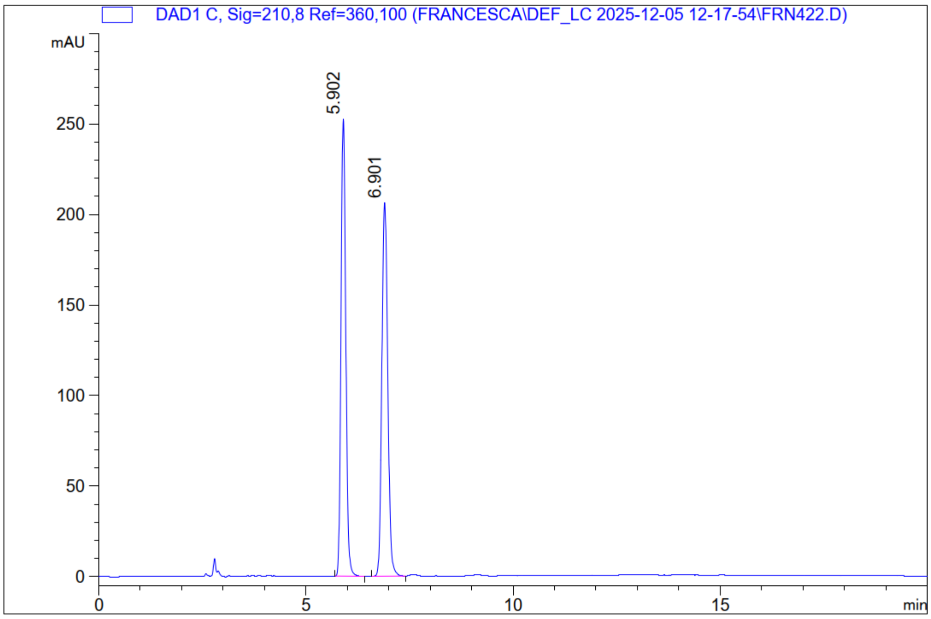

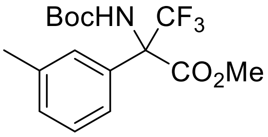

racemate

Signal 1: DAD1 C, Sig=210,8 Ref=360,100

Peak #	RT [min]	Type	Width [min]	Area	Area %	Name
1	5.902	BB	0.126	2032.719	49.783	
2	6.901	BB	0.156	2050.457	50.217	

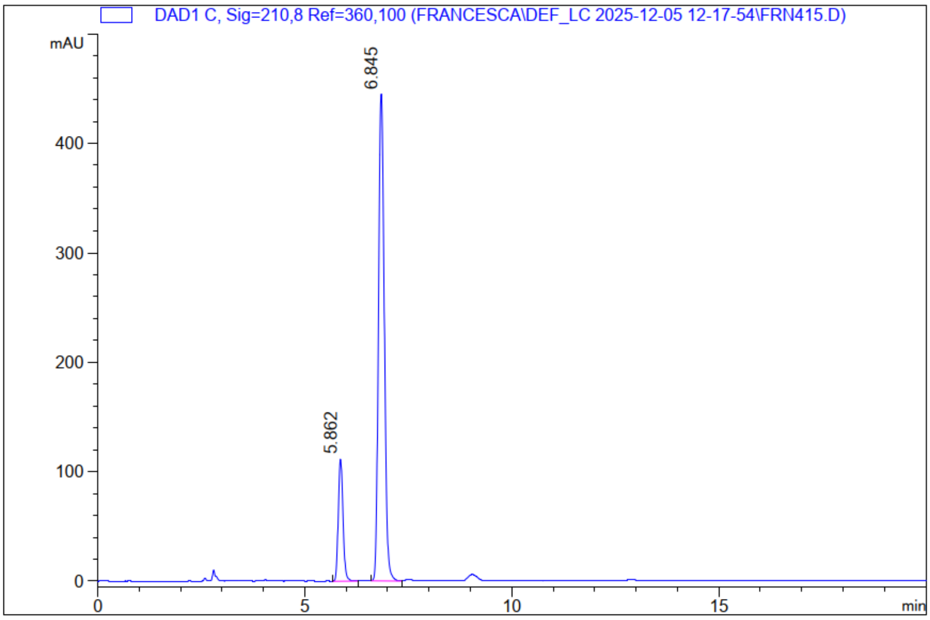

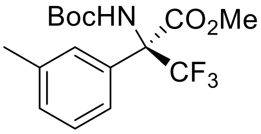

Peak #	RT [min]	Type	Width [min]	Area	Area %	Name
1	5.862	BB	0.125	890.111	16.996	
2	6.845	BV	0.154	4347.144	83.004	

methyl 2-((tert-butoxycarbonyl)amino)-2-(3-chloro-5-fluorophenyl)-3,3,3-trifluoropropanoate (17f)

lux 3um amylose-1, 95:5, Hex:IPA, 1 ml/min, 92 bar

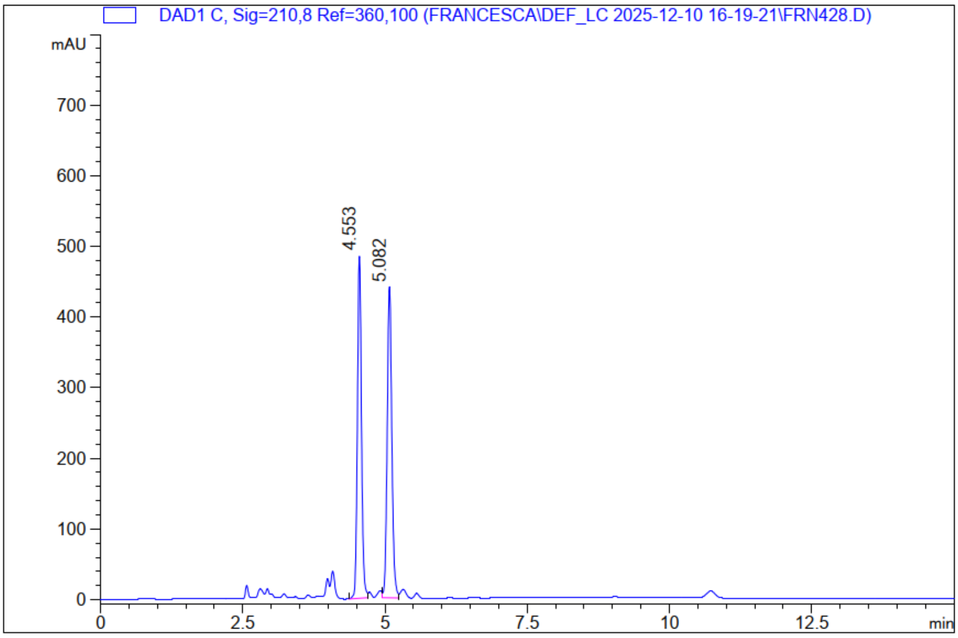

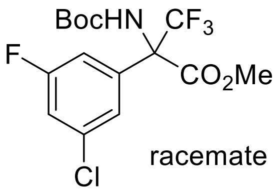

Signal 1: DAD1 C, Sig=210,8 Ref=360,100

Peak #	RT [min]	Type	Width [min]	Area	Area %	Name
1	4.553	BV	0.078	2416.384	49.121	
2	5.082	VV	0.088	2502.865	50.879	

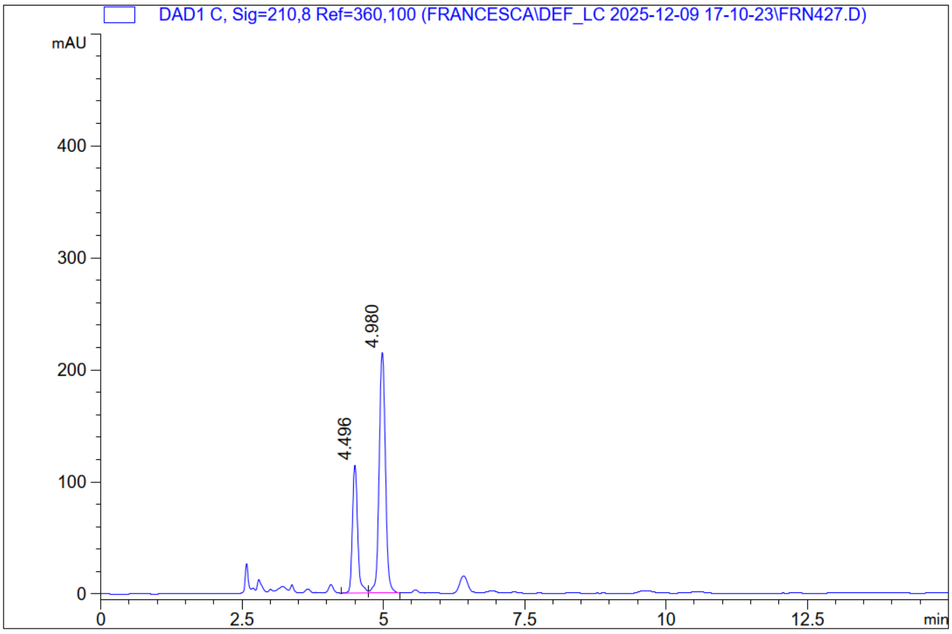

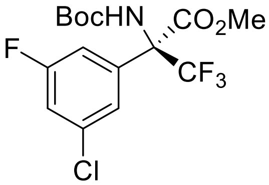

Signal 1: DAD1 C, Sig=210,8 Ref=360,100

Peak #	RT [min]	Type	Width [min]	Area	Area %	Name
1	4.496	BV	0.099	737.482	30.784	
2	4.980	VB	0.122	1658.173	69.216	

5. Computational data

5.1 Micro-pKa determination of catalyst 1a

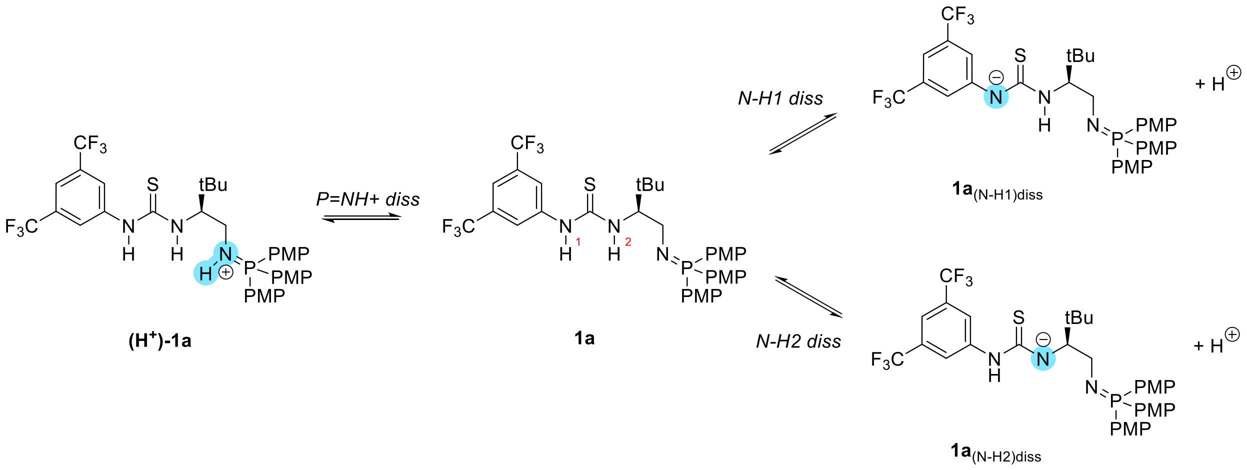

Scheme S1: Catalyst **1a** dissociation profile

The initial conformational geometries were obtained through Monte Carlo conformational analysis performed with Molecular Mechanics calculations using the OPLS4 force field[11] of the Macromodel package[12] in the Schrodinger suite.[13] For molecules exhibiting multiple conformers, the structure within 3 kcal/mol were fully optimized by DFT calculations in gas-phase using the M06-2X functional[14] with the 6-31+G(d) basis set implemented in the Gaussian package.[15] The Gibbs free energies of the protonated catalyst and the deprotonated species (see **Scheme S1**) were recalculated through full geometry optimization and vibrational frequency analysis at the SMD/M06-2X/6-311++G(d,p) level of theory. The resulting pKa values, corrected using the linear free energy scaling relationships,[16] were computed in both DMSO and CH_3CN as solvent ($\Delta G(H^+)_{(DMSO)}$ = -11.1155416306813 eV, ($\Delta G(H^+)_{(CH3CN)}$ = -11.0855416306813 eV). Calculated free Gibbs energies are reported in **Table S1**.

Compound	Free Gibbs energies in DMSO (hartree)	Free Gibbs energies in CH_3CN (hartree)
**(H^+)-1a**	-3122.891736	-3122.90017
**1a**	-3122.433395	-3122.44272
**1a**$_{(N\text{-}H1)diss}$	-3121.957327	-3121.964745
**1a**$_{(N\text{-}H2)diss}$	-3121.947056	-3121.956079

Table S1: Free Gibbs energies calculated at SMD/M06-2X/6-311++G(d,p) level of theory.

5.2 Transition state investigation

Conformational searches of the preliminary geometries of (protonated catalyst **(H^+)-1a**):(ketimine **12a**):(malononitrile anion **15'**) complexes were performed employing a Monte Carlo sampling approach, using the OPLS4 molecular mechanics force field as implemented in the Macromodel module of the Schrödinger suite. The lowest-energy thus obtained conformers (below 3.0 kcal/mol) were subjected to geometry optimization, and the Gibbs free energies of the corresponding transition states were estimated through the semiempirical PM6 method.[17]

Initially, a constrained optimization was carried out, fixing the distance between the reaction carbons of ketimine **12a** and compound **15’** to 2.33 Å. After that, the constrain was removed and the structure was fully optimized in search of a first-order saddle point, which displayed an imaginary frequency corresponding to the vibration of the forming C-C bond between the reactants. Harmonic vibrational frequency calculations were carried out at the same level of theory to verify the nature of the transition states, which was confirmed by the presence of a single imaginary frequency. All calculations were performed in the gas phase under standard conditions. In order to ensure to identify all transition-state geometries, five different coordination models were investigated. As a results, 20 different transition states have been located, which are reported in the following tables. The reported $\Delta\Delta G_{rel}$ values are defined exclusively for the comparison among the four lowest-energy transition states within each coordination model under examination, whereas $\Delta\Delta G_{abs}$ are related to **TS20-*(s-trans)-(Z)-si***, taken as the reference state for the comparative analysis of the computed activation free energies among all the coordination models proposed (**Tables S2-S6**).

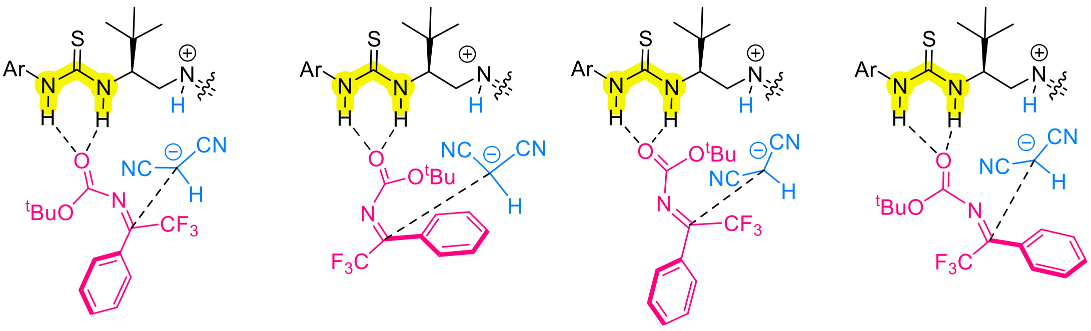

TS1-*(s-cis)-(E)-re* **TS2-*(s-cis)-(E)-si*** **TS3-*(s-cis)-(Z)-re*** **TS4-*(s-cis)-(Z)-si***

	COORDINATION MODEL #1			
	**TS1**	**TS2**	**TS3**	**TS4**
ΔG (hartree)	-0.069394	-0.065921	-0.070118	-0.072866
ΔG (kcal/mol)	-43.5454	-41.3661	-43.9997	-45.7241
$\Delta\Delta G_{rel}$	2.18	4.36	1.72	0.00
$\Delta\Delta G_{abs}$	4.21	6.39	3.75	2.03

Table S2: Computed transition states at PM6 level of theory according to the coordination model #1.

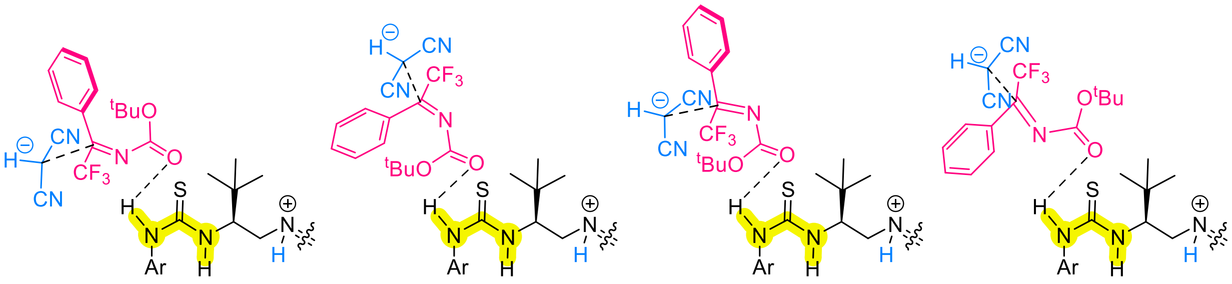

TS5-*(s-trans)-(E)-re* **TS6-*(s-trans)-(E)-si*** **TS7-*(s-trans)-(Z)-re*** **TS8-*(s-trans)-(Z)-si***

	COORDINATION MODEL #2			
	**TS5**	**TS6**	**TS7**	**TS8**
ΔG (hartree)	-0.044287	-0.049973	-0.064494	-0.071781
ΔG (kcal/mol)	-27.7905	-31.3585	-40.4706	-45.0433
$\Delta\Delta G_{rel}$	17.25	13.68	4.57	0.00
$\Delta\Delta G_{abs}$	19.96	16.39	7.28	2.71

Table S3: Computed transition states at PM6 level of theory according to the coordination model #2.

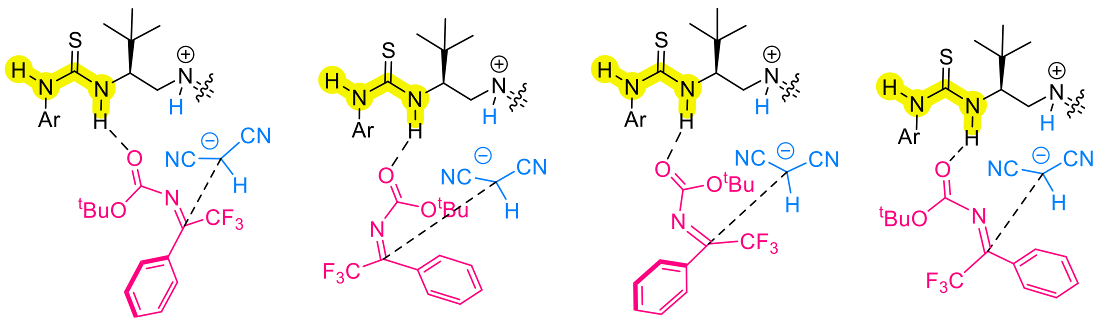

TS9-*(s-trans)-(E)-re* **TS10-*(s-trans)-(E)-si*** **TS11-*(s-trans)-(Z)-re*** **TS12-*(s-trans)-(Z)-si***

	COORDINATION MODEL #3			
	**TS9**	**TS10**	**TS11**	**TS12**
ΔG (hartree)	-0.063955	-0.066961	-0.070477	-0.07359
ΔG (kcal/mol)	-40.1324	-42.0187	-44.2250	-46.1784
$\Delta\Delta G_{rel}$	6.05	4.16	1.95	0.00
$\Delta\Delta G_{abs}$	**7.62**	**5.73**	**3.53**	**1.57**

Table S4: Computed transition states at PM6 level of theory according to the coordination model #3.

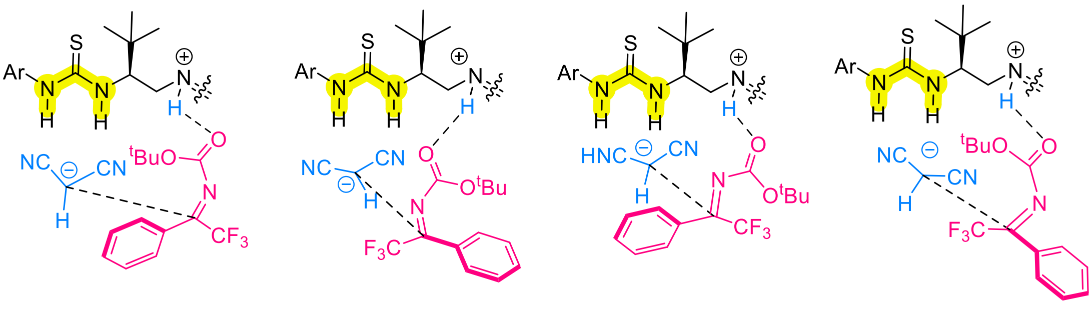

TS13-*(s-cis)-(E)-re* **TS14-*(s-cis)-(E)-si*** **TS15-*(s-cis)-(Z)-re*** **TS16-*(s-cis)-(Z)-si***

	COORDINATION MODEL #4			
	TS13	TS14	TS15	TS16
ΔG (hartree)	-0.06607	-0.066767	-0.06653	-0.065662
ΔG (kcal/mol)	-41.4596	-41.8969	-41.7482	-41.2035
$\Delta\Delta G_{rel}$	0.44	0.00	5.65	6.20
$\Delta\Delta G_{abs}$	**6.29**	**5.86**	**6.00**	**6.55**

Table S5: Computed transition states at PM6 level of theory according to the coordination model #4.

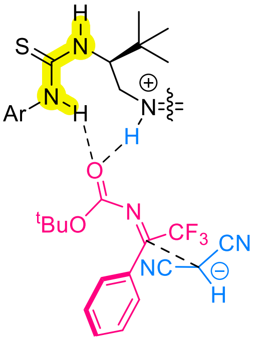

TS17-*(s-trans)-(E)-re*

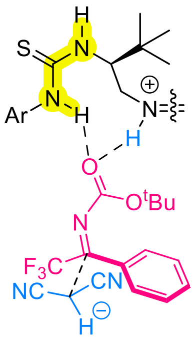

TS18-*(s-trans)-(E)-si*

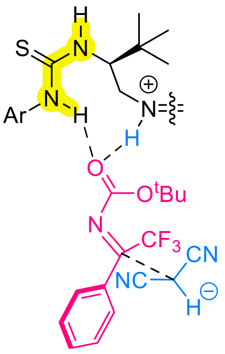

TS19-*(s-trans)-(Z)-re*

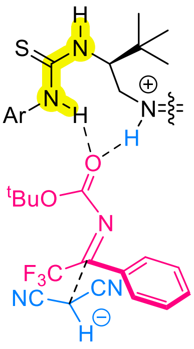

TS20-*(s-trans)-(Z)-si*

	COORDINATION MODEL #5			
	TS17	TS18	TS19	TS20
ΔG (hartree)	-0.075536	-0.067092	-0.063552	-0.076098
ΔG (kcal/mol)	-47.3996	-42.1009	-39.8795	-47.7522
$\Delta\Delta G_{rel}$	0.3527	5.6514	7.8727	0.0000
$\Delta\Delta G_{abs}$	**0.35**	**5.65**	**7.87**	**0.00**

Table S6: Computed transition states at PM6 level of theory according to the coordination model #5.

6. References

[1] Krstić, M.; Benaglia, M.; Gazzotti, M.; Colombo, E.; Sanz. M. *Adv. Synth. Catal*. **2023**, *365*, 1093–1098.

[2] Saitoh, A.; Achiwa, K.; Tanaka, K.; Morimoto T. *J. Org. Chem.* **2000**, *65*, 4227–4240.

[3] Xiao, H.; Chai, Z.; Zheng, C.-W.; Yang, Y.-Q.; Liu, W.; Zhang, J.-K.; Zhao, G. *Angew. Chem. Int. Ed.* **2010**, *49*, 4467–4470.

[4] Kim, J.-M.; Bi, Y.; Paikoff, S. J.; Schultz, P. G. *Tetrahedron Lett.* **1996**, *37*, 5305–5308.

[5] Núñez, M. G.; Farley, A. J. M.; Dixon, D. J. *J. Am. Chem. Soc.* **2013**, *135*, 16348–16351.

[6] Ričko, S.; Svete, J.; Štefane, B.; Perdih, A.; Golobič, A.; Meden, A.; Grošelj, U. *Adv Synth Catal* **2016**, *358*, 3786.

[7] Du, M.; Yu, L.; Du, T.; Li, Z.; Luo, Y.; Meng, X.; Tian, Z.; Zheng, C.; Cao, W.; Zhao, G. *Chem.Commun.* **2020**, *56*, 1581–1584.

[8] Wang, X.; Gao, Y.; Wei, Z.; Cao, J.; Liang, D.; Lin, Y.; Duan, H. *Org. Chem. Front.* **2019**, *6*, 3269–3273.

[9] Liu, Y.; Yun, X.; Zhang-Negrerie, D.; Huang, J.; Du, Y.; Zhao, K. Synthesis of 1,2-Diketones from β-Keto Nitriles via a Protection-Oxidative-Decyanation-Deprotection Protocol. Synthesis 2011, 2011, 2984–2994, doi:10.1055/s-0030-1260156.

[10] Martin, T.; Massif, C.; Wermester, N.; Linol, J.; Tisse, S.; Cardinael, P.; Coquerel, G.; Bouillon, J. P. *Tetrahedron: Asymmetry* **2011**, *22*, 12–21.

[11] Lu, C.; Wu, C.; Ghoreishi, D.; Chen, W.; Wang, L.; Damm, W.; Ross, G. A.; Dahlgren, M. K.; Russell, E.; Von Bargen, C. D.; Abel, R.; Friesner R.A.; Harder, E. D. *J. Chem. Theory Comput*., **2021**, *17*, 4291–4300.

[12] Banks, J. L.; Beard, H. S.; Cao, Y.; Cho, A. E.; Damm, W.; Farid, R.; Felts, A. K.; Halgren, T. A.; Mainz, D. T.; Maple, J. R.; Murphy, R.; Philipp, D. M.; Repasky, M. P.; Zhang, L. Y.; Berne, B. J.; Friesner, R. A.; Gallicchio, E.; Levy. R. M. *J Comput Chem* **2005**, *26*, 1752–1780.

[13] Schrödinger Release 2024-1: MacroModel, Schrödinger, LLC, New York, NY, **2024**.

[14] Zhao Y.; Truhlar, D. G. *Theor. Chem. Acc.*, **2007**, *120*, 215–241.

[15] Gaussian 16, Revision C.01, Frisch, M. J.; Trucks, G. W.; Schlegel, H. B.; Scuseria, G. E.; Robb, M. A.; Cheeseman, J. R.; Scalmani, G.; Barone, V.; Petersson, G. A.; Nakatsuji, H.; Li, X.; Caricato, M.; Marenich, A. V.; Bloino, J.; Janesko, B. G.; Gomperts, R.; Mennucci, B.; Hratchian, H. P.; Ortiz, J. V.; Izmaylov, A. F.; Sonnenberg, J. L.; Williams-Young, D.; Ding, F.; Lipparini, F.; Egidi, F.; Goings, J.; Peng, B.; Petrone A. et al., Gaussian, Inc., Wallingford CT **2019**.

[16] Busch, M.; Ahlberg, E.; Ahlberg E.; Laasonen, K. *ACS Omega*, **2022**, *7*, 17369–17383.

[17] Stewart, J. J. *J Mol Model*, **2007**, *13*, 1173–1213.
